# An integrated “omics” approach to the characterization of maize (*Zea mays L*.) mutants deficient in the expression of two genes encoding cytosolic glutamine synthetase

**DOI:** 10.1186/1471-2164-15-1005

**Published:** 2014-11-20

**Authors:** Nardjis Amiour, Sandrine Imbaud, Gilles Clément, Nicolas Agier, Michel Zivy, Benoît Valot, Thierry Balliau, Isabelle Quilleré, Thérèse Tercé-Laforgue, Céline Dargel-Graffin, Bertrand Hirel

**Affiliations:** Institut Jean-Pierre Bourgin, Institut National de la Recherche Agronomique (INRA), Centre de Versailles-Grignon, Unité Mixte de Recherche 1318 INRA-Agro-ParisTech, Equipe de Recherche Labellisée, Centre National de la Recherche Scientifique 3559, RD10, F-78026 Versailles, Cedex, France; Centre de Génétique Moléculaire, Unité Propre de Recherche 2167, Centre National de la Recherche Scientifique and, Gif/Orsay DNA MicroArray Platform (GODMAP), 1, avenue de la Terrasse, F-91198 Gif sur Yvette Paris, France; Platerforme d’Analyse Protéomique de Paris Sud-Ouest, Unité Mixte de Recherche de Génétique Végétale, Ferme du Moulon, F-91190 Gif/Yvette, Paris, France; Sorbonne Universités, Université Pierre et Marie Curie, Unité Mixte de Recherche 7238, Biologie Computationnelle et Quantitative, Université de Paris 06, F-75006 Paris, France; Centre National de la Recherche Sceintifique, Unité Mixte de Recherche 7238, Biologie Computationnelle et Quantitative, F-75006 Paris, France

**Keywords:** Assimilation, Glutamine synthetase, Grain filling, Maize, Metabolome, Mutant, Nitrogen, Proteome, Transcriptome, Yield

## Abstract

**Background:**

To identify the key elements controlling grain production in maize, it is essential to have an integrated view of the responses to alterations in the main steps of nitrogen assimilation by modification of gene expression. Two maize mutant lines (*gln1.3* and *gln1.4*), deficient in two genes encoding cytosolic glutamine synthetase, a key enzyme involved in nitrogen assimilation, were previously characterized by a reduction of kernel size in the *gln1.4* mutant and by a reduction of kernel number in the *gln1.3* mutant. In this work, the differences in leaf gene transcripts, proteins and metabolite accumulation in *gln1.3* and *gln1.4* mutants were studied at two key stages of plant development, in order to identify putative candidate genes, proteins and metabolic pathways contributing on one hand to the control of plant development and on the other to grain production.

**Results:**

The most interesting finding in this study is that a number of key plant processes were altered in the *gln1.3* and *gln1.4* mutants, including a number of major biological processes such as carbon metabolism and transport, cell wall metabolism, and several metabolic pathways and stress responsive and regulatory elements. We also found that the two mutants share common or specific characteristics across at least two or even three of the “omics” considered at the vegetative stage of plant development, or during the grain filling period.

**Conclusions:**

This is the first comprehensive molecular and physiological characterization of two cytosolic glutamine synthetase maize mutants using a combined transcriptomic, proteomic and metabolomic approach. We find that the integration of the three “omics” procedures is not straight forward, since developmental and mutant-specific levels of regulation seem to occur from gene expression to metabolite accumulation. However, their potential use is discussed with a view to improving our understanding of nitrogen assimilation and partitioning and its impact on grain production.

**Electronic supplementary material:**

The online version of this article (doi:10.1186/1471-2164-15-1005) contains supplementary material, which is available to authorized users.

## Background

Over the last two decades, there has been considerable interest in improving nitrogen use efficiency (NUE) in model and crop plants using quantitative genetic approaches [1], mainly because attempts to engineer plant N metabolism have on most occasions, only met with limited success [2, 3]. The aim of such studies based on the construction of genetic maps and then QTL detection, has been to identify chromosomal regions involved in the control of yield and its components and to determine the relative importance of high or low nitrogen (N) fertilisation [4, 5]. Thus, exploiting the genetic variability of grain yield and grain quality traits under N-limiting or non-limiting conditions appeared to be a key target for improving NUE. More recently, a number of quantitative genetic studies performed mostly on rice and maize, were conducted to detect QTLs for the nutritional quality of grain or stover (amino acids, protein, oil and starch content) as a function of N fertilisation [6–9].

In other quantitative studies aimed at identifying the genetic basis of NUE, in addition to agronomic traits, the association of metabolic and physiological functions with DNA markers was also investigated [10–12].

As for the agronomic traits, a significant genotypic variation was observed for various physiological traits measured in young developing leaves related to N metabolism. In particular, coincidences of QTLs for yield and its components with genes encoding cytosolic glutamine synthetase (GS1) and the corresponding enzyme activity were detected, which could partially explain the variations in yield. Since a QTL for a thousand kernel weight was coincident with a cytosolic GS (*Gln1.4*) locus, and QTLs for a thousand kernel weight and yield were coincident with another cytosolic GS (*Gln1.3*) locus [10], further work was undertaken to validate the function of these two putative candidate genes. In subsequent studies, it was also shown that the corresponding GS1.3 isoenzyme is present in the leaf mesophyll cells, whereas the GS1-4 isoenzyme is specifically localized in the leaf bundle sheath cells. In addition, it was found that in maize leaves among the five different cytosolic isoenzymes, GS1.3 and GS1.4 are the most highly expressed irrespective of the plant developmental stage [13]. Thus, the impact of the knockout mutations *gln1.3* and *gln1.4* on kernel yield and its components were examined in plants grown under non-limiting N feeding conditions [13]. The phenotypes of the two mutant lines were characterized by a reduction of kernel size in the *gln1.4* mutant and by a reduction of kernel number in the *gln1.3* mutant. Transgenic plants that overexpressed *Gln1.3* constitutively in the leaves, exhibited an increase in kernel number, thus providing further evidence that the GS1.3 isoenzyme plays a major role in controlling kernel yield under high [13] or low N fertilisation conditions [1]. The hypothesis that GS is one of the key steps in the control of cereal productivity was strengthened by a study performed on rice, in which a co-localization of a QTL for the *GS1.1* locus and a QTL for one-spikelet weight was identified [14]. As a confirmation, a strong reduction in growth rate and kernel yield was observed in rice mutants deficient in GS1.1 [15].

In order to improve our knowledge of the physiological and molecular responses of two maize mutants deficient in the expression of *Gln1.3* and *Gln1.4*, an analysis of the leaf metabolome was conducted in parallel with a proteomic and a transcriptomic study at the vegetative stage and in the middle of the grain filling period, representing key physiological and developmental stages during the life cycle of the plant. These studies provide an integrated view of the biological responses of the mutant plants, spanning from gene expression and protein content to metabolite accumulation. The possibility that these traits can be used as investigative tools to identify candidate genes for grain production, in relation to plant growth and development, is discussed.

## Results and discussion

### Changes in leaf metabolite profile of *gln1.3*and *gln1.4*mutants

In the present study, GC/MS analysis of the leaf metabolome was performed using WT, *gln1.3* and *gln1.4* mutant plants grown on a high N supply. Samples were taken at two key stages of plant development: the leaf vegetative stage (V) and 55 days after silking, referred to as leaf maturity (M), as described in Methods. At the V stage, during which young developing maize leaves efficiently assimilate CO_2_ through the C_4_ photosynthetic pathway and inorganic N for amino acid and protein synthesis [16], extensive differences were observed in metabolite accumulation between the two GS mutants in comparison to the WT (Additional file 1: Table S1).

At the V stage in the leaves of the *gln1.3* mutant, among the 48 identified metabolites that showed statistically significant differences compared to the WT in the three replicates (*P* ≤0.05), the concentrations of most of the soluble amino acids (14 out of 22), as well as a number of soluble sugars and sugar alcohols, were strongly reduced by 2 to 500 fold. The amount of the polyamine putrescine was also lower in comparison to the WT.

In the leaves of the *gln1.4* mutant, 13 metabolites were present in lower concentrations, as compared to the WT. These metabolites included various C containing molecules and some precursors of lignin biosynthesis such as coumaroylquinate, quinate, and shikimate, the content of the latter two being also lower in the *gln1.3* mutant. In addition, as determined in the *gln1.3* mutant, lower amounts of the polyamine putrescine were detected in *gln1.4,* whilst leucine was the only amino acid that exhibited a decrease. Lower amounts of carbohydrates such as galactose, sedoheptulose and sugar alcohols such as erythritol, were also detected in both *gln1.3* and *gln1.4*. All together, these results show that at the V stage and irrespective of the GS mutation, there is a decrease in the accumulation of a set of metabolites (green background in Additional file 1: Table S1).

In the leaves of the *gln1.3* mutant, a significant increase was observed in the content of a number of organic acids involved in the tricarboxylic acid (TCA) cycle, notably malate, 2-oxoglutarate, fumarate and aconitate. Such an increase was only observed in the *gln1.4* mutant, for malate alone. One possible explanation for this finding could be that in *gln1.3,* there is an accumulation of molecules that are normally used as carbon skeletons for the synthesis of amino acids. In particular these would be the amino acids derived from glutamine and glutamate that are presumably synthesized at a much lower rate when the activity of the GS1.3 isoenzyme is reduced. Interestingly, the sinapinate content of *gln1.3* was around 19 times higher compared to the WT, suggesting that part of the lignin biosynthesis pathway had been severely altered, since phenylpropanoid metabolism is extremely dependent on the N status of the plant [17]. An increase in the concentrations of raffinose, rhamnose, galactinol, mannitol and maltose has previously been observed in N-deficient maize plants [18]. The increase in inositol, a molecule related to stress conditions and involved in the control of plant growth [19], is an interesting finding, which suggests that the metabolic response caused by the lack of the GS1.3 isoenzyme is similar to that found for other abiotic stresses [20, 21]. The increase in such a range of metabolites probably occurs partly at the expense of the precursor glucose, since the increases were less in the *gln1.3* mutant, a situation similar to that also found under N limiting conditions [18]. The increase in the vitamin nicotinate, a stress protective molecule [22] and malonate, a defence compound [23], further supports the hypothesis that the partial lack of GS activity mimics stress conditions. Moreover, such stress conditions are likely to lower the demand for the respiration of alternative substrates such as 2-hydroxyglutarate [24], a molecule that accumulates almost five fold in the *gln1.3* mutant.

In the *gln1.4* mutant, the number of metabolites exhibiting an increase in their amount was much lower compared to *gln1.3*. Phenylalanine was one of the most interesting, as its accumulation probably occurred to avoid feedback inhibition of the corresponding biosynthetic pathway [25]. Such an accumulation of phenyalanine could explain why there is a decrease in the accumulation of its precursor shikimate, or its derivates coumaroylquinate and quinate, both molecules being precursors of lignin biosynthesis [26].

From the metabolite profile at the V stage, it would appear that the impact of the *gln1.3* mutation was much greater than that of the *gln1.4* mutation. Such a finding is logical, since it has been shown that the gene encoding the GS1.3 isoenzyme is constitutively expressed irrespective of the leaf developmental stage, whilst the expression of the gene encoding the GS1.4 isoenzyme is much lower and only enhanced at later stages of leaf development [16]. Moreover, it is well known that a lack of N assimilates such as amino acids before the flowering period, as observed in the metabolic profile of the *gln1.3* mutant, can cause ovule abortion [27]. Consequently, there is a reduction in the grain number of the *gln1.3* mutant [13]. At the M stage, the metabolite profiles of leaves below the ear in the two GS mutants were very different compared to the leaves of plants at the V stage. The only exceptions were metabolites such as aconitate, rhamnose and malate, which exhibited an opposite pattern of accumulation, depending on the plant developmental stage, or the GS mutation (in bold characters in Additional file 1: Table S1). Less than half of the metabolites (22 out of 48) exhibited differences in their concentration in the *gln1.3* mutant between the M and the V stage. In contrast, in the *gln1.4* mutant, the number of metabolites exhibiting an altered level of accumulation was similar between the two stages of plant development (44 for V and 52 for M). However, in the *gln1.4* mutant, the number of metabolites exhibiting an increase in concentration was three times higher at the M stage, compared to that found at the V stage (15 at M and 5 at V; Additional file 1: Table S1). As discussed above for the V stage, this could be related to the fact that the GS1.4 isoenzyme is preferentially involved in the grain filling process at the later M stage of plant development [13].

At the M stage, there was still a reduction in the amount of organic acids of the TCA cycle, but this was limited to aconitate in the *gln1.3* mutant. In the *gln1.4* mutant, the amount of malate and other organic acids derived from the TCA cycle such as citramalate and glucarate were reduced. At the grain filling stage in the *gln1.3* mutant, decreases in the amounts of several other soluble carbohydrates such as melibiose, arabinose and glycerolipids such as galactosylglycerol were observed. A considerable decrease in the amount of galactarate was detected in the *gln1.3* mutant. This decrease was much less in the *gln1.4* mutant. The biosynthesis and physiological roles of a number of the metabolites listed above have not been clearly defined and further work is required to identify their role in plants in relation to stress in general [28], and N-deficiency stress in particular. The 3-fold increase in the amount of phospho*enol*pyruvate, fructose and glucose in the *gln1.3* mutant only, indicates that during the grain filling period, a large proportion of the C molecules fuelling the TCA cycle were not used or were exported. This presumably results from an alteration in the sink capacity of the plant characterised by the considerable reduction in grain number [13].

In the *gln1.4* mutant, an increase in the concentration of coumarate and caffeate, two precursors of lignin that were not detected at the V stage, suggests that an alteration in cell wall synthesis or composition, also occurred at the M stage. Interestingly, in the *gln1.3* mutant, the decrease in the amino acids observed at the V stage was not detected at the M stage. In contrast, the *gln1.4* mutant, in which the amino acid content was not modified at the V stage, exhibited a 2 to 3 fold increase in valine, serine, threonine, methionine, aspartate, alanine and glutamate at the M stage. Since we showed previously that the synthesis of a number of these amino acids still occurred at the M stage in WT plants [18], it is likely that their accumulation (glutamate in particular) is due to the lack of GS1.4 activity during the grain filling period. As these amino acids are not exported to the grain until this M stage of plant development, there is a reduction in grain size in the *gln1.4* mutant [13], as N translocation is required to fill the grain [29, 30]. However, it cannot be completely ruled out that the amino acids could be exported to the grain at a later stage of development.

### Changes in the leaf proteome of the *gln1.3*and *gln1.4*mutants

At the V stage, when the leaf proteome of the WT was compared with the *gln1.3* and *gln1.4* mutants, 46 protein spots were identified as exhibiting significant differences in their volume (p < 0.05) in the former and 14 proteins in the latter. The densitometric quantification of these differences is presented in Additional file 1: Tables S2 and S4. At the M stage, the volumes of 51 protein spots were significantly modified in the *gln1.3* mutant, whereas only 28 proteins were modified in the *gln1.4* mutant (Additional file 1: Tables S3 and S5).

At the V stage, among the 46 identified proteins in the *gln1.3* mutant, 29 were present at lower and 17 at higher concentrations. These proteins are involved in a variety of physiological processes including C assimilation, photosynthesis, cell wall metabolism, proteolysis, stress/defence mechanisms and translation. In the *gln1.4* mutant the same biological processes were altered, however compared to the *gln1.3* mutant, there were much fewer proteins present in lower (10 proteins) or higher amounts (4 proteins). Therefore, it can be concluded that at the protein accumulation level, the impact of the *gln1.3* mutation was much stronger than that of *gln1.4*.

One of the most interesting results from the proteomic study performed at the V stage, was the stronger impact of the *gln1.3* mutation compared to the *gln1.4* mutation on the accumulation of proteins involved in C assimilation and C metabolism, whereas those involved in N metabolism displayed no changes in their level of accumulation. It was also observed that among the 29 proteins present in lower amounts in the *gln1.3* mutant, 8 of the same proteins were also found to be present in lower amounts in the *gln1.4* mutant (Table 1). Such findings suggest that at the proteomic level the two GS mutations had a similar impact on a number of biological processes. In particular, the decrease in amount of the enzymes ribose-5-phosphate isomerase [31] and NADP-dependent malate dehydogenase [32] suggests that the pentose phosphate pathway and the C_4_ photosynthetic pathway respectively, were down-regulated in response to the lower flux of reduced N going through the reaction catalysed by GS1.3 or GS1.4. In addition, the finding that peroxiredoxin and glutaredoxin (CAXIP1 also called GrxS14) were less abundant in the two GS mutants indicates that redox signals [33] and oxidative stress responses [34], in relation to photosynthesis, were also altered. In line with the finding that C metabolism and some of its associated processes were modified in the two GS mutants, an accumulation of the translation initiation factor IF3, a protein that is an important component of the regulation of photosynthetic gene expression [35], was also detected.

**Table 1:** **Proteins detected in lower and higher amounts in the** ***gln1.3*** **and** ***gln1.4*** **mutants as compared to the wild type**

Identification number	Function/pathway	Lower amount at the V stage
TC463245	Ammonia assimilation	Glutamine synthetase root isozyme 3 (GS1-3) {Zm} P38561
TC467371	C_4_ carbon metabolism	Malate dehydrogenase [NADP], chloroplast precursor E {Zm} P15719
TC294877	Oxidative stress	CAXIP1-like protein {At} AY157989
TC288041	Pentose phosphate	Ribose-5-phosphate isomerase precursor {So} Q8RU73
TC287751	Proteolysis	ATP-dependent protease {Os} Q50LH5
TC524782	Redox signalling	Peroxiredoxin-2E-2, chloroplast precursor {Os} Q69TY4
TC287421	Translation	60S acidic ribosomal protein P2B {Zm} O24415
TC310278	Translation	Eukaryotic translation initiation factor 5A {Zm} P80639
		**Higher amount at the V stage**
TC499661	Translation	^a^Translation initiation factor IF-3-like Q6K674
		**Lower amount at the M stage**
TC279798	Abiotic stress	DnaK-type molecular chaperone hsp70 {Os} Q53NM9
TC312072	Chloroplast structure	Plastid-lipid-associated protein 3 {Os} Q7XBW5
TC279235	Development	Actin 1 {0 s} A2XLF2
TC304992	Glycolysis	Fructose-bisphosphate {Os} aldolase Q69V57
TC286949	Organic acid synthesis	NADP-specific isocitrate dehydrogenase {Os} Q9XGU8
TC283000	Oxidative stress	Peroxidase J {Os} Q7F1U1
TC281878	Pathogen defence	Hairpin binding protein 1 {Zm} Q5QJA2
TC293364	Pentose phosphate	^a^Phosphoribulokinase {Tae} P26302
TC305088	Pentose phosphate	Transketolase {Zm} Q7SIC9
TC293364	Pentose phosphate	^a^Phosphoribulokinase {Tae} P26302
TC292245	Photosynthesis/Calvin cycle	^b^Glyceraldehyde-3-phosphate dehydrogenase A {Zm} P09315 (*gln1.4*)
TC303207	Polyamine biosynthesis	Arginine decarboxylase {Os} Q01HV9
TC304817	Redox signalling	2-Cys peroxiredoxin BAS1 {OS} Q6ER94
TC312398	Signalling	Tyrosine phosphatase {Os} Q9LKK3
TC311663	Starch synthesis	Glucose-1-phosphate adenylyltransferase {Zm} A5GZ73
TC307030	Stress/Defence	OSJNBa0006A01.5 protein {Os} Q7F9L9
TC310158	Translation	Elongation factor 1-delta 1 {Os} Q40680
		**Higher amount at the M stage**
TC310669	Photosynthesis/C/N/Metabolism	Ferredoxin {Zm} Q9SLP5
TC292245	Photosynthesis/Calvin cycle	^b^Glyceraldehyde-3-phosphate dehydrogenase A {Zm} P09315 (*gln1.3*)
TC293513	Stress/Defence	Chaperonin CPN60-2, mitochondrial precursor {Zm} Q43298

^a^Isoforms of the same protein exhibiting a different level of accumulation.

^b^Protein showing an opposite pattern of accumulation in *gln1.3* and *gln1.4.*

At the M stage, the differences in protein profiles of the two GS mutants were not the same as those detected at the V stage. Although in *gln1.3,* three proteins were found to be present either in higher or lower amounts at both stages of plant development (protein names in italics in Additional file 1: Tables S2 and S3). In the *gln1.3* mutant and to a lesser extent in the *gln1.4* mutant, the changes observed in the protein profiles at the M stage indicate that the main metabolic functions involved were related to photosynthesis and to C metabolism. Proteins involved in the efficiency of the photosystems and the glycolytic, pentose phosphate and starch biosynthesis pathways were present in much lower quantities. Interestingly a significant number of these proteins such as phosphoribulokinase, and NADP-specific isocitrate dehydrogenase (Table 1), exhibited a common pattern of accumulation in the two GS mutants. Such findings, in line with the accumulation of metabolites, suggest that, during the grain-filling period, the lower flux of N going through the reaction catalysed by GS triggers a reduction in C assimilation, likely because the sink capacity of the plant in terms of C translocation to the developing ear is strongly reduced. Surprisingly, lower amounts of the enzyme glyceraldehyde-3-phosphate dehydrogenase were detected in the *gln1.4* mutant, whereas in *gln1.3* there was an accumulation of the protein. Such contradictory patterns of accumulation could be related to the stress-responsive nature of the enzyme [36], which appears to be specific for each *gln1.3* and *gln1.4* mutation.

In addition, among the proteins exhibiting a decrease in their amount in the two GS mutants, there were two isoforms of the enzyme arginine decarboxylase. This result suggests that polyamine biosynthesis was modified in *gln1.3* and *gln1.4* presumably in response to the stress caused by the reduction in leaf GS activity. The involvement of polyamines during abiotic stress [37] is in line with the finding that the putrescine and spermidine contents were decreased or increased respectively in the GS mutants (see Additional file 1: Table S1). The presence of higher or lower amounts of a number of oxidative stress-responsive proteins, such as peroxidases, superoxide dismutase, peroxiredoxins as well as various biotic and abiotic stress-responsive proteins, was evident either in both, or only one of the two GS mutants (Table 1 and Additional file 1: Tables S3 and S4).

At the M and V stages, the common set of proteins that were down-regulated and up-regulated in *gln1.3* and *gln1.4* were different (Table 1). This result suggests that in the two GS mutants there is a core set of proteins corresponding to different biological processes that are specifically altered at each of the two developmental stages examined. As in a number of proteomic studies related to plant stress (see [38] for a review), relatively few proteins exhibited significant differences in their level of accumulation in the two GS mutants. Reduced GS activity altered the accumulation of proteins and enzymes involved in photosynthesis, primary C metabolism and stress-related regulatory mechanisms, a plant response similar to that observed under N deficient conditions [39–41, 18].

### Correlations between the two *gln1*mutations, mRNA accumulation and a variety of metabolic, signalling and developmental processes

At the V stage, 106 and 52 mRNA transcripts were present in lower amounts in the leaves of the *gln1.3* and the *gln1.4* mutants respectively, when compared to the WT. (Additional file 1: Tables S6a and S8a). The biological processes most significantly reduced in the two *gln1* mutants, were C metabolism, stress/defence, proteolysis, signalling, transport, as well as components of the translation and transcription machinery. In the *gln1.3* mutant, the reduction in transcript accumulation for ADP-glucose pyrophosphorylase, NAD-malate dehydrogenase, ribulose-5-phosphate 3-epimerase and pyruvate dehydrogenase suggests that, at least at the transcriptional level, important steps of primary C assimilation were down-regulated (Additional file 1: Table S6a). The high level of pyruvate dehydrogenase kinase mRNA accumulation further supports this hypothesis, as phosphorylation of the pyruvate dehydrogenase complex would reduce enzyme activity and consequently the C flux through the TCA cycle [42]. Another important impact of the *gln1.3* mutation was a decrease in the amount of transcripts encoding proteins involved in sugar and amino acid transport, which suggests that the translocation and thus the subsequent accumulation of a number of C and N metabolites may be reduced.

The decrease both in terms of the number of biological processes involved and the portfolio of genes belonging to each of these processes was much higher in the *gln1.3* mutant compared to the *gln1.4* mutant. Such findings, in agreement with the metabolomic and proteomic studies, could explain why the impact of the *gln1.3* mutation, compared to the *gln1.4* mutation, was much higher in terms of grain yield penalty [13].

At the V stage, transcripts of 139 genes were present in higher amounts in the *gln1.3* mutant as compared to the WT (Additional file 1: Table S6b), whereas in the *gln1.4* mutant there were only 54 (Additional file 1: Table S8b). Transcripts of various genes encoding proteins having different metabolic and signalling roles, in stress/defence, translation, transcription and transport were predominantly represented. Whilst those genes encoding enzymes involved in C and secondary metabolite metabolism and precursors of cell wall synthesis were present to a lesser extent. Transcripts of asparaginase, which exhibited a strong homology with asparaginase 2 from grasses, were more than three-fold higher in *gln1.3* than in the WT, otherwise there were no major changes in gene expression of the proteins involved in N assimilation. Again, the impact of the GS mutation with respect to the enhanced expression of a number of genes involved in the various functions listed in Additional file 1: Tables S5b and S7b, was much higher in the *gln1.3* mutant.

A similar pattern of transcript accumulation was observed at the M stage, both in the *gln1.3* and *gln1.4* mutant. The number of up- and down-regulated genes in the *gln1.3* mutant was 133 and 170 respectively, which is quantitatively similar to that detected at the V stage. In *gln1.4,* there was an increase in the expression of 102 genes and a decrease in the expression of 96 genes, indicating that the number of genes with a modified level of expression was approximately two-fold higher compared to the V stage (Additional file 1: Tables S7a, S7b, S9a and S9b). As identified at the V stage, the main biological processes that were altered in the *gln1.4* mutant were metabolism, signalling, stress/defence and transport. However, at the M stage in comparison to the V stage, the number of up- or down-regulated genes encoding enzymes involved in C metabolism was much lower. Interestingly, the number of genes encoding proteins relating to proteolytic activity were significantly increased in the *gln1.4* mutant at the M stage compared to the V stage (Additional file 1: Table S9b). The hypothesis that the GS1.4 isoenzyme is involved in the recycling of ammonia released during protein degradation [13], may explain the changes observed in the expression of genes encoding various proteases. In line with this observation, it has also been found that protein degradation was enhanced in transgenic plants overexpressing cytosolic GS [43]. As identified at the V stage, the impact of the two GS mutations on genes encoding enzymes and proteins involved in N assimilation and recycling remained very limited. Such observations lead to the conclusion that in maize, cytoplasmic GS is a central hub at the cross roads of a number of plant metabolic and regulatory functions, other than N assimilation *per se.*

In recent “omics”-based studies performed on maize and other species [44, 45, 18], it has been shown that the plant developmental stage must be taken into account, as there are large differences both at the physiological and molecular levels during the transition from N assimilation to N remobilisation [18, 38]. When maize plants were grown under N-limiting conditions, no decreases or increases in the amounts of transcripts were found that were common to the V and M stage [18]. In contrast, in the two GS mutants, a number of transcripts (70 in *gln1.3* and 29 in *gln1.4*) exhibited a similar pattern of accumulation at both stages of plant development (Additional file 1: Tables S10 and S11). A qRT-PCR experiment performed on four selected genes exhibiting an increase or a decrease in the *gln1.3* and *gln1.4* mutants in comparison to the WT at the V and the M stage of plant development, confirmed that the level of leaf transcript accumulation was similar to that observed in the 46 K microarray experiment (Additional file 1: Figure S1).

A list of selected genes that exhibited a fold change higher than 10 in a mutant compared to the WT is shown in Tables 2 and 3. They could be key candidate genes representative of the reprogramming machinery involved in the control of shoot growth and development in the two mutants, in which only grain yield, but not leaf size or leaf number, was affected [13]. Our study also shows that for a number of key metabolic pathways such as the primary assimilation of C and N, major changes could occur in terms of metabolite accumulation as shown in the present study (see Additional file 1: Table S1), but not at the gene expression level. As previously shown in several investigations, very little direct correspondence between the accumulation of metabolites, proteins and mRNA transcripts has been found in “omics”-based studies [18, 46]. Such a situation is probably due to the complex nature of biological networks and to the fact that both tissue and cellular compartmentation and translocation between different organs, are generally not taken into account (see [47] for a review).

**Table 2:** **Transcripts exhibiting a large increase or decrease in the** ***gln1.3*** **and** ***gln1.4*** **mutants at the V stage as compared to the wild type**

Identification number	Functional category	Putative annotation	FC/WT
***gln1.3*** **mutant**			
TC255750	Transcription	MLIP 15 {Zm} Q41833	430.37
TC260113	Photosynthesis	23 kDa polypeptide of photosystem II {Nt} Q04126	283.37
TC253688	Transposon	Transposase {Zm} Q5UDR1	43.62
TC261195	Proteolysis	26S proteasome regulatory subunit-like protein {Os} Q69Q88	34.37
TC250399	Signalling	Pyruvate dehydrogenase kinase isoform 2 {Zm} O82424	30.98
TC278448	C metabolism	Phosphoribulokinase precursor {Os} P26302	29.41
TC251968	Secondary metabolism	Chalcone isomerase-like {Os} Q6EQW2	28.12
TC270988	Signalling	Protein phosphatase type-2C {Zm} Q9FQY2	23.61
TC253650	Transport	Putative nitrite transporter {Os} Q5Z6P7	22.38
TC276810	Signalling	Putative calcium binding protein {Os} Q652U8	22.14
TC271736	Translation	Ribosomal protein S21-like protein {Os} Q6YUV4	18.77
AZM4_72986	Nuclesome assembly	Histone H2B.2 {Zm} P30756	17.14
TC264476	Transcription	MA3 domain-containing protein {Os} Q10PT6	14.19
TC249105	Metabolism	Ferredoxin 1 {Zm} P27787	13.43
TC279125	Cell wall	Hydroxyproline-rich glycoprotein-like protein {Os} Q67UA8	12.36
TC264087	Stress/Defence	AvrRpt2-induced protein 2-like {Os} Q6Z2W4	11.97
TC277283	Cell wall	Glycoside hydrolase family 28 protein {Os} Q10B12	11.37
TC252426	Lipid metabolism	3-hydroxybutyryl-CoA dehydrogenase-like protein {At} Q9LDF5	11.03
TC213118	Metabolism	Sesquiterpene cyclase {Zm} Q9FEF5	10.69
TC270254	Metabolism	Putative mitochondrial F0 ATP synthase D chain {Os} Q9FT52	10.54
BM382459	C metabolism	NAD-malate dehydrogenase precursor {Nt} Q9XQP4	0.07
AZM4_41292	C metabolism	ADP-glucose pyrophosphorylase small subunit {Zm} Q947C0	0.05
TC192492	Signalling	Peptidyl-prolyl *cis-trans* isomerase {Os} A2WYA7	0.04
TC256186	Transport	Putative integral membrane protein {Os} Q688W0	0.02
***gln1.4*** **mutant**			
TC260113	Photosynthesis	23-kDa polypeptide photosystem II {Nt} Q04126	200.22
TC266788	Metabolism	Putative NADPH HC toxin reductase {Os} Q7X6N6	107.17
TC199171	Proteolysis	Putative uncharacterized protein {Os} Q75K52	67.99
TC259563	Transport	Ubiquinol-cytochrome C reductase complex ubiquinone-binding protein {Os} Q9LDS7	65.76
TC272126	Translation	Putative uncharacterized protein {Ps} A9NTN5	60.23
BM380216	Cell wall	Glycine-rich cell wall structural protein precursor {At} P27483	36.64
TC266666	Metabolism	NAD dependent epimerase/dehydratase family protein {Os} Q10CW5	24.91
TC195262	Stress/Defence	Isoleucyl-tRNA synthetase-like {Os} Q67WM1	23.25
TC270988	Signalling	Protein phosphatase type-2C {Zm} Q9FQY2	21.34
CD990526	Stress/Defence	Lipoate biosynthesis-LIP5 {Os} O48673	15.74
TC200462	Unknown	Putative uncharacterized protein {Os} Q75IJ0	13.59
TC260627	Unknown	Putative uncharacterized protein {Sb} Q8LKT8	13.00
TC259358	Signalling	Type 2 non specific lipid transfer protein precursor {Tae} Q2PCC5	12.57
TC262845	Signalling	MAP kinase 4 {Zm} Q9ZWJ6	11.55
AZM4_8141	Transport	Putative lipid transfer protein {Os} AZM4_8141	0.09
TC262118	C Metabolism	Putative aldose reductase {Os} Q0DHM8	0.09
TC250487	C metabolism	Putative trehalose-6-phosphate synthase {At} Q9C9W6	0.08
TC265311	Stress/Defence	DnaJ protein homolog-like {Os} Q6YT03	0.07
TC217530	Transcription	Homeodomain leucine zipper protein 16 {Os} Q6Q502	0.06
BM380114	Transposon	Transition Protein-TNP2 {Os} Q948C7	0.03
TC198868	Stress/Defence	Putative interferon-related protein {Os} Q6ZIP6	0.02
CF637160	Translation	60S acidic ribosomal protein P3 {Zm} O24413	0.01
TC197759	Stress/Defence	rRNA N-glycosidase {Zm} Q41851	0.01

FC/WT corresponds to the fold change in the mutants compared to wild type (WT) leaves for transcripts exhibiting significant variations (Student t-test, *P* ≤0.05) in their amount.

**Table 3:** **Transcripts exhibiting a large increase or decrease in** ***gln1.3*** **mutant at the M stage as compared to the wild type**

Identification number	Functional category	Putative annotation	FC/WT
TC250399	Signalling	Pyruvate dehydrogenase kinase isoform 2 {Zm} O82424	157.42
AI065741	Stress/Defence	Thaumatin-like cytokinin-binding protein {Os} Q2QND8	47.43
TC251968	Secondary metabolism	Chalcone isomerase-like {Os} Q6EQW2	44.04
TC264087	Stress/Defence	AvrRpt2-induced protein 2-like {Os} Q6Z2W4	35.66
TC260211	Lipid metabolism	^a^Allene oxide cyclase {Zm} Q6RW09	33.17
TC271736	Translation	Ribosomal protein S21-like protein {Os} Q6YUV4	32.63
TC253688	Transposition	Transposase {Zm} Q5UDR1	29.82
TC279125	Cell wall	Hydroxyproline-rich glycoprotein-like protein {Os} Q67UA8	29.29
AZM4_72986	Nucelosome assembly	Histone H2B.2 {Zm} P30756	26.47
AI065444	Translation	40S ribosomal protein S26 {Os} P49216	23.65
TC277283	Cell wall	Glycoside hydrolase family 28 protein {Os} Q10B12	23.09
TC252413	Metabolism	Cinnamyl alcohol dehydrogenase {Lp} Q8S411	22.61
TC270142	Transcription	Auxin response factor 1 {Os} Q2QQX6-2	18.62
TC259350	Cell wall	Exopolygalacturonase precursor {Zm} P35339	17.51
TC277223	Transport	Putative monosaccharide transporter 1 {Os} Q69S90	16.03
CF056912	Transcription	Histone deacetylase {Zm} Q9ZTP8	14.72
TC276810	Signalling	Putative calcium binding protein {Os} Q652U8	13.63
TC200056	Secondary metabolism	4-coumarate--CoA ligase 4CL1 {Lp} Q9M7S3	12.58
TC251967	Secondary metabolism	Chalcone isomerase-like {Os} Q6EQW2	12.41
TC192449	Cell wall	Pectate lyase {Zm} Q43861	12.36
TC271645	Transport	Chloroplast glucose-6-phosphate/phosphate translocator {Ps} A4UTS2	11.94
TC270824	Stress/Defence	Putative shock protein SRC2 {Os} Q0JMR3	11.76
AW289103	Cell wall	Exopolygalacturonase precursor {Zm} P26216	11.61
TC264439	Signalling	Zinc finger protein family-like {Os} Q6ZKY1	10.83
TC271833	C metabolism	Granule bound starch synthase IIa {Zm} A4URH2	10.80
AZM4_101226	Metabolism	Ferredoxin {Zm} AZM4_101226	10.19
TC249219	Proteolysis	26S proteasome regulatory particle non-ATPase subunit3 {Os} Q8W3N4	10.12
TC269702	C metabolism	Arabinogalactan protein {Nt} Q9FUL8	10.03
CF633665	Metabolism	Glycosyltransferase {Sb} Q5QPY7	0.09
AI979477	Transport	Ammonium transporter 1–3 {Os} Q947M9	0.09
TC273598	C metabolism	Beta-glucosidase 31 {Os} B7F7K7	0.08
TC273931	Transport	Putative peptide transport protein {Os} Q7XDJ1	0.08
TC207366	Signalling	Putative calcium-dependent protein kinase {Os} Q6I587	0.08
TC254765	Stress/Defence	Glutathione S-transferase GST 34 {Zm} Q9FQA5	0.07
CF029576	Transcription	SCARECROW gene regulator, putative related cluster {Os} Q53MB4	0.07
TC255650	Metabolism	Putative trehalose-6-phosphate synthase {Os} Q6Z548	0.06
TC264842	Stress/Defence	101 kDa heat shock protein {Tae} Q334I0	0.05
BG835945	C metabolism	Putative beta-1,3-glucanase {Os} Q6Z9Y9	0.04
TC266129	Transcription	Putative transcription activator RF2a {Os} Q10LT0	0.04
BG458838	Unknown	Expressed protein {Os} Q6AU54	0.03
BM501271	C metabolism	Putative beta-1 3-glucanase {Os} Q6Z9Y9	0.03
TC266256	Metabolism	S-adenosylmethionine decarboxylase {Sa} A4GXE8	0.02
TC271423	Stress/Defence	Glutathione S-transferase GST 18 {Zm} Q9FQC1	0.01
AZM4_124731	Metabolism	Putative P450 monooxygenase {Os} BAD09944.1	0.01

FC/WT corresponds to the fold change in the mutants compared to wild type (WT) leaves for transcripts exhibiting significant variations (Student t-test, *P* ≤0.05) in their amount.

### Common changes in key biological functions are found in the *gln1.3*and *gln1.4*mutants that are dependent on the plant developmental stage

A limited number of transcripts exhibited a similar pattern of accumulation in both the *gln1.3* and *gln1.4* mutants, suggesting that the decrease in leaf GS1.3 and GS1.4 activity induces a common plant response at the transcriptional level. At the V stage, in both mutants, 11 genes were up-regulated, 2 genes were down-regulated and 2 genes showed an opposite pattern of expression. (Additional file 1: Table S12). At the M stage, 19 genes were up-regulated, 19 genes were down-regulated and 2 genes showed an opposite pattern of expression (Additional file 1: Table S13). Interestingly in both mutants, transcripts for a 40S and 60S ribosomal protein showed an opposite pattern of accumulation at the V stage and the M stage respectively.

In a similar manner to the proteomic changes, the responses in transcript accumulation could provide clues as to how the plant maintains normal vegetative growth when sink organs such as the grain do not develop and thereby import less C and N assimilates. At the V stage in the two GS mutants, the massive accumulation (more than 400 fold compared to the WT) of Muscular LMNA-Interacting Protein (MLIP) 15 mRNAs encoding a Leucine Zipper Transcription Factor (bZIP) involved in abiotic stress tolerance [48], suggests that this protein is a major stress-responsive element induced in response to the lack of cytosolic GS activity. A 23 kDa photosystem II protein was also highly up-regulated with more than a 200-fold increase in mRNA accumulation in the two mutants. When GS activity is reduced, the photosynthetic products are not used for grain filling and it is then possible that there is excess light, which exceeds the requirement of the plant to assimilate CO_2_. Consequently, the light energy needs to be dissipated as thermal energy through an increase in the synthesis and repair of the pigment antenna of photosystem II [49, 50].

The accumulation of transcripts for isoleucyl-tRNA synthetase in the two GS mutants is intriguing. Although the role of this enzyme is well established in lower organisms, the information on its role in higher plants is scarce. Based on the results obtained in microorganisms [51, 52], it is attractive to think that there are some important post-transcriptional modifications resulting from the lack of the GS1.3 and GS1.4 isoenzyme, which could modify gene codon recognition and thus the protein sequence. The increase in the steady state level of mRNA encoding a putative mannitol transporter is also an interesting result. It is well established that such a monosaccharide transporter is involved in a variety of biological processes related to phloem function, resource allocation, plant defence and sugar signalling [53].

Compared to the V stage, there was a clear balance between the up- and down-regulated genes at the M stage. Among the most strongly up-regulated genes (more than 60-fold), one encoding a cytochrome P450 monoxygenase CYP72A27 (which can be subjected to transposition and then act as a retrotransposon [54]) was particularly interesting. Moreover, this type of enzyme can be involved in the synthesis of secondary metabolites that could be used as precursors of compounds such as lignin [55]. The changes in the accumulation of secondary metabolites observed in a previous study [56] and in the present investigation (see Table 4 and Additional file 1: Table S1), also indicate that lignin biosynthesis was altered, thus possibly affecting shoot structure and ear development [57]. In agreement with this hypothesis, a gene encoding a putative cinnamoyl-CoA reductase, the first enzyme after the branch of the lignin biosynthetic pathway [58], was also up-regulated. The strong accumulation of transcripts for a glycoside hydrolase, an enzyme involved in cell wall polysaccharide metabolism [59] and the considerable decrease in the amount of mRNA for an acid chitinase [60], further support the hypothesis that there was some readjustment in the production of a number of structural compounds during plant growth and development. The increase in the amount of transcripts encoding an allene oxide cyclase is more difficult to interpret, but it is possible that an abiotic stress response mediated by jasmonic acid [61] is induced when the GS1.3 and GS1.4 isoenzymes are not active. In addition to the number of metabolic and regulatory roles altered in the two GS mutants, an important reprogramming of translational and post-translational activity is likely to occur at the same time, since a number of transcripts encoding ribosomal proteins and a RNA recognition motif protein [62] were present in larger amounts in both the *gln1.3* and *gln1.4* mutants. It is also worth noting that a number of genes of unknown function exhibited an altered level of expression.

**Table 4:** **Overview of the main changes occurring in a leaf of the** ***gln1.3*** **and** ***gln1.4*** **mutants at the metabolome, proteome and transcriptome level**

TRANSCRIPTOME	PROTEOME	METABOLOME	TRANSCRIPTOME	PROTEOME	METABOLOME
**VEGETATIVE (V)**					
	**Lower in** ***gln1.3*** **-**			**Higher in** ***gln1.3***	
**C METABOLISM** (10)	C Metabolism (4)	**C METABOLISM** (12)	C metabolism (4)	**C metabolism** (7)	**C METABOLISM** (14)
N metabolism (1)		**N METABOLISM** (15)	N metabolism (1)		
CW Metabolism (3)	CW Metabolism (3)	CW Metabolism (2)	CW Metabolism (3)	CW Metabolism (1)	CW Metabolism (1)
**METABOLISM** (20)	**Metabolism** (5)	Metabolism (1)	**METABOLISM** (22)	Metabolism (2)	Metabolism (2)
**Proteolysis** (7)	Proteolysis (3)		**Proteolysis** (7)		
**SIGNALLING** (16)			**SIGNALLING** (19)		
**STRESS/DEFENCE** (10)	**Stress /Defence** (8)		**STRESS/DEFENCE** (25)	Stress/Defence (2)	
**TRANSPORT** (14)			**TRANSPORT** (12)		
**Transcription** (8)			**TRANSCRIPTION** (10)		
Translation (4)	Translation (3)		**Translation** (8)	Translation (2)	
	**Lower in** ***gln1.4***			**Higher in** ***gln1.4***	
C metabolism (2)	C Metabolism (2)	**C METABOLISM** (8)	C metabolism (1)	C Metabolism (1)	**C METABOLISM** (3)
	N metabolism (1)	N Metabolism (1)			N Metabolism (1)
		CW (3)			
Metabolism (4)			Metabolism (4)		
Proteolysis (1)	Proteolysis (1)		Proteolysis (3)		
**Signalling** (7)			**Signalling** (7)		
**Stress/Defence** (8)	Stress/Defence (2)		**Stress/Defence** (7)	Stress/Defence (1)	
Transport (3)			**Transport** (6)		
Translation (4)	Translation (2)		Translation (3)	Translation (2)	
**MATURITY (M)**					
	**Lower in** ***gln1.3***			**Higher in** ***gln1.3-***	
**C Metabolism** (6)	**C METABOLISM** (9)	**C METABOLISM** (12)	**C METABOLISM** (10)	C Metabolism (5)	**C METABOLISM** (5)
N Metabolism (3)	N metabolism (2)		N Metabolism (1)	N Metabolism (1)	
CW Metabolism (1)			**CW METABOLISM** (22)	CW Metabolism (1)	CW Metabolism (1)
**METABOLISM** (21)	Metabolism (2)		**METABOLISM** (15)	Metabolism (5)	Metabolism (3)
**PROTEOLYSIS** (13)	Proteolysis (2)		**Proteolysis** (6)		
**SIGNALLING** (14)	Signalling (1)		**SIGNALLING** (20)		
**STRESS/DEFENCE** (22)	**Stress/Defence** (7)		**STRESS/DEFENCE** (24)	Stress/Defence (2)	
**TRANSPORT** (15)			**TRANSPORT** (16)	Transport (1)	
**Transcription** (7)			**TRANSCRIPTION** (10)		
Translation (3)	Translation (2)		**Translation** (8)	Translation (4)	
	**Lower in** ***gln1.4***			**Higher in** ***gln1.4***	
**C Metabolism** (6)	**C Metabolism** (8)	C Metabolism (5)	**C Metabolism** (5)		C Metabolism (3)
CW Metabolism (1)		CW Metabolism (1)	CW Metabolism (1)		CW Metabolism (2)
N Metabolism (4)	N metabolism (2)	N Metabolism (2)	N Metabolism (2)		**N Metabolism** (7)
**METABOLISM** (13)		Metabolism (3)	**METABOLISM (**18)	Metabolism (1)	Metabolism (3)
**Proteolysis** (7)			Proteolysis (4)		
**SIGNALLING** (12)	Signalling (1)		**SIGNALLING** (13)		
**STRESS/DEFENCE** (12)	**Stress/Defence** (7)		**STRESS/DEFENCE** (11)	Stress/Defence (4)	
**Transport** (9)			**TRANSPORT** (10)		
**Transcription** (8)			**Transcription** (8)		
**Translation** (6)	Translation (1)		**Translation** (7)		

*gln1.3* and *gln1.4* mutant plants were grown under high N fertilisation conditions and harvested at the vegetative (V) or maturity (M) stage. The functional category that exhibited the greatest alterations when considering the number of different metabolites, proteins and mRNA transcripts, is indicated in bold capital letters (total number ≥10). Those in bold lower case had an intermediate level of increase or decrease (between 5 and 10). The others in lower case not in bold were ≤5. The numbers in brackets indicate the number of representatives in each category. The different functional categories were obtained from the different analyses presented in Additional file 1: Tables S1 to S9. C = carbon; N = nitrogen and CW = cell wall.

### Integrated view of the differences in accumulation of metabolites, proteins and transcripts

**Figure 1:**
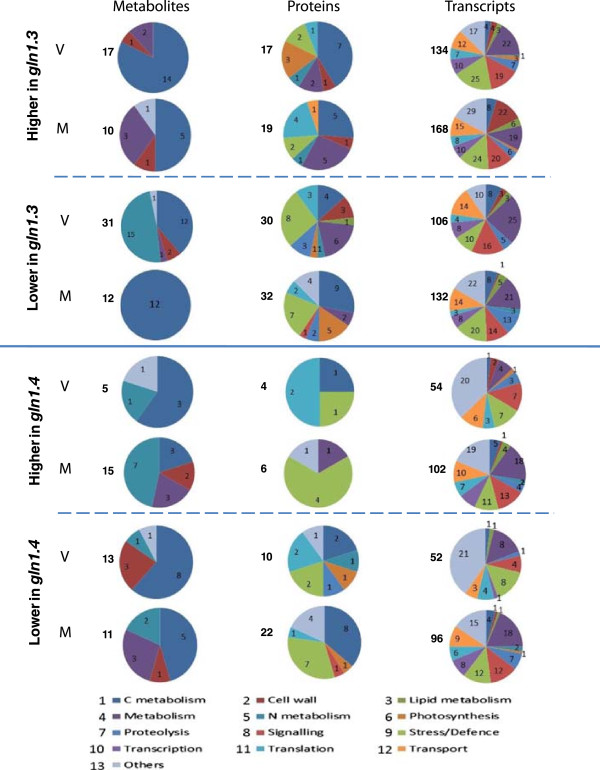
**Functional categories of metabolites, proteins and gene transcripts isolated from the leaves of maize** ***gln1.3*** **and** ***gln1.4*** **mutant plants, exhibiting differences in their level of accumulation.** Pie charts show the number of metabolites, proteins and transcripts for each functional class identified in the three “omics” experiments exhibiting an increase or a decrease in the leaves of the *gln1.3* and *gln1.4* mutants at the vegetative (V) and mature (M) stage of leaf development. For each mutant and each developmental stage, the total number of changes is indicated on the left side of the pie chart.

As in a previous study aimed at integrating the three different “omics” investigations when maize plants were grown under N-limiting conditions [18], we have made a similar attempt to integrate the transcriptome, proteome and metabolome datasets and the impact of the two *gln1* mutations at two key stages of plant development. Such an integrated “omics” picture can provide important information on the impact of the two *gln1* mutations on the physiological adaptation of the plant. Irrespective of their biological functions, the quantitative differences observed in mRNA transcript, protein and metabolite accumulation in relation to the plant developmental stage are presented in Table 4. In addition, the relative importance of these processes in terms of number of metabolites, proteins and transcripts exhibiting differences within common functional categories across the three “omic” studies is presented in Figure 1. The most important differences were generally observed at the transcript accumulation level, regardless of the plant developmental stage.

Fewer variations in functional diversity were observed at the metabolome level, although changes in C and N metabolites and to a lesser extent secondary metabolites, precursors of cell wall synthesis, were the main consequences of the *gln1.3* and *gln1.4* mutations (see also Additional file 1: Table S1). Nevertheless, it is clear that the process that is the most strongly affected in the two *gln1* mutants is C metabolism, irrespective of the mutation and of the plant developmental age. Important changes in the accumulation of leaf C metabolites, mostly represented by soluble sugars, sugar alcohols and organic acids were the main consequences of N deficiency [18], a metabolic signature that is similar to that described here in the two GS mutants (Table 4). Such a finding is consistent with the now established hypothesis that the reaction catalysed by the enzyme GS is the major route facilitating the incorporation of inorganic N into organic molecules [63, 64].

Interestingly, the *gln1.3* mutation had only a limited impact on N metabolism across the three “omics” components, both at the V and the M plant developmental stage. In the *gln1.4* mutant, simultaneous changes in metabolites, proteins and transcript accumulation related to N assimilation and recycling were only observed at the M stage. This observation is consistent with the finding that the bundle sheath-specific GS1.4 isoform is encoded by a gene induced during leaf aging, which plays a catabolic role in the reassimilation of ammonium released during protein degradation in senescing leaves at the M stage [13].

In a similar manner to the N-deficient growth conditions investigated by [18], in at least two of the three “omics” experiments, the synthesis of cell wall components, as well as stress and defence mechanisms were altered in the two GS mutants. These alterations include the accumulation of marker metabolites for stress conditions, which agrees with the observation that C metabolism, lignin biosynthesis and stress-responsive elements are co-ordinately regulated [65]. Although throughout the plant life cycle, both at the transcriptome and proteome level, a number of stress-responsive elements and plant defence mechanisms specific for the plant developmental stage were up- or down-regulated in the two GS mutants (Table 2), in a similar manner to when N is limiting [18].

Differences in the protein profile of the two GS mutants in comparison to the WT were relatively minor, again comparable with the impact of N limitation on the leaf proteome [18]. Moreover these proteins were involved in a limited number of plant biological functions. One explanation is that some of the low-abundance structural, or regulatory proteins cannot be detected using 2-D gel electrophoresis in comparison to a maize pan-transcriptome. It is also possible that the decrease in GS1 activity, may involve a number of post-translational protein modifications that were not investigated in the present study. It is likely that such post-translational modifications occur in the leaf proteome of the GS mutants, since two different protein spots for the same sequence were identified for the enzyme fructose-bisphosphate aldolase (Additional file 1: Table S2b) and for ferredoxin (Additional file 1: Table S3b).

## Conclusions

Increasing amounts of “omics” data are available related to the response of both model and crop plants to short-term and long-term N-deficiency and other abiotic stresses (Simons *et al.* 2014) [38]. However, linking these data to a plant phenotype in terms of plant growth, development and yield remains a challenge. This is mainly because, as shown in the present study and a number of other recent investigations [66, 67, 18], when examining individually the biological systems involved, there is only a single level of complexity, which increases when their interactions are considered [68]. Moreover, there is often very little direct correlation between differences in the accumulation of metabolites, proteins and mRNA transcripts [47], due to the complexity inherent in biological networks and to the fact that both tissue and cellular compartmentation within different organs are generally not taken into account. This metabolomic study performed on the *gln1.3* and *gln1.4* mutants should also be carried out using labelled molecules in a fluxomic study [69] performed on individual organs harvested at the V and the M stage.

**Figure 2:**
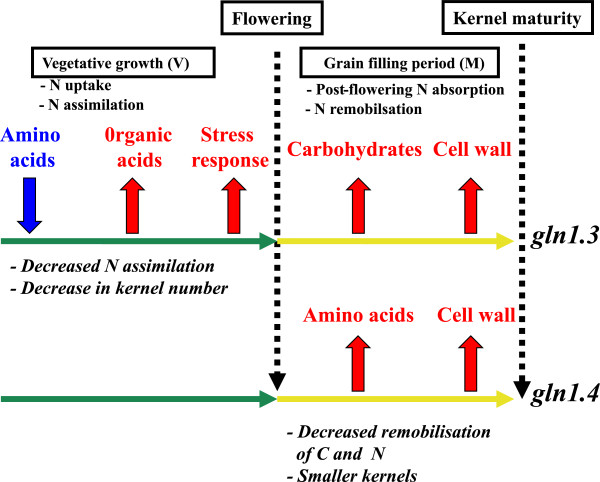
**Schematic representation of the main biological changes occurring in the leaves of the** ***gln1.3*** **and** ***gln1.4*** **mutants.** On the top of the figure is shown a schematic representation of leaf N management in maize during the developmental cycle. During vegetative growth (V), N is taken up by the roots and assimilated to build up plant cellular structures (green arrow). After flowering the N accumulated in the vegetative parts of the plant is remobilized and translocated to the developing kernels. At the same time (yellow arrow), which corresponds to the grain filling period (M), about half of the N that is translocated to the developing kernels is taken up after flowering to contribute to storage protein deposition until the kernels reach maturity. During these two main phases of plant development the large arrows indicate the different biological functions that exhibit the most significant decrease (blue arrow) or increase (red arrows) in the *gln1.3* and *gln1.4* mutants. The physiological impact of the two mutations is indicated below the green and yellow arrows in italics, which corresponds to the two main phases of leaf N management before and after flowering.

Nevertheless, the most interesting finding in this study is that in two separate mutants in which a different cytosolic GS isoenzyme has been deleted, a number of major biological processes such as C metabolism and transport, cell wall metabolism, and several metabolic pathways and stress responsive and regulatory elements, share common or specific characteristics across at least two or even three of the “omics” considered. Some of these biological processes are specific or common to the two GS mutants depending on the plant developmental stage. They are represented in Figure 2 and can be summarized as follow: in the *gln1.3* mutant the decrease in the accumulation of amino acids occurring during the V stage is due to a decrease in N assimilation resulting in a decrease in the number of kernels. During this period, the lack of amino acids induced an accumulation of organic acids that are normally used as C skeletons for amino acid synthesis, the N residues being provided by glutamine. An accumulation of stress-responsive metabolites was also observed similar to that which occurred when there is a shortage in N. Since fewer kernels were produced, carbohydrates synthesized, or exported during the grain filling period accumulated in the leaves. In the *gln1.4* mutant, the main changes observed in terms of metabolite accumulation mostly occurred during the grain filling period, when the amino acids were released during N remobilisation. These amino acids were not used to fill the kernels, thus limiting their development. During the grain filling period, an accumulation of cell wall components took place in both GS mutants, which indicates that the reduction in either kernel number or kernel size had strong repercussions on the basic structure of source organs, presumably to circumvent the decrease in storage capacity of the sink organs.

It will be necessary to exploit further “omics” data such as those obtained with the *gln1.3* and *gln1.4* mutants, in order to improve our understanding of the source to sink relationship in terms of maize productivity. This can be achieved by linking genes and metabolic functions to physiological or agronomic traits, through the construction of whole genome-scale metabolic models [70, 38]. The ultimate goal of developing such models is to provide a new tool for predicting crop yields that will allow the selection of crops adapted to lower inputs and to particular environmental conditions, while maintaining an acceptable yield. The knowledge gained from such modelling approaches could ultimately allow for the identification of key developmental and metabolic components involved in the elaboration of complex agronomic traits such as biomass and grain production.

The “omics” data obtained by growing plants under varying N conditions and by analysing genetically modified plants and mutants altered in the expression of structural or regulatory genes, can be incorporated in the model even if the genes do not have a direct link with primary C and N metabolism. Such data could provide a more accurate simulation of the impact of the genetic alteration on the metabolic interactions and fluxes throughout the plant. Subsequently, such a modelling approach could allow the identification of key reactions controlling plant growth and development under different N fertilisation or stress conditions [38].

Furthermore, the genes identified in the transcriptome studies, which were found to be the most strongly up- or down-regulated in the present investigation (listed in Tables 2 and 3), can be placed on a maize genetic map. In this way, co-localisations with QTLs for phenotypic traits related to leaf or ear growth and development, biomass production and grain yield can be identified in both vegetative and reproductive organs [1, 57]. Validation of these candidate genes exhibiting a strong alteration in their level of expression and co-localizing with phenotypic QTLs can then be undertaken using transgenic technologies, mutagenesis, or by association genetics, either at the single gene or genome-wide level.

## Methods

### Plant material

Maize wild type (WT) plants, (*Zea mays* L., genotype B73) and *gln1.3* and *gln1.4* mutant seeds in the B73 background (see [13], for the production, selection and characterization of the mutants) were grown as described in [18] in a glasshouse at the Institut National de la Recherche Agronomique, Versailles, France from May to September 2004. Three individual plants of similar size and of similar developmental stage were selected. They correspond to the three replicates used for the three different “omics” experiments. At the 10 to 11 leaf stage (45 days after sowing), the three entire youngest fully expanded leaves without the midrib were harvested and pooled as the vegetative stage (V) samples. The entire leaf below the ear without the midrib was harvested at 55 days after silking, corresponding to 65-70% of the grain filling stage and named the mature leaf developmental stage (M). At this M stage of plant development, the leaf chlorophyll content was approximately 30% lower compared to that determined at silking, which corresponds to an early stage of leaf senescence [18]. Under our experimental conditions, maturity of the ear was attained around 80 days after silking.

In the glasshouse, plants were watered several times a day with a complete nutrient solution containing 10 mM KNO_3_ as the sole N source [71]. The complete nutrient solution also contained 1.25 mM K^+^, 0.25 mM Ca^2+^, 0.25 mM Mg^2+^, 1.25 mM H_2_PO_4_^-^, 0.75 mM SO_4_^2-^, 21.5 μM Fe^2+^(Sequestrene; Ciba-Geigy, Basel, Switzerland), 23 μM B^3+^, 9 μM Mn^2+^, 0.3 μM Mo^2+^, 0.95 μM Cu^2+^, and 3.5 μM Zn^2+^.

### RNA preparation

Total RNA was extracted as described in [72] from leaves that had been stored at -80°C. Total RNAs (50 μg) for transcriptome and quantitative Real Time-Polymerase Chain Reaction (qRT-PCR) studies were treated and prepared as previously described by [18]. Reverse transcription reactions and quantitative first strands were synthesized according to [18]. Primers for qRT-PCR and RT-PCR cloning were designed from Bacterial Artificial Chromosomes (BAC) sequences found in the public maize genome databases (Maizesequence.org, PlantGDB, Genebank). The sequences of the primers used in RT-PCR and qRT-PCR are presented in Additional file 1: Table S14.

### Gene expression profiles using maize cDNA microarrays and statistical analysis of data

Whole genome leaf transcript profiling was performed using the maize 46 K arrays obtained from the maize oligonucleotide array project (http://www.maizegdb.org/microarray.php#mes) essentially as described previously in [18]. The maize 46 K spotted oligonucleotide array contains 46,000 unique probes from maize. Its detailed description, composition and gene putative annotation can be found at the Gene Expression Omnibus (GEO); (http://www.ncbi.nlm.nih.gov/geo/query/acc.cgi?acc=GPL6438).

Starting with 3 μg of total leaf RNA, non-modified amplified antisense RNA (aRNA) products were prepared using the Amino Allyl MessageAmp™ aRNA Kit (Ambion, Foster City, CA, USA). Following this, 2 μg-aliquots of aRNA were labelled using the SuperSript™ Indirect cDNA Labeling System Kit (Invitrogen, Carlsbad, CA, USA) and the purification steps were carried out using QIAquick® PCR columns (QIAGEN, Hilden, Germany). The quantity and quality of each intermediate product, including total RNA, dscDNA, aRNA and labelled targets, were evaluated using a Nanodrop ND-1000 spectrophotometer and an Agilent Technologies 2100 Bioanalyzer. Hybridisations between the maize oligonucleotide microarrays and fluorescently labelled samples were performed in MICROMAX Hybridisation Buffer III (Perkin Elmer) using the manufacturer’s hybridisation and wash conditions and a GeneTac™ HybStation (Genomic Solutions, Ann Arbor, NI, USA). Before hybridisation, 50 pmol Cy3- and 50 pmol Cy5-labelled targets were mixed, dried using compressed air and reconstituted with 115 μl of hybridisation buffer, followed by denaturing at 90°C for 3 min. Each hybridisation mixture was placed on the maize 46 K array slides mounted in the hybridisation station and the hybridisations were performed for 3 h at 65°C, followed by 3 h at 55°C, then 12 h at 50°C with gentle agitation. Thereafter, the arrays were automatically washed with the GeneTac™ washing solutions (Genomic Solutions, Ann Arbor, NI, USA) using the program for multiple automatic washes, with a flow time of 40 s. Hybridised microarrays were scanned using a GenePix 4000B Microarray Scanner (Molecular Devices, Sunnyvale, CA, USA) at 10-μm resolution and variable photomultiplier (PMT) voltage to obtain maximal signal intensities with <0.05% probe saturation. Subsequent image analysis was performed with the GenePix Pro (v6.0.1.26) software.

The transcript abundance of each of the 46, 000 unique genes in each of the three replicates for V and M leaves was determined using a mixture of all the samples (18 in total, each with the same mRNA concentration) as a reference. Statistical group comparisons were performed using multiple testing procedures to evaluate statistical significance for differentially expressed genes essentially as described in [18]. Transcriptomic data were validated by qRT-PCR analysis performed on a selected number of gene transcripts up- or down-regulated (Additional file 1: Figure S1). Non-filtered transcriptome data are presented in Additional files 2, 3, 4 and 5.

### Two-dimensional electrophoresis, gel staining, image analysis and protein identification

Total protein extraction, solubilisation, and quantification were performed as described in [73]. The frozen leaf powder (100 mg) was resuspended in acetone with 0.07% (v/v) 2-mercaptoethanol and 10% (w/v) TCA. Proteins were allowed to precipitate for 1 h at -20°C. The pellet was then washed overnight with acetone containing 0.07% (v/v) 2-mercaptoethanol. Protein resolubilisation was performed using 60 μL/mg of R2D2 buffer (5 M urea, 2 M thiourea, 2% CHAPS, 2% SB3-10, 20 mM dithiothreitol, 5 mM Tris (2-carboxyethyl) phosphine hydrochloride, 0.75% carrier ampholytes). The total protein content of each sample was evaluated using the 2-D Quant kit (Amersham Biosciences).

Solubilised proteins (300 μg) were separated on a pH 4–7 gradient Immobilised pH Gradient (IPG) strip (Amersham Biosciences) using a Protean Isoelectrofocusing (IEF) cell (Bio-Rad), as follows: Active rehydration was performed at 20°C for 13 h at 50 V; then the focusing itself was carried out. After IEF, strips were equilibrated to improve protein transfer to the two-dimensional gel (2-D gel). The second separation was performed in an 11% SDS–PAGE gel. Separation was carried out at 20 V for 1 h and subsequently at a maximum of 30 mA/gel, 120 V overnight, until the bromophenol blue front had reached the end of the gel. After SDS-PAGE, the gels were subsequently stained with colloidal Coomassie blue. Scanning was carried out at 300 dpi with a 16-bit greyscale pixel depth using an image scanner (Amersham Biosciences), and then gel images were analyzed using the Progenesis and SameSpot softwares (Nonlinear Dynamics Ltd). The SAS package (procedure GLM for one way ANOVA analysis) was used to examine modifications of individual protein spot volumes. A protein spot was selected if its variation had a p value <0.05.

Spot digestion and LC-MS/MS were performed as described in [13]. In-gel digestion was performed with the Progest system (Genomic Solution). Gel pieces were washed twice by successive separate baths of 10% acetic acid, 40% ethanol, and acetonitrile (ACN). The pieces were then washed twice with successive baths of 25 mM NH_4_CO_3_ and ACN. Digestion was subsequently performed for 6 h at 37°C with 125 ng of modified trypsin (Promega) dissolved in 20% methanol and 20 mM NH_4_CO_3_. The peptides were extracted successively with 2% trifluoroacetic acid (TFA) and 50% ACN and then with ACN. Peptide extracts were dried in a vacuum centrifuge and suspended in 20 mL of 0.05% TFA, 0.05% formic acid and 2% ACN. HPLC was performed on an Ultimate LC system combined with a Famos Autosampler and a Switchos II microcolumn switch system (Dionex). A multiple-threshold filter was applied at the peptide level: Xcorr magnitude were up to 1.7, 2.2, 3.3 and 4.3 for peptides with one, two, three and four isotopic charges respectively; peptide probability lower than 0.05, ΔCn >0.1 with a minimum of two different peptides for an identified protein. A database search was performed with Bioworks 3.3.1 (Thermo Electron). The TIGR maize gene indice database v 16, 72047*6 EST (http://maize.jcvi.org/) sequences was used.

### Metabolome analysis

All steps were adapted from the original protocol described in [74] following the procedure described in [18]. The ground frozen leaf samples (25 mg fresh weight) were resuspended in 1 ml of frozen (-20°C) Water: Chloroform: Methanol (1:1:2.5) and extracted for 10 min at 4°C with shaking at 1400 rpm in an Eppendorf Thermomixer. Insoluble material was removed by centrifugation and 900 μl of the supernatant were mixed with 20 μl of 200 μg/ml ribitol in methanol. Water (360 μl) was then added and after mixing and centrifugation, 50 μl of the upper polar phase were collected and dried for 3 h in a Speed-Vac and stored at -80°C. For derivatisation, samples were removed from -80°C storage, warmed for 15 min before opening and Speed-Vac dried for 1 h before the addition of 10 μl of 20 mg/ml methoxyamine in pyridine. The reactions with the individual samples, blanks and amino acid standards were performed for 90 min at 28°C with continuous shaking. 90 μl of N-methyl-N-trimethylsilyl-trifluoroacetamide (MSTFA) were then added and the reaction continued for 30 min at 37°C. After cooling, 50 μl of the reaction mixture were transferred to an Agilent vial for injection. For the analysis, 3 h and 20 min after derivatisation, 1 μl of the derivatised samples were injected in the Splitless mode onto an Agilent 7890A gas chromatograph (GC) coupled to an Agilent 5975C mass spectrometer (MS). The column used was an Rxi-5SilMS from Restek (30 m with 10 m Integra-Guard column). The oven temperature ramp was 70°C for 7 min, then 10°C/min up to 325°C, which was maintained for 4 min. For data processing, Raw Agilent datafiles were converted into the NetCDF format and analyzed with AMDIS (http://chemdata.nist.gov/dokuwiki/doku.php?id=chemdata:amdis). Peak areas were then determined using the quanlynx software (Waters) after conversion of the NetCDF file into the masslynx format. Statistical analyses were carried out with TMEV http://www.tm4.org/index.html. Univariate analyses by permutation (1-way ANOVA and 2-way ANOVA) were first used to select the metabolites exhibiting significant changes in their concentration.

### Availability of supporting data

The original transcriptomic data sets are presented in supplementary file 16 to 19. Proteomic and metabolomic data sets can be obtained from the corresponding author on request.

## Electronic supplementary material

Additional file 1: Table S1: Metabolites exhibiting significant changes in their concentration at the V and M stage in *gln1.3* and *gln1.4* mutants. **Table S2.** Proteins exhibiting significant decrease **(a)** and increase **(b)** in their concentration in the *gln1.3* mutant at the V stage. **Table S3.** Proteins exhibiting significant decrease **(a)** and increase **(b)** in their concentration in the *gln1.3* mutant at the M stage. **Table S4.** Proteins exhibiting significant decrease **(a)** and increase **(b)** in their concentration in the *gln1.4* mutant at the V stage. **Table S5.** Proteins exhibiting significant decrease **(a)** and increase **(b)** in their concentration in the *gln1.4* mutant at the M stage. **Table S6.** Transcripts exhibiting significant decrease **(a)** and increase **(b)** in their concentration in the *gln1.3* mutant at the V stage. **Table S7.** Transcripts exhibiting significant decrease **(a)** and increase **(b)** in their concentration in the *gln1.3* mutant at the M stage. **Table S8.** Transcripts exhibiting significant decrease **(a)** and increase **(b)** in their concentration in the *gln1.4* mutant at the V stage. **Table S9.** Transcripts exhibiting significant decrease **(a)** and increase **(b)** in their concentration in the *gln1.4* mutant at the M stage. **Table S10.** Transcripts exhibiting significant changes in *gln1.3* mutant both at the V and M stage. **Table S11.** Transcripts exhibiting significant changes in *gln1.4* mutant both at the V and M stage. **Table S12.** Transcripts exhibiting significant changes both in *gln1.3* and *gln1.4* mutant both at the V stage. **Table S13.** Transcripts exhibiting significant changes both in *gln1.3* and *gln1.4* mutant both at the M stage. **Table** **S14.** Primers used in qRT-PCR for the validation of the transcriptome study. **Figure S1.** Transcript abundance in the *gln1.3* and *gln1.4* maize (*Zea mays L.*) mutants compared to the wild type (WT) of selected genes. (PDF 2 MB)

Additional file 2: **Non-filtered transcriptome data obtained with the maize 46 k oligonuceotide array for** ***gln1.4*** **mutant in comparison to the WT at the vegetative (V) stage.** (XLSX 12 MB)

Additional file 3: **Non-filtered transcriptome data obtained with the maize 46 k oligonuceotide array for** ***gln1.3*** **mutant in comparison to the WT at the maturity (M) stage.** (XLSX 11 MB)

Additional file 4: **Non-filtered transcriptome data obtained with the maize 46 k oligonuceotide array for** ***gln1.3*** **mutant in comparison to the WT at the vegetative (V) stage.** (XLSX 12 MB)

Additional file 5: **Non-filtered transcriptome data obtained with the maize 46 k oligonuceotide array for** ***gln1.4*** **mutant in comparison to the WT at the maturity (M) stage.** (XLSX 13 MB)

## Abbreviations

C: Carbon
2-D: Two dimentional electrophoresis
GC/MS: Gas chromatography coupled to mass spectrometry
GS: Glutamine synthetase
M: Maturity stage
N: Nitrogen
NUE: Nitrogen use efficiency
QTLs: Quantitative trait loci
TCA: Tricarboxylic acid
V: Vegetative stage
WT: Wild type.
