# Supplementary material for: An integrated “omics” approach to the characterization of maize (Zea mays L.) mutants deficient in the expression of two genes encoding cytosolic glutamine synthetase
Source: BMC Genomics. 2014 Nov 20;15(1):1005. doi: 10.1186/1471-2164-15-1005 (PMC4247748; doi:10.1186/1471-2164-15-1005)
Supplement: Supplementary file 1 — Additional file 1: Table S1: Metabolites exhibiting significant changes in their concentration at the V and M stage in gln1.3 and gln1.4 mutants. Table S2. Proteins exhibiting significant decrease (a) and increase (b) in their concentration in the gln1.3 mutant at the V stage. Table S3. Proteins exhibiting significant decrease (a) and increase (b) in their concentration in the gln1.3 mutant at the M stage. Table S4. Proteins exhibiting significant decrease (a) and increase (b) in their concentration in the gln1.4 mutant at the V stage. Table S5. Proteins exhibiting significant decrease (a) and increase (b) in their concentration in the gln1.4 mutant at the M stage. Table S6. Transcripts exhibiting significant decrease (a) and increase (b) in their concentration in the gln1.3 mutant at the V stage. Table S7. Transcripts exhibiting significant decrease (a) and increase (b) in their concentration in the gln1.3 mutant at the M stage. Table S8. Transcripts exhibiting significant decrease (a) and increase (b) in their concentration in the gln1.4 mutant at the V stage. Table S9. Transcripts exhibiting significant decrease (a) and increase (b) in their concentration in the gln1.4 mutant at the M stage. Table S10. Transcripts exhibiting significant changes in gln1.3 mutant both at the V and M stage. Table S11. Transcripts exhibiting significant changes in gln1.4 mutant both at the V and M stage. Table S12. Transcripts exhibiting significant changes both in gln1.3 and gln1.4 mutant both at the V stage. Table S13. Transcripts exhibiting significant changes both in gln1.3 and gln1.4 mutant both at the M stage. Table S14. Primers used in qRT-PCR for the validation of the transcriptome study. Figure S1. Transcript abundance in the gln1.3 and gln1.4 maize (Zea mays L.) mutants compared to the wild type (WT) of selected genes. (PDF 2 MB) [file 12864_2014_6693_MOESM1_ESM.pdf]

Table S1 Metabolites exhibiting significant changes (Student t-test, P ≤ 0.05) in their concentration in *gln1.3* and *gln1.4* mutant at the V stage and at the M stage

gln1.3

V

Functional category	Metabolite	FC/WT
C metabolism	Glucose	0.04
C metabolism	Glucosopyranose	0.07
C metabolism	Pipecolate	0.11
C metabolism	Lactose	0.14
C metabolism	Mannose	0.15
C metabolism	Glucuronate	0.18
C metabolism	Galactose	0.25
C metabolism	Sorbitol	0.29
C metabolism	Butanetriol	0.42
C metabolism	Arabinose	0.43
C metabolism	Sedoheptulose	0.58
C metabolism	Erythritol	0.63
[b]CW metabolism	Quinate	0.27
CW metabolism	Shikimate	0.30
Metabolism	Salicylate	0.24
N metabolism	His	0.002
**N metabolism**	**Ser**	**0.07**
**N metabolism**	**Met**	**0.09**
N metabolism	Gly	0.13
N metabolism	Pro	0.14
N metabolism	Ile	0.16
**N metabolism**	**Val**	**0.17**
**N metabolism**	**Phe**	**0.19**
N metabolism	Triethylamine	0.19
**N metabolism**	**Thr**	**0.22**
N metabolism	Leu	0.26
N metabolism	Tyr	0.39
N metabolism	Lys	0.42
**N metabolism**	**Ala**	**0.48**
N metabolism	Trp	0.49
Polyamine	Putrescine	0.22

Functional category	Metabolite	FC/WT
C metabolism	2-Hydroxyglutarate	4.43
C metabolism	Malonate	3.74
C metabolism	Nicotinate	3.51
C metabolism	Rafinose	3.42
**C metabolism**	**Malate**	**3.11**
C metabolism	2-Oxoglutarate	2.39
C metabolism	Glycerate	2.32
C metabolism	Fumarate	2.23
**C metabolism**	**Rhamnose**	**1.97**
C metabolism	Galactinol	1.79
C metabolism	Pentonate1	1.79
**C metabolism**	**Aconitate**	**1.68**
C metabolism	Mannitol	1.53
C metabolism	Maltose	1.11
CW metabolism	Sinapinate	18.75
Metabolism	Dopamine	5.74
Metabolism	Inositol	1.17

M

Functional category	Meatbolite	FC/WT
C metabolism	Galactarate	0.001
C metabolism	Galactosylglycerol	0.13
C metabolism	Inositol-P-glycerol	0.14
**C metabolism**	**Aconitate**	**0.21**
C metabolism	Inositol-scyllo	0.23
C metabolism	Digalactosylglycerol	0.28
C metabolism	Hexonic 4	0.35
**C metabolism**	**Rhamnose**	**0.41**
C metabolism	Melibiose	0.41
C metabolism	Ribose	0.42
C metabolism	Arabinose	0.51
C metabolism	Xylose	0.66

Functional category	Metabolite	FC/WT
**C metabolism**	**Glucarate**	**5.40**
C metabolism	P-Enolpyruvate	3.30
C metabolism	Fructose	2.68
C metabolism	Glucose	2.29
C metabolism	Glucopyranose	1.65
**CW metabolism**	**Coumaroylquinate**	**70.61**
**Metabolism**	**Urate**	**1.75**
Metabolism	Phosphate	1.66
Metabolism	Salicylate	6.24
**Polyamine**	**Spermidine**	**5.47**

Common to *gln1.3* and *gln1.4* mutant at the V stage and at the M stage (green)

Common to *gln1.3* and *gln1.4* mutant at the M stage (yellow)

Red **Primary C and N metabolites in higher amount**

Blue **Primary C and N metabolites in lower amout**

Bold **Opposite accumulation between the V and M stage, bewteen *gln1.3* and *gln1.4* mutant or both**

gln1.4

V

Functional category	Metabolite	FC/WT
C metabolism	Glucosopyranose	0.33
C metabolism	Glucuronate	0.45
C metabolism	Galacturonate	0.47
C metabolism	Mannitol	0.49
C metabolism	Galactose	0.64
C metabolism	Erythritol	0.66
C metabolism	Sedoheptulose	0.69
C metabolism	Rhamnose	0.74
N metabolism	Leu	0.60
Polyamine	Putrescine	0.32
**CW metabolism**	**Coumaroylquinate**	**0.18**
CW metabolism	Quinate	0.38
CW metabolism	Shikimate	0.41

Functional category	Metabolite	FC/WT
C metabolism	Malate	2.40
C metabolism	Glycerate	2.23
C metabolism	Citraconate	1.49
**N metabolism**	**Phe**	**2.88**
**Polyamine**	**Spermidine**	**1.78**

M

Functional category	Metabolite	FC/WT
**C metabolism**	**Malate**	**0.69**
**C metabolism**	**Glucarate**	**0.50**
**C metabolism**	**Urate**	**0.46**
C metabolism	Citramalate	0.21
C metabolism	Galactarate	0.17
Metabolism	Melezitose	0.55
Metabolism	Hexonate2	0.54
Metabolism	Pipecolate	0.51
N metabolism	2-Hydroxyglutarate	0.65
N metabolism	3-OH-3-Metglutarate	0.35
CW metabolism	Quinate	0.32

Functional category	Metabolite	FC/WT
C metabolism	Glycerol-3-P	2.22
C metabolism	Mannose	2.13
C metabolism	Butanetriol	1.48
Metabolism	Salicylate	3.79
Metabolism	Dehydroascorbate	1.70
Metabolism	Threonate	1.32
**N metabolism**	**Val**	**3.45**
**N metabolism**	**Ser**	**2.96**
**N metabolism**	**Thr**	**2.73**
**N metabolism**	**Met**	**2.10**
N metabolism	Asp	1.92
**N metabolism**	**Ala**	**1.87**
N metabolism	Glu	1.59
CW metabolism	Coumarate	1.82
CW metabolism	Caffeate	1.69

FC/WT corresponds to the fold change in the mutants compared to wild type (WT) plants for metabolites exhibiting significant variations (Student t-test, *P* ≤ 0.05) in their amount. CW = cell wall.

Table S2a Proteins exhibiting significant decrease (Student t-test, *P* ≤ 0.05) in their amount in the *gln1.3* mutant at the V stage

Spot ID	Identification number	Functional category	Protein name	FC /WT
2666	TC288041	C metabolism	**Ribose-5-phosphate isomerase precursor {So} Q8RU73**	0.44
1905	TC486794	C metabolism	Phosphoribulokinase {Os} Q6Z8F4	0.19
2027	TC311169	C metabolism	Fructose-1,6-bisphosphatase, cytosolic {Sh} Q43139	0.08
1885	TC304992	C metabolism	Fructose-bisphosphate aldolase {Pa} Q6RJ32	0.16
3809	TC304752	Cell wall	Glycine-rich protein {Zm} Q41810	0.48
1966	TC280213	Cell wall	Alpha-1,4-glucan-protein synthase {Zm} P80607	0.38
1884	TC279240	Cell wall	*Cinnamyl alcohol dehydrogenase {Lp} Q8S412*	0.23
2278	TC287829	Lipid metabolism	Malonyl-CoA:ACP transacylase {Pf} Q9FR04	0.01
3147	TC461573	Metabolism	Vacuolar ATP synthase subunit C {Os} Q6L4R5	0.05
1551	TC279356	Metabolism	*ATP synthase beta chain {Zm} P00827*	0.26
3104	TC280598	Metabolism	ATP synthase subunit d, mitochondrial {At} Q9FT52	0.77
1731	TC503557	Metabolism	S-adenosylmethionine synthetase 2 {Os} P93438	0.15
2144	TC503010	Metabolism	Thiazole biosynthetic enzyme 1-2, chloroplast precursor {Zm] Q41739	0.08
1954	TC463245	N metabolism	**Glutamine synthetase root isozyme 3 (GS1-3) {Zm} P38561**	0.45
1854	TC467371	Photosynthesis	**Malate dehydrogenase [NADP], chloroplast precursor E {Zm} P15719**	0.21
2330	TC299774	Proteolysis	Proteasome subunit alpha type 1 (20S alpha) {Os} P52428	0.58
2843	TC287751	Proteolysis	**ATP-dependent protease {Os} Q50LH5**	0.28
1737	TC298239	Proteolysis	RAD23 protein {Sl} Q9STA6	0.61
3788	TC279966	Stress/Defence	Actin-depolymerizing factor 3 (ZmADF3) {Zm} Q41764	0.07
3250	TC524782	Stress/Defence	**Peroxiredoxin-2E-2, chloroplast precursor {Os} Q69TY4**	0.56
2817	TC280243	Stress/Defence	Chloroplast chapronin 21 {Vf} Q6B4V4	0.69
2748	TC285595	Stress/Defence	Ascorbate peroxidase {Os} P93404	0.36
2738	TC285595	Stress/Defence	Ascorbate peroxidase {Os} P93404	0.43
2682	TC280109	Stress/Defence	Glutathione transferase I {Zm} S03726	0.56
2800	TC460119	Stress/Defence	Glutathione s transferase IIIA {Zm} Q9ZP62	0.12
2607	TC294877	Stress/Defence	**CAXIP1-like protein {At} AY157989**	0.14
2256	TC499661	Translation	Translation initiation factor IF-3-like {Os} Q6K674	0.52
3364	TC310278	Translation	**Eukaryotic translation initiation factor 5A {Zm} P80639**	0.48
3617	TC287421	Translation	**60S acidic ribosomal protein P2B {Zm} O24415**	0.29

FC/WT corresponds to the fold change in the mutants compared to wild type (WT) plants for proteins exhibiting significant variations (Student t-test, *P* ≤ 0.05) in their amount. Proteins indicated in italics correspond to two isoforms of the same protein.
Proteins names indicated in bold were also found to be present in lower amounts in *gln1.4* mutant at the V stage.

Table S2b Proteins exhibiting significant increase (Student t-test, *P* ≤ 0.05) in their amount in the *gln1.3* mutant at the V stage

Spot ID	Identification number	Functional category	Protein name	FC /WT
2234	TC305774	C metabolism	Apospory-associated protein C-like {Os} Q6K2P3	4.72
2000	TC280370	C metabolism	Malate dehydrogenase, cytoplasmic {Zm} Q08062	1.72
2007	TC490724	C metabolism	Sedoheptulose-1,7-biphosphatase precursor {Os} AAO22559	1.46
1207	TC458298	C metabolism	Phosphoglycerate mutase {Zm} P30792	2.53
2094	TC285785	C metabolism	*Fructose-bisphosphate aldolase class-I {Os} Q53P95*	2.17
2104	TC285785	C metabolism	*Fructose-bisphosphate aldolase class-I {Os} Q53P95*	3.28
1188	TC458298	C metabolism	Phosphoglycerate mutase {Zm} P30792	2.85
1900	TC279240	Cell wall	*Cinnamyl alcohol dehydrogenase {Lp} Q8S412*	4.05
3781	TC286701	Metabolism	H+-transporting ATP synthase chain 9-like protein {Os} Q84PA4	2.43
1434	TC279356	Metabolism	*ATP synthase beta chain {Zm} P00827*	1.89
1962	TC306967	N metabolism	Glutamine synthetase {Zm} P38563 (GS1.5)	4.13
1381	TC545357	Photosynthesis	Ribulose bisphosphate carboxylase large chain precursor {Zm} P00874	1.76
1903	TC458513	Photosynthesis	Ribulose biphosphate carboxylase/oxygenase activase {Zm} Q9ZT00	1.82
2639	TC281879	Stress/Defence	Harpin binding protein 1 {Zm} Q5QJA2	2.95
2017	TC513320	Stress/Defence	Oxidoreductase-like {Os} Q656T5	2.67
2238	TC495648	Translation	**Translation initiation factor IF-3-like {Os} Q6K674**	1.96
1730	TC459353	Translation	Elongation factor Tu {0s} Q8W2C3	1.46

FC/WT corresponds to the fold change in the mutants compared to wild type WT plants for proteins exhibiting significant variations (Student t-test, *P* ≤ 0.05) in their amount. Proteins indicated in italics correspond to two isoforms of the same protein.
Proteins indicated in bold were also found to be present in higher amounts in *gln1.4* mutant at the V stage.

Table S3a Proteins exhibiting significant decrease (Student t-test, $P \leq 0.05$) in their amount in the *gln1.3* mutant at the M stage

Spot ID	Identification number	Functional category	Protein name	FC /WT
1692	TC304992	C metabolism	***Fructose-bisphosphate aldolase {Os} Q69V57***	0.00
1661	TC304992	C metabolism	***Fructose-bisphosphate aldolase {Os} Q69V57***	0.16
1394	TC311663	C metabolism	**Glucose-1-phosphate adenylyltransferase {Zm} A5GZ73**	0.16
1250	TC298164	C metabolism	Enolase 10 {Zm} P26301	0.18
1660	TC293364	C metabolism	**Phosphoribulokinase {Tae} P26302**	0.19
1680	TC313074	C metabolism	Alpha-galactosidase 1 precursor {Os} Q9FWV8	0.24
1520	TC286949	C metabolism	**NADP-specific isocitrate dehydrogenase {Os} Q9XGU8**	0.35
1796	TC311169	C metabolism	Fructose-1,6-bisphosphatase {Sh} Q43139	0.49
761	TC305088	C metabolism	**Transketolase {Zm} Q7SIC9**	0.57
768	TC279235	Development	**Actin 1 {0s} A2XLF2**	0.33
803	TC286907	Metabolism	Putative vacuolar proton-ATPase {Os} Q651T8	0.45
2455	TC305786	Metabolism	FAD/NAD(P)-binding oxidoreductase {At} Q9XI55	0.03
1914	TC303207	N metabolism	***Arginine decarboxylase {Os} Q01HV9***	0.10
1674	TC303207	N metabolism	***Arginine decarboxylase {Os} Q01HV9***	0.12
2311	TC279249	Photosynthesis	Oxygen-evolving enhancer protein 1 {Tae} P27665	0.41
2851	TC292835	Photosynthesis	**Chlorophyll a/b-binding apoprotein CP24 precursor {Zm} Q41748**	0.53
2746	TC310824	Photosynthesis	23 kDa polypeptide of photosystem II {Os} O65103	0.57
2829	BQ619330	Photosynthesis	LHCI-680, photosystem I antenna protein {Hv} Q43485	0.59
2759	TC310824	Photosynthesis	Oxygen-evolving enhancer protein 2 {Tae} Q00434	0.62
1154	TC307322	Proteolysis	Serine-type carboxypeptidase {Os} Q69ME0	0.35
1755	TC294056	Proteolysis	Protease Do-like 1, chloroplast precursor {At} O22609	0.43
2178	TC312398	Signalling	**Tyrosine phosphatase{Os} Q9LKK3**	0.26
1775	TC293174	Stress/Defence	Peroxidase 7 {Tm} Q5I3F1	0.27
2105	TC283000	Stress/Defence	**Peroxidase J {Os} Q7F1U1**	0.34
715	TC279798	Stress/Defence	**DnaK-type molecular chaperone hsp70 {Os} Q53NM9**	0.57
3391	TC307030	Stress/Defence	**OSJNBa0006A01.5 protein {Os} Q7F9L9**	0.01
2763	TC304817	Stress/Defence	**2-Cys peroxiredoxin BAS1 {OS} Q6ER94**	0.27
3017	TC299837	Stress/Defence	Germin-like protein {Zm} Q6TM44	0.46
2489	TC281878	Stress/Defence	**Harpin binding protein 1 {Zm} Q5QJA2**	0.47
1270	TC312072	Structure	**Plastid-lipid-associated protein 3 {Os} Q7XBW5**	0.16
2359	TC310158	Translation	**Elongation factor 1-delta 1 {Os} Q40680**	0.24
3354	*TC310278*	*Translation*	Eukaryotic translation initiation factor 5A {Zm} P80639	*0.55*

FC/WT corresponds the the fold change in the mutants compared to wild type (WT) plants for proteins exhibiting significant variations (Student t-test, $P \leq 0.05$) in their amount. Proteins indicated in italics correspond to two isoforms of the same protein.
Proteins indicated in bold were also found to be present in lower amounts in *gln1.4* mutant at the M stage.

Table S3b Proteins exhibiting significant decrease (Student t-test, $P \leq 0.05$) in their amount in the *gln1.3* mutant at the M stage

Spot ID	Identification number	Functional category	Protein name	FC /WT
1874	TC285785	C metabolism	*Fructose-bisphosphate aldolase class-I {Os} Q53P95*	1.74
1766	*TC292245*	*C metabolism*	**Glyceraldehyde-3-phosphate dehydrogenase {Zm} P09315**	*1.91*
1148	TC287561	C metabolism	Beta-D-glucosidase precursor {Zm} Q41761	2.31
1781	TC280370	C metabolism	Malate dehydrogenase {Zm} Q08062	2.35
1653	TC293364	C metabolism	Phosphoribulokinase {Tae} P26302	3.89
1739	TC280215	Cell wall	Alpha-1,4-glucan-protein synthase {Zm} P80607	1.81
1188	TC279356	Metabolism	*ATP synthase beta chain {Zm} P00827*	1.55
2711	TC312121	Metabolism	ATP synthase delta chain, chloroplast precursor {Sb} Q07300	2.17
1262	TC280392	Metabolism	Restorer factor 2 (RF2) {Zm} Q43274	2.23
2072	TC310669	Metabolism	***Ferredoxin {Zm} Q9SLP5***	2.24
2077	TC310669	Metabolism	***Ferredoxin {Zm} Q9SLP5***	2.41
1720	TC310869	N metabolism	Glutamine synthetase root isozyme 4 {Zm} P38562	1.61
1042	TC293513	Stress/Defence	Chaperonin CPN60-2, mitochondrial precursor {Zm} Q43298	4.01
2834	TC301296	Stress/Defence	Superoxide dismutase [Mn] 3.4 {Zm} P41980	1.50
2914	TC294699	Translation	50S ribosomal protein L10 {Os} Q9ZSS9	2.02
1971	TC300713	Translation	Ribosomal protein {Os} Q850W6	2.16
2751	TC312649	Translation	Plastid-specific 30S ribosomal protein 2 {So} P82277	2.29
2016	TC280689	Translation	Ribosomal protein L1 {Os} Q60E59	2.63
3254	TC286511	Transport	Cytochrome b6-f complex iron-sulfur subunit {Os} Q69S39	1.70

FC/WT corresponds the the fold change in the mutants compared to wild type WT plants for proteins exhibiting significant variations (Student t-test, $P \leq 0.05$) in their amount. Proteins indicated in italics correspond to two isoforms of the same protein.
Proteins indicated in bold were also found to be present in higher amounts in *gln1.4* mutant at the M stage.

Table S4a Proteins exhibiting significant decrease (Student t-test, $P \leq 0.05$) in their amount in *gln1.4* mutant at the V stage

Spot ID	Identification number	Functional category	Protein name	
2666	TC288041	C ametabolism	**Ribose-5-phosphate isomerase precursor {So} Q8RU73**	0.7
1848	TC285983	C metabolism	Phosphoglycerate kinase, cytosolic {Tae} P12783	0.3
1954	TC463245	N metabolism	**Glutamine synthetase root isozyme 3 (GS1-3) {Zm} P38561**	0.7
1854	TC467371	Photosynthesis	**Malate dehydrogenase [NADP], chloroplast precursor E {Zm} P15719**	0.1
2843	TC287751	Proteolysis	**ATP-dependent protease {Os} Q50LH5**	0.4
3250	TC524782	Stress/Defence	**Peroxiredoxin-2E-2, chloroplast precursor {Os} Q69TY4**	0.6
2607	TC294877	Stress/Defence	**CAXIP1-like protein {At} AY157989**	0.5
3364	TC310278	Translation	**Eukaryotic translation initiation factor 5A {Zm} P80639**	0.6
3617	TC287421	Translation	**60S acidic ribosomal protein P2B {Zm} O24415**	0.1
1842	TC548216	Unknown	TGB12K interacting protein 2 {Nt} Q84P56	0.6

FC corresponds to the fold change in the mutants compared to WT plants for proteins exhibiting significant variations (Student t-test, $P \leq 0.05$) in their amount.
Proteins indicated in bold were also found to be present in lower amounts in *gln1.3* mutant at the V stage.

Table 4b Proteins exhibiting significant inccrease (Student t-test, $P \leq 0.05$) in their amount in the *gln1.4* mutant at the V stage

Spot ID	Identification number	Functional category	Protein name	FC/WT
2115	TC287227	C metabolism	Fructokinase 1 {Zm} Q6XZ79	2.9
2437	TC498147	Stress/Defence	Probable plastid-lipid-associated protein 2, chloroplastic {Os} Q6K439	1.6
2256	TC499661	Translation	**Translation initiation factor IF-3-like {Os} Q6K674**	1.5
3373	TC310278	Translation	Eukaryotic translation initiation factor 5A {Zm} P80639	1.6

FC corresponds the the fold change in the mutants compared to WT plants for proteins exhibiting significant variations (Student t-test, $P \leq 0.05$) in their amount.
Proteins indicated in bold were also found to be present in lower amounts in *gln1.3* mutant at the V stage.

Table S5a Proteins exhibiting significant decrease (Student t-test, $P \leq 0.05$) in their amount in the *gln1.4* mutant at the M stage

Spot ID	Identification number	Functional category		FC/WT
1653	TC293364	C metabolism	**Phosphoribulokinase {Tae} P26302**	0.42
1520	TC286949	C metabolism	**NADP-specific isocitrate dehydrogenase {Os} Q9XGU8**	0.44
1394	TC311663	C metabolism	**Glucose-1-phosphate adenylyltransferase {Zm} A5GZ73**	0.45
761	TC305088	C metabolism	**Transketolase {Zm} Q7SIC9**	0.46
1758	TC292245	C metabolism	**Glyceraldehyde-3-phosphate dehydrogenase A, chloroplast precursor {Zm}P09315**	0.47
1660	TC293364	C metabolism	**Phosphoribulokinase {Tae} P26302**	0.51
1773	TC285785	C metabolism	**Fructose-bisphosphate aldolase class-I {OS} Q53P96**	0.56
1745	TC312652	C metabolism	Sedoheptulose-1,7-bisphosphatase {Tae} P46285	0.59
768	TC279235	Development	**Actin {Os} A2XLF2**	0.44
1674	TC303207	N metabolism	**Arginine decarboxylase {Os} Q01HV9**	0.06
1914	TC303207	N metabolism	**Arginine decarboxylase {Os} Q01HV9**	0.11
2616	TC286614	Photosynthesis	**Chlorophyll a-b binding protein M9, chloroplast precursor {Zm} P27497**	0.26
2182	TC312398	Signalling	**Putative tyrosine phosphatase {Os} Q9LKK3**	0.69
2763	TC286603	Stress/Defence	**2-Cys peroxiredoxin BAS1 {OS} Q6ER94**	0.33
2805	TC304817	Stress/Defence	Thioredoxin peroxidase {Os} O81480	0.18
2105	TC283000	Stress/Defence	**Peroxidase J {Os} Q7F1U1**	0.21
715	TC279798	Stress/Defence	**DnaK-type molecular chaperone hsp70 {Os} Q53NM9**	0.32
2800	TC304817	Stress/Defence	Thioredoxin peroxidase {Os} O81480	0.40
2489	TC281878	Stress/Defence	**Harpin binding protein 1 {Zm} Q5QJA2**	0.46
3391	TC307030	Stress/Defence	**OSJNBa0006A01.5 protein {Os} Q7F9L9**	0.63
1270	TC312072	Structure	**Plastid-lipid-associated protein 3, chloroplast precursor {Os} Q7XBW5**	0.40
2359	TC310158	Translation	**Elongation factor 1-delta 1 {Os} Q40680**	0.25

FC/WT corresponds to the fold change in the mutants compared to wild type (WT) plants for proteins exhibiting significant variations (Student t-test, $P \leq 0.05$) in their amount.
Proteins indicated in bold were also found to be present in lower amounts in *gln1.3* mutant at the M stage.

Table S5b Proteins exhibiting significant increase (Student t-test, $P \leq 0.05$) in their amount in the *gln1.4* mutant at the M stage

Spot ID	Identification number	Functional category	Protein name	FC /WT
2072	TC310669	Metabolism	**Ferredoxin {Zm} Q9SLP5**	2.20
2565	TC280815	Stress/Defence	IN2-1 protein putative expressed {Os} Q10N45	2.06
1234	TC281354	Stress/Defence	Methylmalonate semi-aldehyde dehydrogenase {Os} Q6Z4E4	2.22
1042	TC293513	Stress/Defence	Chaperonin CPN60-2, mitochondrial precursor Z{m} Q43298	3.15
1264	TC281354	Stress/Defence	Methylmalonate semi-aldehyde dehydrogenase {Zm} Q6Z4E4	3.43
2729	TC313499	Unknown	(Clone wusl1032) mRNA sequence {Zm} Q43707	3.76

FC/WT corresponds to the fold change in the mutants compared to WT plants for proteins exhibiting significant variations (Student t-test, $P \leq 0.05$) in their amount.
Proteins indicated in bold were also found to be present in higher amounts in gln1.3 mutant at the M stage.

Table S6a Trancripts exhibiting significant decrease (Student t-test, *P* ≤ 0.05) in the *gln1.3* mutant at the V stage

Identification number	Functional category	Putative annotation	FC/WT
AZM4_41292	C metabolism	ADP-glucose pyrophosphorylase small subunit {Zm} Q947C0	0.05
BM382459	C metabolism	NAD-malate dehydrogenase precursor {Nt} Q9XQP4	0.07
AI491304	C metabolism	Beta-D-xylosidase {Os} Q53MP2	0.14
TC248095	C metabolism	Putative succinate dehydrogenase flavoprotein alpha subunit {Os} Q6ZDY8	0.22
TC259550	C metabolism	Ribulose-5-phosphate-3-epimerase {Os} Q9ZTP5	0.23
TC264470	C metabolism	2 3-bisphosphoglycerate-independent phosphoglycerate mutase {Os} Q10LY9	0.26
TC254070	C metabolism	Putative dTDP-4-dehydrorhamnose reductase {Os} Q6K7R8	0.29
TC248037	C metabolism	Putative pyruvate dehydrogenase E1 alpha subunit {Bn} A8IXJ9	0.36
TC259558	C Metabolism	Putative phospho-2-dehydro-3-deoxyheptonate aldolase 1, chloroplast precursor {Os} Q75W16	0.28
AI603716	C Metabolism	Putative phospho-2-dehydro-3-deoxyheptonate aldolase 1 chloroplast precursor {Os} Q75W16	0.29
TC272153	Cell wall	Alpha-expansin 1 {Z} Q94KT7	0.23
TC272199	Cell wall	Cellulose synthase-3 {Zm} Q9LLI7	0.28
BM378652	Cell wall	Probable laccase {Zm} Q2PAJ0	0.22
TC265543	Cytoskeleton	Tubulin beta-3 chain {Zm} Q43695	0.37
TC273548	DNA repair	Putative formamidopyrimidine-DNA glycosylase 1 {Os} Q67WP0	0.36
TC253670	Lipid metabolism	Very-long-chain fatty acid condensing enzyme CUT1 like protein {At} Q0WWR0	0.15
TC271549	Lipid metabolism	Putative lipoxygenase {Zm} A1XCI0	0.24
TC261801	Lipid metabolism	Putative uncharacterized protein {Os} Q8LR39	0.37
TC269753	Metabolism	ATP synthase beta chain, mitochondrial precursor {Zm} P19023	0.10
TC249231	Metabolism	Putative vacuolar ATP synthase subunit C {Os} Q6L4R5	0.11
TC262572	Metabolism	Beta3-glucuronyltransferase {Zm} Q7XHK1	0.12
TC253743	Metabolism	Putative uncharacterized protein {Os} A3C1C7	0.13
AZM4_139479	Metabolism	Putative UDP-glucosyltransferase {Os} Q852C2	0.13
TC251023	Metabolism	Cytochrome P450 {Tae} Q70EV8	0.15
TC253757	Metabolism	Flavonoid 3',5'-hydroxylase {Gh} A5XEH3	0.16
TC271095	Metabolism	Cycloartenol synthase {As} Q6IWA4	0.19
TC256370	Metabolism	Putative indole-3-glycerol phosphate synthase {Os} Q6K307	0.20
AI795614	Metabolism	Putative cytidine deaminase {Zm} A1YLY6	0.24
BM338846	Metabolism	Dehydration-responsive family protein-like {Os} Q6EP94	0.28
TC264764	Metabolism	UbiE/COQ5 methyltransferase-like {Os} Q6Z2U2	0.29
TC273509	Metabolism	CMP-KDO synthetase {Zm} Q9M4G3	0.29
TC251368	Metabolism	Putative respiratory burst oxidase protein E {Os} Q69LJ7	0.29
TC263464	Metabolism	Putative polynucleotide phosphorylase {Os} Q69LE7	0.30
AZM4_113030	Metabolism	Putative hydroxyanthranilate hydroxycinnamoyltransferase 2 {Os} Q6ZBI7	0.33
TC255762	Metabolism	mRNA-binding protein precursor {Nt} Q7X998	0.35
BM500657	Metabolism	Putative cinnamoyl-CoA reductase {OS} Q69U05	0.35
TC271607	Metabolism	Chalcone--flavonone isomerase {Zm} Q08704	0.37
TC209230	Metabolism	Hydrolase, TatD family like protein {Os} Q6Z130	0.34
TC264498	N Metabolism	Cysteine synthase {Os} Q9XEA9	0.12
TC256306	Nucleotide binding	Isoflavone reductase related protein {Pc} O81355	0.07
TC273424	Nuclesome assembly	Histone acetyl transferase {Zm} Q8VWI2	0.31
TC268742	Plant development	S-adenosyl-L-methionine: phosphoethanolamine N-methyltransferase {Zm} A7XZC6	0.35
TC249748	Polyamine biosynthesis	Aminopropyl transferase {Os} Q7XAP2	0.18
TC260199	Proteolysis	Ubiquitin-associated (UBA)/TS-N domain-containing protein-like {Os} Q6K653	0.23
TC247886	Proteolysis	Ubiquitin-like protein SMT3 {Os} P55857	0.34
TC271428	Proteolysis	Serine carboxypeptidase II-1 precursor {Hv} P55747	0.21
TC272405	Proteolysis	Bowman-Birk serine protease inhibitor {Tae} P81713	0.25
CF633259	Proteolysis	Putative uncharacterized protein {Os} A2YM57	0.26
TC248802	Proteolysis	Polyprotein {Sugarcane mosaic virus} Q09EZ1	0.34
TC265459	Proteolysis	Chloroplast nucleoid binding protein {Bs} Q2L3E7	0.38
TC260128	RNA silencing	Leafbladeless1 {Zm} A1Y2B7	0.33
TC192492	Signalling	Peptidyl-prolyl cis-trans isomerase {Os} A2WYA7	0.04
TC252728	Signalling	Putative acid phosphatase {Hv} Q9M4D6	0.10
TC271920	Signalling	Putative tubby-like protein {Os} Q6Z2G9	0.11
TC213804	Signalling	Putative CBL-interacting protein kinase {Os} Q6Z9F4	0.13
TC255710	Signalling	Calcium-dependent protein kinase-related kinase {Zm} O23797	0.16
BG841682	Signalling	Peptidyl-prolyl cis-trans isomerase{Os} A2WYA7	0.17
TC271919	Signalling	Putative tubby-like protein {Os} Q6Z2G9	0.17
TC250426	Signalling	Auxin-binding protein precursor {Zm} P13689	0.19
TC207366	Signalling	Putative calcium-dependent protein kinase {Os} Q6I587	0.19
BG873986	Signalling	Os05g0353500 protein{Os} Q0DIX6	0.21
TC272457	Signalling	Serine/threonine-protein kinase SAPK3 {Os} A2ZAB5	0.27
BG874174	Signalling	Galactokinase {Ps} Q5ZP80	0.30
TC249147	Signalling	Zinc finger and C2 domain protein-like {Os} Q7XIV3	0.31
TC215575	Signalling	Receptor protein kinase {Os} Q5JN26	0.32
TC206632	Signalling	Auxin-responsive protein {OS} P0C132	0.34
TC250894	Signalling	Histidine kinase 1 {Zm} Q9FRY7	0.37
TC269627	Stress/Defence	Putative DnaJ like protein {Os} Q6F3B0	0.06
TC264842	Stress/Defence	101 kDa heat shock protein {Tae} Q334I0	0.13
TC270076	Stress/Defence	Putative thioredoxin peroxidase {Os} O81480	0.14
TC273388	Stress/Defence	MutT/nudix-like {Os} Q6Z2I1	0.16
TC264843	Stress/Defence	101 kDa heat shock protein {Zm} Q9S822	0.17
TC213439	Stress/Defence	Metallothionein-like protein 1 {Zm} P30571	0.17
TC248663	Stress/Defence	Pore-forming toxin-like protein Hfr-2 {Tae} Q4JEV5	0.19
TC251553	Stress/Defence	Putative peroxidase {Os} Q9FYP0	0.22
AZM4_93135	Stress/Defence	Putative SHOOT1 protein {Os} Q69LC0	0.27
TC204802	Stress/Defence	L-ascorbate peroxidase {Os} Q10N21	0.37
TC205681	Transcription	NAC3 protein {Os} Q7EZT1	0.13
TC267841	Transcription	B3 DNA binding domain containing protein expressed {Os} Q2QMT2	0.14
TC271561	Transcription	Protein rough sheath 2 {Zm} Q9S7B2	0.17
TC276381	Transcription	BHLH transcription factor(GBOF-1)-like {Os} Q84QW1	0.23
BG841242	Transcription	Putative bZIP protein HY5 {Os} Q6ZHT8	0.28
BG836491	Transcription	Putative transcription factor EREBP1 {Os} A3RL57	0.29
TC273757	Transcription	M22 {Zm} Q70ZY3	0.30
TC252076	Transcription	bZIP transcription factor RF2b {Os} Q6S4P4	0.30
TC261460	Translation	Eukaryotic translation initiation factor 4E-2 {Zm} O81482	0.29
TC255353	Translation	Putative ubiquitin / ribosomal protein CEP52 {Os} Q7XHS4	0.31
TC258930	Translation	Putative 60S ribosomal protein L11 {Os} P52855	0.31
BI478752	Translation	Putative translational inhibitor protein {O} Q8H4B9	0.32
TC256186	Transport	Putative integral membrane protein {Os} Q688W0	0.02
BM500946	Transport	Os09g0555100 protein {Os} Q0IZR3	0.06
TC274040	Transport	Putative cyclic nucleotide gated ion channel {Hv} Q4VDN3	0.08
TC272004	Transport	Kinesin-like protein {Os} Q9FW70	0.13
AZM4_8141	Transport	Putative lipid transfer protein {Hv} P93191	0.13
TC250451	Transport	Sugar transporter protein {Ac} A4GXC8	0.14
TC206341	Transport	Synaptobrevin-like protein {Os} Q69WS1	0.15
CF024160	Transport	ETCHED1 protein {Zm} Q6KF34	0.24
AZM4_69077	Transport	Putative impotin alpha 1b {Os} AZM4_69077	0.25
TC261773	Transport	Putative ABC transporter {Os} Q69S79	0.27
TC271071	Transport	Phosphate/phosphoenolpyruvate translocator protein {Os} Q8S2F4	0.29
TC266526	Transport	ABC transporter G family member 15 {At} Q8RWI9	0.32
AZM4_38886	Transport	putative amino acid transporter {Os} Q8S7G6	0.32
TC273825	Transport	Putative ubiquinol--cytochrome-c reductase {At} Q2RA72	0.38
TC256063	Unknown	Mitochondrial transcription termination factor-like protein {Os} Q6K5F5	0.19
TC277727	Unknown	Expressed protein {Os} Q2R123	0.25
**AI665454**	**Unknown**	**Putative uncharacterized protein {Os} A2Z2L6**	**0.07**

FC/WT corresponds to the fold change in the mutants compared to wils type (WT) plants for transcripts exhibiting significant variations (Student t-test, *P* ≤ 0.05) in their amount. Genes indicated in bold were used for validation by qRT-PCR.

Table S6b Trancripts exhibiting significant increase (Student t-test, $P \leq 0.05$) in the *gln1.3* mutant at the V stage

Identification number	Functional category	Putative annotation	FC/WT
TC272022	C metabolism	Beta-glucosidase {Mt} A8TVR1	3.01
TC272124	C metabolism	Putative pyrophosphate-dependent phosphofructokinase alpha subunit {At} Q0WNI9	4.69
TC273540	C metabolism	Putative class III chitinase {Os} Q7G8Q1	6.09
TC278448	C metabolism	Phosphoribulokinase precursor {Os} P26302	29.41
BG319752	Cell wall	Xyloglucan endotransglycosylase homolog {Zm} Q42446	9.14
TC277283	Cell wall	Glycoside hydrolase family 28 protein {Os} Q10B12	11.37
TC279125	Cell wall	Hydroxyproline-rich glycoprotein-like protein {Os} Q67UA8	12.36
AZM4_22327	Cytoskeleton	Profilin 4 {Zm} O22655	2.84
BF729443	DNA binding	Putative LHY protein {Os} Q6ZD85	4.84
CB278146	DNA integration	Prpol {Zm} Q8SA85	3.06
ZRSgTERTOOT18402	DNA replication	Putative reverse transcriptase {Os} ZRSgTERTOOT18402	3.33
TC249210	DNA topology	DNA topoisomerase II {Os} A2X8M2	8.01
TC248075	Lipid metabolism	Acyl-CoA thioester hydrolase-like {Os} Q69MT1	4.87
TC252426	Lipid metabolism	3-hydroxybutyryl-CoA dehydrogenase-like protein {At} Q9LDF5	11.03
TC255020	Lipid metabolism	3-hydroxy-3-methylglutaryl-CoA reductase {Os} O04188	46.64
CA403071	Metabolism	Putative sorbitol dehydrogenase {Os} Q6ZBH2	2.60
TC270212	Metabolism	Putative NUDIX hydrolase {Os} Q6ATR4	2.69
TC204944	Metabolism	Putative sorbitol dehydrogenase {Os} Q6ZBH2	2.69
BM501462	Metabolism	Catalase {Zm} P12365	3.03
TC269736	Metabolism	Putative HGA1 {Os} Q5Z8T7	3.43
TC274083	Metabolism	Iron/ascorbate-dependent oxidoreductase {Hv} Q7XJ26	4.12
TC253148	Metabolism	Uncharacterized protein {Os} A2X8Q0	4.46
TC255584	Metabolism	Putative iron deficiency protein Ids3 {Os} Q69LD9	4.70
TC252043	Metabolism	Catalase isozyme 2 {Zm} P12365	4.71
TC253202	Metabolism	Indole-3-glycerol phosphate lyase {Zm} Q9FQ77	5.21
AZM4_101226	Metabolism	ferredoxin {Zm} Q9SLP6	5.35
TC278653	Metabolism	Putative cytochrome P450 71C4 {Os} Q6ZD83	5.79
CD441119	Metabolism	Putative sorbitol dehydrogenase {O} Q6ZBH2	5.90
CB179185	Metabolism	Trans-cinnamate 4-monooxygenase {Ca} O81928	6.40
BI992412	Metabolism	Putative uncharacterized protein {Os} A2X8P7	6.77
CD438149	Metabolism	Putative sorbitol dehydrogenase {Os} Q6ZBH2	6.82
BI992914	Metabolism	Putative silverleaf whitefly-induced protein 1 {Os} Q6ZGS9	9.87
TC270254	Metabolism	Putative mitochondrial F0 ATP synthase D chain {Os} Q9FT52	10.54
TC213118	Metabolism	Sesquiterpene cyclase {Zm} Q9FEF5	10.69
TC249105	Metabolism	Ferredoxin 1 {Zm} P27787	13.43
TC195262	Metabolism	Isoleucyl-tRNA synthetase-like {Os} Q67WM1	21.73
BG837402	Metabolism	Putative ethylene-forming enzyme {Os} Q8SB89	3.86
TC274941	N Metabolism	Asparaginase {Hv} Q9FUG8	3.41
TC191421	Nuclesome assembly	Histone H2B {Zm} P40280	2.92
TC248918	Nuclesome assembly	Histone H1-like protein HON101 {Zm} Q8SB89	2.97
BI542963	Nuclesome assembly	Histone H2A {Zm} P40280	3.48
TC190930	Nuclesome assembly	Histone H2A {Zm} P40280	3.92
CF056912	Nuclesome assembly	Histone deacetylase {Zm} Q9ZTP8	6.74
AZM4_72986	Nuclesome assembly	Histone H2B.2 {Zm} P30756	17.14
TC262308	Photosynthesis	Putative glycolate oxidase {Os} Q6YT73	3.12
TC248507	Photosynthesis	Photosystem I reaction centre subunit N chloroplast precursor {Hv} S35159	5.68
**TC260113**	**Photosynthesis**	**23 kDa polypeptide of photosystem II {Nt} Q04126**	**283.37**
TC249049	Proteolysis	26S proteasome regulatory particle triple-A ATPase subunit6 {Os} Q9FEB6	2.88
TC270878	Proteolysis	Zinc finger protein {Os} Q5JL96	3.19
TC261397	Proteolysis	Kelch repeat-containing F-box-like {Os} Q53NA8	4.81
TC269559	Proteolysis	Cinful1 polyprotein {Zm} Q8W1D4	14.13
TC266142	Proteolysis	Avr9/Cf-9 rapidly elicited protein-like {Os} Q6K5K9	33.18
TC261195	Proteolysis	26S proteasome regulatory subunit-like protein {Os} Q69Q88	34.37
**TC199171**	**Proteolysis**	**Putative uncharacterized protein {Os} Q75K52**	**79.56**
TC276882	Purine biosynthesis	Phosphoribosylaminoimidazole-succinocarboxamide synthase {Os} Q6YXG8	3.08
TC261848	Ribosome biogenesis	Putative brix domain-containing protein {Os} Q69L77	2.68
TC251967	Secondary metabolism	Chalcone isomerase-like {Os} Q6EQW2	7.37
TC251968	Secondary metabolism	Chalcone isomerase-like {Os} Q6EQW2	28.12
TC256631	Signalling	Pherophorin-C2 protein precursor {Cr} Q3HTK5	2.94
TC276517	Signalling	Putative RPT2 {Os} Q69P69	2.95
TC250773	Signalling	Dual-specificity phosphatase protein {Os} Q6DL00	2.97
CF006353	Signalling	putative zinc finger protein {At} O04261	3.18
TC251357	Signalling	Putative ethanolamine kinase 1 {Os} Q69QD7	3.19
TC260058	Signalling	Pollen-specific protein C13 precursor {Zm} P33050	3.26
TC273781	Signalling	Putative Ras-related protein Rab {Os} Q8S7J3	3.35
TC250774	Signalling	Dual-specificity phosphatase protein {Os} Q6DL00	3.68
TC272464	Signalling	TAK33 {Ta} Q9ATQ4	3.83
TC250843	Signalling	Putative protein phosphatase-2C {Os} O82470	4.17
TC254588	Signalling	Os07g0578200 protein {Os} Q0D566	4.19
TC278080	Signalling	RPT2-like protein {Hv} Q84YF6	4.77
TC249354	Signalling	Putative protein kinase CK2 regulatory subunit CK2B3 {Zm} Q9FQF6	5.08
TC259714	Signalling	Shaggy-like kinase etha {Os} Q5Z7J0	6.08
CA402100	Signalling	ADP-ribosylation factor-like protein {Nt} Q08IJ1	15.80
TC276810	Signalling	Putative calcium binding protein {Os} Q652U8	22.14
**TC270988**	**Signalling**	**Protein phosphatase type-2C {Zm} Q9FQY2**	**23.61**
TC249498	Signalling	Peptidyl-prolyl cis-trans isomerase {Os} A2WYA7	26.76
TC250399	Signalling	Pyruvate dehydrogenase kinase isoform 2 {Zm} O82424	30.98
TC273009	Stress/Defence	Putative calreticulin {Os} Q8LJ85	2.70
TC272680	Stress/Defence	Wound responsive protein {Os} A6N0L4	2.84
TC199909	Stress/Defence	Catalase isozyme 2 {Zm} P12365	3.18
TC260819	Stress/Defence	Brown planthopper-induced resistance protein 1 {Os} Q6V9H6	3.39
TC259284	Stress/Defence	Gigantea-like protein {Os} A6N180	3.46
TC267654	Stress/Defence	Putative heat shock protein {Os} Q8RVR9	3.54
TC274706	Stress/Defence	Peroxidase precursor {Cc} Q43417	3.63
NP288631	Stress/Defence	Glutathione S-transferase GST 33 {Zm} Q9FQA6	3.73
TC265405	Stress/Defence	Pathogen-related protein {Os} Q945E9	4.01
TC271690	Stress/Defence	Sugar-starvation induced protein {Zm} Q41855	4.16
TC258684	Stress/Defence	ABA- and ripening-inducible-like protein{Zm} Q41730	4.28
TC260968	Stress/Defence	Thioredoxin-like 1 {Os} Q10M18	4.29
TC199226	Stress/Defence	Glutathione S-transferase GST 24 {Zm} Q9FQB5	4.32
TC276723	Stress/Defence	Zeaxanthin cleavage dioxygenase chromoplast precursor {Cs} Q84K96	4.47
TC276317	Stress/Defence	Peroxidase precursor {Cc} Q43416	4.58
TC249083	Stress/Defence	Cysteine proteinase inhibitor {Zm} Q30KW7	4.78
AZM4_5610	Stress/Defence	Low-molecular-weight cysteine-rich protein LCR69 precursor {At} S30578	5.10
CD436680	Stress/Defence	Proteinase inhibitor {Os} O24225	5.13
TC255599	Stress/Defence	DnaJ protein-like {Os} Q6Z899	5.84
TC249019	Stress/Defence	Putative uncharacterized protein{Os} A2X0A2	5.92
TC252674	Stress/Defence	Glutathione S-transferase GST 21 {Zm} Q9FQB8	6.17
CF024860	Stress/Defence	Cysteine proteinase inhibitor {Zm} Q30KW6	6.39
TC276520	Stress/Defence	Putative uncharacterized protein {Os} A2X0A2	6.88
AW056325	Stress/Defence	heat shock protein 17.2 {Zm} Q43701	7.25
TC264087	Stress/Defence	AvrRpt2-induced protein 2-like {Os} Q6Z2W4	11.97
TC260732	Transcription	Helix-loop-helix-like protein {Os} Q6ZKI8	2.68
TC260535	Transcription	Transcription factor RAU1 {Os} Q8S490	2.96
TC194557	Transcription	putative 15.9 kDa subunit of RNA polymerase II {Os} O48890	3.40
AZM4_80187	Transcription	Putative H-protein promoter binding factor-2a {Os} Q8S7W1	4.05
TC278406	Transcription	Transcription factor MYB42 {Zm} Q2A700	4.66
AZM4_1726	Transcription	Myb transcription factor protein {Os} Q56UT4	5.26
TC270942	Transcription	Typical P-type R2R3 Myb protein {Zm} Q8RUC5	6.60
TC264482	Transcription	Putative transcription factor (Myb-like DNA-binding domain) {Os} PF00249	7.71
TC264476	Transcription	MA3 domain-containing protein {Os} Q10PT6	14.19
TC255750	Transcription	MLIP 15 {Zm} Q41833	430.37
NP672375	Translation	Putative ribosomal protein L17 {Os} Q6K1W6	2.98
TC258948	Translation	Putative ribosomal protein S3a, cytosolic {Os} P49397	3.51
AI987264	Translation	Putative aspartate-tRNA ligase {Os} Q6ZHC3	3.65
TC269732	Translation	Putative 60S ribosomal protein {Os} Q8LKU3	5.16
TC248070	Translation	Putative 60S ribosomal protein L1 {Os} Q7Y1I5	6.38
TC271736	Translation	Ribosomal protein S21-like protein {Os} Q6YUV4	18.77
CA403087	Translation	Ribosomal protein S21-like protein {Os} Q6YUV4	20.80
TC248254	Translation	40S ribosomal protein S10 {Os} Q9AYP4	4.11
AZM4_114699	Transport	Putative chloride channel protein {Os} Q6ZDC2	2.66
TC261760	Transport	Putative amino acid transport protein {Os} Q6ESP5	3.44
TC253416	Transport	Potassium outward rectifying channel{Zm} Q5EEQ2	3.61
TC251375	Transport	Membrane protein-like {Os} Q6EQH5	4.38
TC272026	Transport	Putative VAMP-associated protein {Os} Q8LDM1	4.40
CF045733	Transport	SEC14 cytosolic factor-like {Os} Q6ER71	4.99
TC276034	Transport	Major facilitator superfamily protein {Zm} A5YMH6	5.09
**TC248805**	**Transport**	**Mannitol transporter putative expressed {Os} Q10QG5**	**5.72**
TC268922	Transport	ADP-ribosylation factor {Os} P36397	14.06
TC253650	Transport	Putative nitrite transporter {Os} Q5Z6P7	22.38
TC258661	Transport	Syntaxin {Os} Q6YVJ8	31.33
TC251793	Transport	Peroxisomal biogenesis factor 11 {Mt} A2Q5C8	2.62
TC253688	Transposon	Transposase {Zm} Q5UDR1	43.62
TC253014	Transposon	Putative AC9 transposase {Zm} P03010	3.00
TC271800	Transposon	Putative transposase {Os} Q2QMM6	4.24
T18705	Transposon	Activator-like transposable element {Pg} T18705	4.47
TC260041	Unknown	Uncharacterized protein{Mt} Q45NM5	5.14
TC275787	Unknown	MATH domain containing protein expressed{Os} Q2QMP1	5.76
TC260712	Unknown	Putative Tic62 protein {Os} Q69SX2	6.53

FC/WT corresponds to the fold change in the mutants compared to wild type (WT) plants for transcripts exhibiting significant variations (Student t-test, $P \leq 0.05$) in their amount. Genes indicated in bold were used for validation by qRT-PCR.

Table S7a Trancripts exhibiting significant decrease (Student t-test, $P \leq 0.05$) in the *gln1.3* mutant at the M stage

Identification number	Functional category	Putative annotation	FC/WT
TC273598	C metabolism	Beta-glucosidase 31 {Os} B7F7K7	0.08
TC248095	C metabolism	Putative succinate dehydrogenase flavoprotein alpha subunit {Os} Q6ZDY8	0.16
TC249389	C metabolism	Putative arabinogalactan-like protein {Os} Q5QLS1	0.18
BM501271	C metabolism	Putative beta-1 3-glucanase {Os} Q6Z9Y9	0.03
BG835945	C metabolism	Putative beta-1,3-glucanase {Os} Q6Z9Y9	0.04
BG836367	C metabolism	Putative beta-1,3-glucanase precursor {Os} Q6Z9Y9	0.21
TC272199	Cell wall	Cellulose synthase-3 {Zm} Q9LLI7	0.11
AZM4_104563	Cytoskeleton	Putative dynein light chain {Os} Q09IX9	0.17
TC272001	Cytoskeleton	Kinesin-like protein {Os} Q7XCW8	0.31
TC192225	Development	Floral organ regulator 1 {Os} Q8GT94	0.13
TC249811	Development	Probable chromatin-remodeling complex ATPase chain {Os} Q7G8Y3	0.26
TC262161	Lipid metabolism	Putative acyltransferase {Os} Q6YY78	0.20
TC253670	Lipid metabolism	Very-long-chain fatty acid condensing enzyme CUT1 like protein {At} Q0WWR0	0.21
TC249712	Lipid metabolism	Beta-ketoacyl-ACP synthase {Hv} Q40027	0.23
TC272080	Lipid metabolism	Hydroperoxide lyase {Zm} Q6QHT5	0.24
AZM4_124731	Metabolism	Putative P450 monooxygenase {Os} BAD09944.1	0.01
TC266256	Metabolism	S-adenosylmethionine decarboxylase {Sa} A4GXE8	0.02
TC249231	Metabolism	Putative vacuolar ATP synthase subunit C {Os} Q6L4R5	0.04
AZM3_14088	Metabolism	Putative cytochrome P450 {Os} Q9AS41	0.05
TC269768	Metabolism	Putative dihydropyrimidine dehydrogenase {Os} Q8S3J6	0.05
TC255650	Metabolism	Putative trehalose-6-phosphate synthase {Os} Q6Z548	0.06
CD966502	Metabolism	Putative dihydropyrimidine dehydrogenase {Os} Q6Z744	0.06
BG837711	Metabolism	Putative cytochrome P450 {Os} Q9LXX7	0.08
TC251023	Metabolism	Cytochrome P450 {Tae} Q70EV8	0.09
CD995318	Metabolism	Putative dihydropyrimidine dehydrogenase {Os} Q6Z744	0.09
CF633665	Metabolism	Glycosyltransferase{Sb} Q5QPY7	0.09
TC217855	Metabolism	Esterase PIR7B {Os} A2WYS7	0.09
TC262102	Metabolism	Putative cytochrome b5 {Bc} Q8H293	0.13
AZM4_76918	Metabolism	Putative Cyt-P450 monooxygenase {Os} BAD37942.1	0.14
TC267145	Metabolism	Molybdenum cofactor sulfurase protein -like {Os} Q653D7	0.15
**TC261275**	**Metabolism**	**2-isopropylmalate synthase B {Os} Q2QXY9**	**0.19**
TC263090	Metabolism	Os07g0194500 protein {Os} Q0D7Z6	0.21
TC255765	Metabolism	Putative glucosyltransferase {OS} Q5VRT6	0.22
TC253520	Metabolism	Os07g0291800 protein{Os} Q0D761	0.25
TC252666	Metabolism	Epoxide hydrolase {Ac} Q8H289	0.27
TC263872	Metabolism	S-adenosylmethionine:2-demethylmenaquinone methyltransferase-like protein {Os} Q8W0C5	0.30
TC259185	mRNA splicing	Putative uncharacterized protein {Os} A2Y8C5	0.02
TC191426	N metabolism	Aspartate transaminase {Os} P37833	0.17
TC261390	N metabolism	Glutamine synthetase root isozyme 3 {Zm} P38561	0.23
TC252405	N metabolism	Glutamine synthetase root isozyme 3 {Zm} P38561	0.24
TC247927	Nucleosome assembly	Histone H4 {Os} Q0JHY5	0.26
TC264256	Nucleotide biosynthesis	Aminoimidazolecarboximide ribonucleotide transformylase {Os} Q6ZKK4	0.07
TC260829	Nucleotide metabolism	Nucleotide pyrophosphatase precursor {Os} Q42974	0.26
TC269709	Pathogenesis	Putative r40c1 proteine {Os} Q40705	0.21
TC249748	Polyamines biosynthesis	Aminopropyl transferase {Os} Q7XAP2	0.12
CF633259	Proteolysis	Putative uncharacterized protein {OS} A2YM57	0.06
TC250942	Proteolysis	Putative prolylcarboxypeptidase {Os} Q5ZAM8	0.12
BQ744608	Proteolysis	Cathepsin b-like cysteine proteinase 3 {Os} A6N1G1	0.15
CD940779	Proteolysis	Cathepsin B {Tae} Q03106	0.16
TC278353	Proteolysis	Putative serine type endopeptidase inhibitor {Zm} A3FMA3	0.18
TC259689	Proteolysis	Cysteine protease {Zm} Q43705	0.19
TC260199	Proteolysis	Ubiquitin-associated (UBA)/TS-N domain-containing protein-like {Os} Q6K653	0.19
TC276468	Proteolysis	Cysteine protease 2 {Zm} Q10717	0.20
TC247886	Proteolysis	Ubiquitin-like protein SMT3 {Os} P55857	0.22
CF635931_root	Proteolysis	OSJNBa0058G03.7 protein {Os} Q7X5X9	0.23
TC271428	Proteolysis	Serine carboxypeptidase II-1 precursor {Hv} P55747	0.30
TC205049	Proteolysis	Cystatin I precursor {Zm} P31726	0.32
AZM4_59457	Proteolysis	Cysteine endopeptidase {Os} Q9SXM1	0.38
TC253728	Signalling	Phospholipase D alpha 1 {Zm} Q43270	0.16
TC192492	Signalling	Peptidyl-prolyl cis-trans isomerase {Os} A2WYA7	0.01
TC207366	Signalling	Putative calcium-dependent protein kinase {Os} Q6I587	0.08
TC271920	Signalling	Putative tubby-like protein {Os} Q6Z2G9	0.08
BG841682	Signalling	Peptidyl-prolyl cis-trans isomerase{Os} A2WYA7	0.08
TC271919	Signalling	Putative tubby-like protein {Os} Q6Z2G9	0.09
BG874174	Signalling	Galactokinase {PS} Q5ZP80	0.10
TC272912	Signalling	Elicitor-responsive protein {OS} Q25AG5	0.12
TC267287	Signalling	ZCN15 {Zm} A8WES6	0.19
AZM4_126821	Signalling	BTH-induced protein phosphatase 1 {Os} AZM4_126821	0.22
TC249791	Signalling	Protein phosphatase 2C-like {Os} Q7F0H8	0.24
TC249790	Signalling	Protein phosphatase 2C-like {Os} Q7F0H8	0.25
TC192383	Signalling	Putative ethylene-responsive small GTP-binding protein {Os} Q84TA8	0.30
TC272908	Signalling	Putative uncharacterized protein {Pt} A9PBY9	0.32
TC270384	Signalling	Protein phosphatase 2C {Zm} Q6IV73	0.39
TC208483	Stress/Defence	Acidic class I chitinase {Zm} Q41794	0.07
TC273540	Stress/Defence	Putative class III chitinase {Os} Q7G8Q1	0.13
TC271423	Stress/Defence	Glutathione S-transferase GST 18 {Zm} Q9FQC1	0.01
TC259276	Stress/Defence	Heat shock protein 70 {Zm} Q6XNL6	0.02
AZM4_125367	Stress/Defence	Glutathione S-transferase {Os} AAM94536.1	0.02
TC264842	Stress/Defence	101 kDa heat shock protein {Tae} Q334I0	0.05
TC254765	Stress/Defence	Glutathione S-transferase GST 34 {Zm} Q9FQA5	0.07
TC213439	Stress/Defence	Metallothionein-like protein 1 {Zm} P30571	0.11
TC196442	Stress/Defence	DNAJ domain-containing; methylation-controlled J protein {Os} Q5ZCC4	0.16
TC252043	Stress/Defence	Catalase isozyme 2 {Zm} P12365	0.18
TC274919	Stress/Defence	Glutathione S-transferase GST 31 {Zm} Q9FQA8	0.19
TC264843	Stress/Defence	101 kDa heat shock protein {Zm} Q9S822	0.20
TC249778	Stress/Defence	Dehydrin {Zm} Q41824	0.20
TC249300	Stress/Defence	Superoxide dismutase {Zm} P23345	0.22
BQ485999	Stress/Defence	Heat shock protein 16.9 {Pg} Q40865	0.23
AI065909	Stress/Defence	18 Kd heat shock protein {Hv} Q40057	0.23
CD961676	Stress/Defence	Low temperature and salt responsive protein related cluster {Pg} Q5MJ27	0.25
CD219294	Stress/Defence	Desiccation responisve element-binding protein {Sb} A9LNM4	0.27
TC260273	Stress/Defence	Luminal binding protein 2 precursor {Zm} P24067	0.28
TC210344	Stress/Defence	Putative glyoxalase II {Os} Q5QLQ5	0.28
TC259808	Stress/Defence	Hypoxia induced protein {OS} Q2QYP0	0.36
TC250781	Stress/Defence	Sulfiredoxin-like protein {Os} Q45GC6	0.36
TC261595	Transcription	Putative homeodomain protein {OS} Q60EM7	0.27
TC266129	Transcription	Putative transcription activator RF2a {Os} Q10LT0	0.04
CF029576	Transcription	SCARECROW gene regulator, putative related cluster {Os} Q53MB4	0.07
TC255556	Transcription	Putative transcription factor {Os} Q75LJ4	0.10
TC267841	Transcription	B3 DNA binding domain containing protein expressed {Os} Q2QMT2	0.15
TC263379	Transcription	MYB transcription factor {Cr} A1DR87	0.18
AZM4_38275	Transcription	Putative DNA-binding protein {At} Q9CA64	0.20
TC261714	Transcription	NAM (No apical meristem)-like protein {Os} Q9FK44	0.31
TC248635	Translation	Eukaryotic translation initiation factor 2 alpha subunit {Os} Q10MT2	0.01
TC258948	Translation	Putative ribosomal protein S3a {OS} P49397	0.04
TC192123	Translation	Putative aspartate-tRNA ligase {Os} Q6ZHC3	0.20
TC274040	Transport	Cyclic nucleotide-gated ion channel 4 {Hv} Q4VDN3	0.03
TC269753	Transport	ATP synthase beta chain, mitochondrial precursor {Zm} P19023	0.05
TC256186	Transport	Putative integral membrane protein {Os} Q688W0	0.06
TC273931	Transport	Putative peptide transport protein {Os} Q7XDJ1	0.08
AI979477	Transport	Ammonium transporter 1-3 {Os} Q947M9	0.09
TC206341	Transport	Synaptobrevin-like protein {Os} Q69WS1	0.14
TC271073	Transport	Phosphate/phosphoenolpyruvate translocator protein-like {Os} Q8S2F4	0.18
TC247906	Transport	Protein transport protein SEC61 gamma subunit {Os} P38385	0.19
TC261773	Transport	Putative ABC transporter {Os} Q69S79	0.20
TC196541	Transport	ABC transporter {At} Q6GYA9	0.20
TC269251	Transport	Putative chloride channel protein {Os} Q6Z673	0.20
TC271071	Transport	Phosphate/phosphoenolpyruvate translocator protein-like {Ors} Q8S2F4	0.21
BM338689	Transport	Ran-binding protein {Tae} A0MA43	0.29
TC253336	Transport	Major facilitator superfamily antiporter {Os} Q2RAW8	0.32
TC271976	Transport	Os12g0133100 protein {Os} Q0IQB0	0.32
CF007590	Unknown	Putative uncharacterized protein {Os} A2WLZ0	0.01
TC257902	Unknown	Anthocyanin biosynthesis regulatory protein Pl1 {Zm} Q6QP46	0.03
BG458838	Unknown	Expressed protein {Os} Q6AU54	0.03
TC255042	Unknown	Putative pentatricopeptide (PPR) repeat-containing protein {Os} Q6K7U1	0.07
AZM4_136178	Unknown	Putative uncharacterized protein {Os} Q6ERW6	0.09
TC265398	Unknown	Putative uncharacterized protein {OS} Q5Z5F9	0.12
TC255859	Unknown	Putative uncharacterized protein W1110ERIPDM {Os} Q949B5	0.21
TC256063	Unknown	Mitochondrial transcription termination factor-like protein {Os} Q6K5F5	0.24
AW519999	Unknown	LEM3 family/CDC50 family protein expressed {Os} Q10SJ9	0.27
TC274207	Unknown	Expressed protein-like protein {Sb} Q84YE8	0.37

FC/WT corresponds to the fold change in the mutants compared to wild type (WT) plants for transcripts exhibiting significant variations (Student t-test, $P \leq 0.05$) in their amount.
Genes indicated in bold were used for validation by qRT-PCR.

Table S7b Trancripts exhibiting significant increase (Student t-test, $P \leq 0.05$) in the *gln1.3* mutant at the M stage

Identification number	Functional category	Putative annotation	FC/WT
TC272203	C metabolism	Beta-galactosidase {Os} A3ABI3	3.10
AI947855	C metabolism	Dihydrolipoamide S-acetyltransferase {Zm} Q9SWR9	3.13
TC261141	C metabolism	Endoglucanase 8 precursor {OS} Q6K7G9	3.81
TC272061	C metabolism	Os01g0130400 protein {Os} Q0JQZ2	4.23
TC266651	C metabolism	Raffinose synthase protein {At} Q9FND9	7.01
TC266531	C metabolism	Glycosyl hydrolases family 16 protein expressed {Os} Q10SX3	8.65
TC269702	C metabolism	Arabinogalactan protein {Nt} Q9FUL8	10.03
TC271833	C metabolism	Granule bound starch synthase IIa {Zm} A4URH2	10.80
TC261572	C Metabolism	Aldose reductase {Zm} A2T1W7	3.48
TC262118	C Metabolism	Aldose reductase {Zm} A2T1W7	8.57
AZM4_23955	Cell division	Cyclin-like protein {Os} AZM4_23955	3.06
TC263855	Cell wall	Glyoxal oxidase {Os} Q8S198	3.10
TC261243	Cell wall	Proline-rich protein precursor {Zm} Q9ZNY1	4.51
TC270841	Cell wall	Expansin-B9 precursor {Zm} Q07154	5.17
TC264354	Cell wall	Putative beta-glucan binding protein {Os} Q6H5B5	5.20
TC259352	Cell wall	Exopolygalacturonase precursor {Zm} P35339	6.06
TC276164	Cell wall	Putative pectinesterase {Os} Q69PI0	6.23
TC260411	Cell wall	Expansin-A26 precursor {Os} Q2QP13	6.35
TC270569	Cell wall	Pectinesterase family protein expressed {OS} Q2QZJ9	6.56
TC260477	Cell wall	Pectinesterase family protein expressed {Os} Q10MG7	6.73
TC248060	Cell wall	Pectate lyase {Zm} Q43862	7.01
TC259833	Cell wall	Expansin A-32 precursor {Os} Q6YYW5	7.15
CF011440	Cell wall	Exopolygalacturonase precursor {Zm} P35339	7.50
TC258899	Cell wall	Expansin B-1 precursor {Zm} P58738	8.17
AI987316	Cell wall	Exopolygalacturonase precursor {Zm} P26216	8.27
TC251465	Cell wall	Pectin methylesterase-like protein {Zm} O24596	8.62
AW289103	Cell wall	Exopolygalacturonase precursor {Zm} P26216	11.61
TC192449	Cell wall	Pectate lyase {Zm} Q43861	12.36
TC259350	Cell wall	Exopolygalacturonase precursor {Zm} P35339	15.19
AW400251	Cell wall	Exopolygalacturonase precursor {Zm} P26216	15.73
TC259350	Cell wall	Exopolygalacturonase precursor {Zm} P35339	17.51
TC277283	Cell wall	Glycoside hydrolase family 28 protein {Os} Q10B12	23.09
TC279125	Cell wall	Hydroxyproline-rich glycoprotein-like protein {Os} Q67UA8	29.29
TC271640	Cytoskeleton	tubulin beta-4 chain{Zm} Q41782	3.05
TC249583	Cytoskeleton	Actin depolymerizing factor 6 {Os} Q7XSN9	5.48
TC249210	DNA topology	DNA topoisomerase II {Os} A2X8M2	14.50
TC270501	Genetic information processing	RNA recognition motif family protein expressed {Os} Q10MR0	19.74
TC249787	Lipid metabolism	Fiddlehead putative expressed {Os} Q10QW1	3.53
TC258708	Lipid metabolism	Putative stearoyl-acyl-carrier protein desaturase {Zm} Q3HUP7	3.54
TC249785	Lipid metabolism	Fiddlehead-like protein {Os} Q10QW1	4.45
TC248075	Lipid metabolism	Acyl-CoA thioester hydrolase-like {Os} Q69MT1	30.40
TC255020	Lipid metabolism	3-hydroxy-3-methylglutaryl-CoA reductase {Os} O04188	33.09
**TC260211**	**Lipid metabolism**	[a]**Allene oxide cyclase {Zm} Q6RW09**	**33.17**
TC252587	Metabolism	Cinnamoyl-CoA reductase {Zm} Q84J56	2.84
TC249331	Metabolism	Phytoene synthase 2 {Zm} Q6EI12	3.40
CF016910	Metabolism	Putative aminotransferase AGD2 {Os} Q6VMN8	3.75
TC262802	Metabolism	Protein F14N23.19 {At} Q9SY73	3.77
TC249424	Metabolism	Cinnamyl alcohol dehydrogenase {Lp} Q8S412	4.06
TC279016	Metabolism	Putative phosphoethanolamine methyltransferase {Os} Q5SDQ0	4.25
TC259296	Metabolism	Herbicide safener binding protein 1 {Zm} O49010	4.53
BM500657	Metabolism	Putative cinnamoyl-CoA reductase {OS} Q69U05	4.53
TC274161	Metabolism	Carbonyl reductase -like protein {Os} Q7XIR1	5.47
TC276138	Metabolism	Putative uncharacterized protein {Vv} A5B456	6.16
TC209457	Metabolism	Cytosolic aldehyde dehydrogenase RF2C {Zm} Q8S532	6.91
AZM4_101226	Metabolism	Ferredoxin {Zm} AZM4_101226	10.19
TC276770	Metabolism	Geranylgeranyl diphosphate synthase {Os} Q94ID7	22.55
TC252413	Metabolism	Cinnamyl alcohol dehydrogenase {Lp} Q8S411	22.61
TC249078	Metabolism	Cytochrome P450 monooxygenase CYP72A27 {Zm} Q8LGN4	60.42
TC250386	N Metabolism	Glutamate decarboxylase {Os} Q5F1L4	6.91
AZM4_72986	Nucelosome assembly	Histone H2B.2 {Zm} P30756	26.47
TC269707	Pathogenesis	r40g2 protein {Os} Q6Z4N6	3.24
TC275387	Photosynthesis	Chlorophyll a/b-binding protein CP24 precursor {Vr} Q9XQB6	4.10
TC259341	Photosynthesis	Carbonic anhydrase {Zm} Q41728	4.80
TC259391	Proteolysis	Katanin {Os} Q7XXR9	5.05
TC256776	Proteolysis	Putative nucleoid DNA-binding protein cnd41, chloroplast {Os} Q69P39	9.49
TC249853	Proteolysis	27K protein {Tae} Q7Y1Z2	9.72
TC249219	Proteolysis	26S proteasome regulatory particle non-ATPase subunit3 {Os} Q8W3N4	10.12
TC266142	Proteolysis	Avr9/Cf-9 rapidly elicited protein-like {Os} Q6K5K9	19.49
TC261195	Proteolysis	26S proteasome regulatory subunit-like protein {Os} Q69Q88	20.68
TC251967	Secondary metabolism	Chalcone isomerase-like {Os} Q6EQW2	12.41
TC200056	Secondary metabolism	4-coumarate--CoA ligase 4CL1 {Lp} Q9M7S3	12.58
TC254689	Secondary metabolism	Terpene synthase {Zm} Q84ZW8	14.02
TC251968	Secondary metabolism	Chalcone isomerase-like {Os} Q6EQW2	44.04
AZM4_71169	Signalling	Zinc finger protein {Os} BAD19334.1	2.90
TC263931	Signalling	Protein phosphatase type 2-C Zm} A3F4K2	2.97
AI065635	Signalling	Protein kinase domain containing protein {OS} Q10LW3	3.33
TC263275	Signalling	Photomorphogenesis repressor protein-like {At} Q9LJR3	3.39
TC250858	Signalling	Expansin-like A1 precursor {Os} Q10S70	3.55
TC247967	Signalling	Floral organ regulator 1 {Os} Q8GT94	4.09
TC249983	Signalling	CCCH-type zinc finger protein-like {OS} Q9FU27	4.48
TC275385	Signalling	Putative brassinosteroid insensitive 1-associated receptor kinase 1 {OS} Q5VQL9	4.56
TC249354	Signalling	Putative protein kinase CK2 regulatory subunit CK2B3 {Zm} Q9FQF6	4.75
AZM4_35643	Signalling	Putative zinc finger protein {At} O04261	4.77
TC263963	Signalling	Putative C2 domain-containing protein {Os} Q6ZBX9	4.83
TC261915	Signalling	EF-hand Ca2+-binding protein CCD1 {Tae} Q9LLM1	5.44
TC250843	Signalling	Putative protein phosphatase-2C {Os} O82470	6.20
TC191458	Signalling	Pollen specific protein C13 precursor {Zm} P33050	6.42
TC272988	Signalling	Minor allergen alt a7 {Os} A6MZE9	8.33
TC249885	Signalling	Peptidyl-prolyl cis-trans isomerase CYP19-4 precursor {At} Q8LDP4	9.29
TC264439	Signalling	Zinc finger protein family-like {Os} Q6ZKY1	10.83
TC276810	Signalling	Putative calcium binding protein {Os} Q652U8	13.63
TC249498	Signalling	Peptidyl-prolyl cis-trans isomerase {Os} A2WYA7	61.45
TC250399	Signalling	Pyruvate dehydrogenase kinase isoform 2 {Zm} O82424	157.42
TC270969	Stress/Defence	Low-molecular-weight cysteine-rich protein LCR66 precursor {At} Q9C947	2.83
TC251736	Stress/Defence	Putative GMPase {Os} Q941T9	2.90
CD970573	Stress/Defence	6-4 photolyase {Os} Q6Z6D9	3.04
TC216807	Stress/Defence	Fibroin heavy chain (Fib-H) (H-fibroin)-like protein - {Os} Q69PC6	3.09
CF024860	Stress/Defence	Cystatin II {Zm} Q41825	3.14
TC252527	Stress/Defence	RNA binding protein {Os} Q2QSR7	3.27
TC275710	Stress/Defence	glutathione S-transferase GST 37 {Zm} Q9FQA2	3.59
TC265311	Stress/Defence	Os06g0716100 protein {Os} Q0D9H0	3.69
TC266166	Stress/Defence	Putative uncharacterized protein S126P21.2 {Sb} Q8L434	4.20
TC252674	Stress/Defence	Glutathione S-transferase GST 21 {Zm} Q9FQB8	4.76
TC255599	Stress/Defence	DnaJ protein-like {Os} Q6Z899	6.04
TC252419	Stress/Defence	Hydroxyproline-rich glycoprotein DZ-HRGP precursor {Vc} Q9SBM1	6.21
TC276214	Stress/Defence	Putative heat shock protein 70 {Zm} Q6XNL6	6.60
BM736896	Stress/Defence	Protein IN2-1 {Zm} P49248	7.71
TC274553	Stress/Defence	22.7 kDa class IV heat shock protein precurso {Zm} P19244	8.24
BM347819	Stress/Defence	Beta-glucosidase aggregating factor precursor {Zm} Q9LL87	8.56
TC272986	Stress/Defence	Hydroxyproline-rich glycoprotein DZ-HRGP precursor {Vc} Q9SBM1	8.79
TC274187	Stress/Defence	Hydroxyproline-rich glycoprotein DZ-HRGP precursor {Vc} Q9SBM1	8.80
TC270824	Stress/Defence	Putative shock protein SRC2 {Os} Q0JMR3	11.76
TC249646	Stress/Defence	Mucin-like protein {Zm} Q6YSY6	13.01
TC249645	Stress/Defence	Mucin-like protein {Os} Q6YSY6	14.30
TC255578	Stress/Defence	Plastid division protein precursor {Os} Q6Z729	15.67
TC264087	Stress/Defence	AvrRpt2-induced protein 2-like {Os} Q6Z2W4	35.66
AI065741	Stress/Defence	Thaumatin-like cytokinin-binding protein {Os} Q2QND8	47.43
TC257116	Transcription	M21 {Zm} Q84V76	2.87
TC201896	Transcription	Chitin-inducible gibberellin-responsive protein {Os} Q8GVE1	3.32
TC261982	Transcription	Ethylene-responsive element binding factor {Os} Q6J832	4.11
TC250035	Transcription	Putative zinc finger transcription factor {Os} Q6L4N4	5.08
BG320207	Transcription	Putative ethylene-responsive transcriptional coactivator-like {Rr} Q8S4Q2	5.08
TC257815	Transcription	AP2 domain-containing protein AP28 {Os} Q7X649	6.05
TC250265	Transcription	WRKY DNA binding domain containing protein {Os} Q2QMN4	9.80
TC196307	Transcription	Putative pirin-like protein {Os} Q75LB9	12.18
CF056912	Transcription	Histone deacetylase {Zm} Q9ZTP8	14.72
TC270142	Transcription	Auxin response factor 1 {Os} Q2QQX6-2	18.62
AI987264	Translation	Putative aspartate-tRNA ligase {Os} Q6ZHC3	3.14
BM417335	Translation	17S ribosomal RNA gene and flanks {Zm} MZERG17S	4.13
**TC248617**	**Translation**	**Elongation factor 1-delta 1 {Os} Q40680**	**5.72**
TC248070	Translation	Putative 60S ribosomal protein L1 {Os} |Q7Y1I5	7.54
TC249011	Translation	Putative 40S ribosomal protein S26 {Os} P49216	12.38
CA403087	Translation	Ribosomal protein S21-like protein {Os} Q6YUV4	22.52
AI065444	Translation	40S ribosomal protein S26 {Os} P49216	23.65
TC271736	Translation	Ribosomal protein S21-like protein {Os} Q6YUV4	32.63
TC253650	Transport	Putative nitrite transporter {Os} Q5Z6P7	2.77
TC197830	Transport	Na+/H+ antiporter {Tae} Q5QIU6	3.09
TC248810	Transport	Sorbitol-like transporter {Gm} Q7XA50	3.63
TC248805	Transport	Mannitol transporter putative expressed {Os} Q10QG5	3.71
TC259158	Transport	Beta adaptin-like {Os} Q5ZEA1	4.07
CD999219	Transport	Mannitol transporter putative expressed {OS} Q10QG5	4.09
TC265585	Transport	Putative uncharacterized protein {Os} A2WJN2	4.20
TC263969	Transport	SNAP25 homologous protein SNAP33 {At} Q9S7P9	5.47
TC270254	Transport	ATP synthase subunit D {Os} Q9FT52	7.18
BI096457	Transport	Putative chromate transport protein {Ms} Q4A6M3	10.50
TC271645	Transport	Chloroplast glucose-6-phosphate/phosphate translocator {Ps} A4UTS2	11.94
TC268922	Transport	ADP-ribosylation factor {At} P36397	12.42
TC277223	Transport	Putative monosaccharide transporter 1 {Os} Q69S90	16.03
TC249105	Transport	Ferredoxin 1 {Zm} P27787	19.36
CA402100	Transport	ADP-ribosylation factor-like protein {Nt} Q08IJ1	30.33
TC258661	Transport	Syntaxin {Os} Q6YVJ8	32.93
TC253014	Transposition	Putative AC9 transposase {Zm} P03010	3.43
T18705	Transposition	Activator-like transposable element {Pg} T18705	5.24
TC253688	Transposition	Transposase {Zm} Q5UDR1	29.82
TC249299	Unknown	Proline-rich protein precursor {Zm} Q9SBX4	3.04
TC205982	Unknown	Putative uncharacterized protein {Os} A2Y860	3.17
CF006353	Unknown	Putative zinc finger protein {At} O04261	3.33
CF637976	Unknown	Putative uncharacterized protein {OS} A2YIS9	3.58
TC260941	Unknown	PM5 protein-like {Os} Q0JNE6	3.81
CF632260_root	Unknown	Putative uncharacterized protein {OS} A2X7W5	4.97
TC257842	Unknown	Expressed protein {Os} Q6AVT8	5.23
TC260041	Unknown	Putative uncharacterized protein {Mt} Q45NM5	5.58
BM351379	Unknown	Putative uncharacterized protein {Os} A2WQB8	10.57
TC276236	Unknown	Streptococcal hemagglutinin-like protein {Os} Q6EN70	12.51
CF045733	Unknown	SEC14 cytosolic factor-like {Os} Q6ER71	12.52
TC265100	Unknown	Putative phosphate-induced protein 1 {Os} Q0DXF4	23.23
TC270417	Unknown	Dormancy/auxin associated protein expressed {Os} Q10LM8	31.93
TC268559	Unknown	Putative uncharacterized protein {Os} A2WKY1	4.44
**TC199171**	**Unknown**	**Putative uncharacterized protein {Os} Q75K52**	**18.49**
TC252472	Unknown	Orf protein {Zm} Q41809	2.77

FC/WT corresponds to the fold change in the mutants compared to wild type (WT) plants for transcripts exhibiting significant variations (Student t-test, $P \leq 0.05$) in their amount
Genes indicated in bold were used for validation by qRT-PCR

Table S8a Trancripts exhibiting significant decrease (Student t-test, $P \leq 0.05$) in the *gln1.4* mutant at the V stage

Identification number	Functional category	Putative annotation	FC/WT
TC250487	C metabolism	Putative trehalose-6-phosphate synthase {At} Q9C9W6	0.08
TC262118	C Metabolism	Putative aldose reductase {Os} Q0DHM8	0.09
TC210146	Lipid metabolism	H0523F07.8 protein {Os} Q01JQ5	0.04
TC253728	Lipid metabolism	Phospholipase D alpha 1 {Zm} Q43270	0.33
BM380813	Lipid metabolism	Phospholipase D nu-2 {Os} Q8SAG4	0.39
TC249033	Metabolism	Os09g0518700 protein {OS} Q0J0B2	0.14
TC261275	Metabolism	2-isopropylmalate synthase B {Os} Q2QXY9	0.15
TC260608	Metabolism	Putative threonine synthase {Os} Q6L4H5	0.31
TC192679	Metabolism	CHCH domain containing protein expressed {Os} Q10MU1	0.35
TC275071	mRNA splicing	Predicted protein {Pp} A9SFP2	0.19
TC271428	Proteolysis	Serine carboxypeptidase II-1 precursor {Hv} P55747	0.24
TC251667	Ribosome biogenesis	H/ACA ribonucleoprotein complex subunit 1-like protein {Os} Q2R242	0.30
TC270490	Signalling	Zinc finger protein-like {Os} Q5N7K2	0.12
TC261446	Signalling	Putative CRK1 protein {Os} Q93W00	0.04
TC261340	Signalling	Putative C2 domain-containing protein {Os} Q6ZBX9	0.20
TC263639	Signalling	Kinase binding protein {Os} Q0KIW5	0.21
TC210905	Signalling	Response regulator {Zm} Q9ZQW0	0.27
TC197759	Stress/Defence	rRNA N-glycosidase {Zm} Q41851	0.01
TC198868	Stress/Defence	Putative interferon-related protein {Os} Q6ZIP6	0.02
TC265311	Stress/Defence	DnaJ protein homolog-like {Os} Q6YT03	0.07
TC262220	Stress/Defence	Peroxiredoxin Q, chloroplast precursor {Tae} Q5S1S6	0.13
TC264552	Stress/Defence	Dehydration-induced protein {Tae} Q52PK2	0.13
TC204522	Stress/Defence	Metallothionein-like protein type 2 {Os} A9XB13	0.20
TC248665	Stress/Defence	Pore-forming toxin-like protein Hfr-2 {Tae} Q4JEV5	0.21
AZM4_113200	Stress/Defence	DnaJ protein, putative {At} AZM4_113200	0.28
TC217530	Transcription	Homeodomain leucine zipper protein 16 {Os} Q6Q502	0.06
CF637160	Translation	60S acidic ribosomal protein P3 {Zm} O24413	0.01
TC248642	Translation	40S ribosomal protein S21 {Zm} P93626	0.09
TC248254	Translation	40S ribosomal protein S10 {Os} Q9AYP4	0.18
AZM4_123234	Translation	Ribosomal protein L35A {Zm} AZM4_123234	0.25
AZM4_8141	Transport	Putative lipid transfer protein {Os} AZM4_8141	0.09
TC264581	Transport	Putative uncharacterized protein {Os} Q6F325	0.13
TC248296	Transport	Nonspecific lipid-transfer protein precursor {Zm} P19656	0.33
BM380114	Transposon	TNP2 {Os} Q948C7	0.03
TC199089	Unknown	Expressed protein {Os} Q10J01	0.04
TC260702	Unknown	Os01g0222700 protein {Os} Q0JPH5	0.08
TC274742	Unknown	Putative uncharacterized protein {Os} Q7F229	0.11
**AI665454**	**Unknown**	**Putative uncharacterized protein {Os} A2Z2L6**	**0.11**
TC258612	Unknown	Wali7 protein {Tae} Q43661	0.12
TC251581	Unknown	H0717B12.5 protein {Os} Q01JB7	0.12
TC257271	Unknown	Unknown protein {Os} A2Z8W2	0.13
TC276137	Unknown	Expressed protein {Os} Q2QYG0	0.14
CD958015	Unknown	Os09g0364800 protein {Os} Q0J2A0	0.17
AZM4_89978	Unknown	Unknown protein, contains F-box domain, PF00646 {Os} AZM4_89978	0.21
TC276840	Unknown	Unknown protein {Os} Q60D96	0.24
TRSiRGRR00000016	Unknown	Putative senescence-associated protein {Ps} TRSiRGRR00000016	0.29
AZM4_94659	Unknown	OSJNBa0072D21.8 {Os} AZM4_94659	0.30
TRSiRGRR00000016	Unknown	Putative senescence-associated protein {Ps} TRSiRGRR00000016	0.33
TC248100	Unknown	Putative uncharacterized protein {Os} A2YE66	0.33
TC260626	Unknown	Putative uncharacterized protein {Sb} Q8LKT8	0.34
TC259287	Unknown	Importin alpha-like protein {At} O49600	0.36
TC274227	Unknown	Os01g0128800 protein {Os} Q0JR00	0.36
AZM4_2542	Unknown	Unknown protein {Os} AZM4_2542	0.36

FC/WT corresponds to the fold change in the mutants compared to wild type (WT) plants for transcripts exhibiting significant variations (Student t-test, $P \leq 0.05$) in their amount.

Genes indicated in bold were used for validation by qRT-PCR.

Table S8b Trancripts exhibiting significant increase (Student t-test, $P \leq 0.05$) in the *gln1.4* mutant at the V stage

**Identification number**	**Functional category**	**Putative annotation**	**FC/WT**
BM072721	C metabolism	Carbonic anhydrase {Zm} Q41728	7.85
TC253829	Cell wall	Glycine-rich protein-like {Os} Q69TG9	4.20
BM380216	Cell wall	Glycine-rich cell wall structural protein precursor {At}P27483	36.64
TC250571	Metabolism	Putative CbxX protein {Os} Q6ETX1	2.64
TC266765	Metabolism	Putative iron/ascorbate-dependent oxidoreductase {Os} Q658E2	7.80
TC266666	Metabolism	NAD dependent epimerase/dehydratase family protein {Os} Q10CW5	24.91
TC266788	Metabolism	Putative NADPH HC toxin reductase {Os} Q7X6N6	107.17
TC260041	Nucleic acid binding	Uncharacterized protein {Os} Q45NM5	4.38
TC270249	Nucleic acid binding	Putative oligouridylate binding protein {Zm} Q8H6I9	7.05
**TC260113**	**Photosynthesis**	**23-kDa polypeptide photosystem II {Nt} Q04126**	**200.22**
TC261397	Proteolysis	Kelch repeat-containing F-box-like {Os} Q6H8E0	3.07
TC259721	Proteolysis	Cysteine protease component of protease-inhibitor complex {Zm} Q9SLY9	8.52
TC199171	Proteolysis	Putative uncharacterized protein {Os} Q75K52	67.99
TC264476	RNA metabolic process	MA3 domain-containing protein {Os} Q10PT6	5.57
CF006353	rRNA-processing	Programmed cell death protein 2 {Os} Q10MP9	3.00
TC273433	Signalling	OSJNBa0027P08.19 {Os} Q7XUX3	2.86
TC276666	Signalling	Protein kinase family protein {Os} Q2QXA8	4.18
TC259714	Signalling	Shaggy-like kinase etha {Os} Q5Z7J0	7.48
TC259877	Signalling	Calmodulin {Zm} Q7DLR7	8.02
TC262845	Signalling	MAP kinase 4 {Zm} Q9ZWJ6	11.55
TC259358	Signalling	Type 2 non specific lipid transfer protein precursor {Tae} Q2PCC5	12.57
**TC270988**	**Signalling**	**Protein phosphatase type-2C {Zm} Q9FQY2**	**21.34**
TC273537	Stress/Defence	Heat shock protein 17.2 {Zm} Q43701	4.92
TC270817	Stress/Defence	Dehydrin {Zm} Q41824	5.19
TC258611	Stress/Defence	22 kDa drought-inducible protein {So} Q6EBV8	5.69
AW055947	Stress/Defence	ZmGR2c {Zm} Q9ZWI4	8.66
TC261286	Stress/Defence	Metallo-beta-lactamase-like {Os} Q6K9T9	10.02
CD990526	Stress/Defence	LIP5 {Os} O48673	15.74
TC195262	Stress/Defence	Isoleucyl-tRNA synthetase-like {Os} Q67WM1	23.25
TC270237	Translation	Elongation factor 1 alpha {Zm} Q9FYV3	4.51
ZRSmOTOT00201701	Translation	Ribosomal protein-like {Os} ZRSmOTOT00201701	6.16
TC272126	Translation	Putative uncharacterized protein {Ps} A9NTN5	60.23
TC250470	Transport	Mitochondrial FAD carrier {Cm} A6YTC9	2.71
TC251793	Transport	Peroxisomal biogenesis factor 11 {Mt} A2Q5C8	3.39
TC194260	Transport	Vacuolar sorting receptor homolog {Zm} P93645	4.49
TC274040	Transport	Putative cyclic nucleotide gated ion channel {Os} Q60EI8	4.73
**TC248805**	**Transport**	**Mannitol transporter putative expressed {Os} Q10QG5**	**9.04**
TC259563	Transport	Ubiquinol-cytochrome C reductase complex ubiquinone-binding protein {Os} Q9LDS7	65.76
BE050166	Unknown	OSE3 {Os} Q9M4Y6	3.89
TC262979	Unknown	Os01g0579800 protein {Os} Q0JLR3	2.87
TC268439	Unknown	Unknown protein {Os} Q6Z406	3.00
BM334819	Unknown	Putative uncharacterized protein OJ1651_D06.1 {Os} Q60EN2	3.15
TC198218	Unknown	Putative uncharacterized protein {Os} Q7F229	3.16
TC250447	Unknown	Putative uncharacterized protein {Os} A2WYF6	3.25
BG842375	Unknown	OSJNBb0016D16.5 protein {Os} Q7FAP0	4.05
TC274110	Unknown	Putative uncharacterized protein {Os} A2Z9Q5	4.78
TC261614	Unknown	Putative uncharacterized protein {Os} A2XH87	5.68
TC251337	Unknown	Expressed protein {Os} Q2QYM8	6.32
TC252511	Unknown	OSE3 {Os} Q9M4Y6	6.85
TC262924	Unknown	Putative uncharacterized protein {Os} A2Y599	7.69
TC260627	Unknown	Putative uncharacterized protein {Sb} Q8LKT8	13.00
TC200462	Unknown	Putative uncharacterized protein {Os} Q75IJ0	13.59
TC207014	Unknown	BRI1-KD interacting protein 103 {Os} Q762B4	2.60
TC271771	Unknown	CHY zinc finger family protein {Os} Q337P2	4.37

FC/WT corresponds to the fold change in the mutants compared to wild type (WT) plants for transcripts exhibiting significant variations (Student t-test, $P \leq 0.05$) in their amount.
Genes indicated in bold were used for validation by qRT-PCR.

Table S9a Trancripts exhibiting significant decrease (Student t-test, $P \leq 0.05$) in *gln1.4* mutant at the M stage

Identification number	Functional category	Putative annotation	FC/WT
TC250487	C metabolism	Putative trehalose-6-phosphate synthase {At} Q9C9W6	0.06
TC205106	C metabolism	PRm 3 {Zm} P93518	0.07
TC208483	C metabolism	Acidic class I chitinase {Zm} Q41794	0.08
BM349195	C metabolism	Glucose-6-phosphate isomerase {Ac} A0LTY7	0.29
TC248047	C Metabolism	Aconitate hydratase {Os} Q6YZX6	0.25
TC254327	C Metabolism	UDP-glucose-4-epimerase {Zm} Q7XZQ2	0.42
AZM4_1068	Cell wall	Cellulose synthase-like protein OsCslE1 {OS} Q944E4	0.38
TC269680	Cytoskeleton	Profilin 5 {Zm} Q9FR39	0.24
TC272001	Cytoskeleton	Kinesin-like protein {Os} Q7XCW8	0.29
TC205202	Cytoskeleton	Profilin 5 {Zm}Q9FR39	0.32
BM380365	Development	Putative uncharacterized protein {OS} A2XF19	0.13
AZM4_24572	Development	putative gibberellin 20-dioxygenase {Os} Q69LD8	0.28
AZM4_96	DNA integration	Putative polyprotein {Zm} Q8SA93	0.41
TC266118	Genetic information processing	Putative splicing factor {Os} O22826	0.11
TC253728	Lipid metabolism	Phospholipase D alpha 1 {Zm} Q43270	0.16
**TC261275**	**Metabolism**	**2-isopropylmalate synthase B {Os} Q2QXY9**	**0.03**
TC273949	Metabolism	Cytochrome b5 reductase isoform II {Zm} Q8W2K4	0.13
TC262102	Metabolism	Putative cytochrome b5 {Bc} Q8H293	0.17
TC263090	Metabolism	Os07g0194500 protein {Os} Q0D7Z6	0.18
TC260608	Metabolism	MutT/nudix-like {Os} Q6Z2I1	0.26
TC273389	Metabolism	Glucosyltransferase-like {Os} Q8LJ11	0.26
CF631261	Metabolism	Putative cytochrome P450 71C4 {Os} Q6ZD83	0.27
TC278653	Metabolism	Putative carbamoyl phosphate synthetase {Os} Q0JLU1	0.31
TC273502	Metabolism	NifU-like domain containing protein {OS} Q10MC1	0.31
BM078443	Metabolism	Putative prolyl 4-hydroxylase alpha subunit {Os} Q6AVM9	0.36
TC252189	Metabolism	Hydroxymethylglutaryl-CoA lyase {Os} Q2RAU5	0.40
TC252103	Metabolism	NADPH-cytochrome P450 oxydoreductase {Os} A6MZJ5	0.45
TC273888	Metabolism	NADPH-cytochrome P450 oxydoreductase {Os} A6MZJ5	0.46
TC268472	mRNA processing	U2 snRNP auxiliary factor, small subunit {Os} Q9ZQW9	0.28
TC254835	N Metabolism	Putative threonine synthase {Os} Q6L4H5	0.18
TC270563	N Metabolism	Putative aspartate aminotransferase {At} Q9C969	0.41
TC193294	N metabolism	Glutamine synthetase root isozyme 4 {Zm} P38562	0.31
TC209156	N metabolism	Glutamine synthetase root isozyme 4 {Zm} P38562	0.38
BG320061	Photosynthesis	Photosystem I reaction center subunit XI {Hv} P23993	0.29
TC258872	Polyamine biosynthesis	S-adenosylmethionine decarboxylase {So} A4GXE9	0.38
TC265459	Proteolysis	Putative nucleoid DNA-binding protein cnd41, chloroplast {Os} Q69XG4	0.17
TC275361	Proteolysis	Putative serine type endopeptidase inhibitor {Zm} A3FMA3	0.19
TC278353	Proteolysis	Putative serine type endopeptidase inhibitor {Zm} A3FMA3	0.22
TC247886	Proteolysis	Ubiquitin-like protein SMT3 {Os} P55857	0.24
CA452674	Proteolysis	Pyrrolidone carboxyl peptidase-like protein {OS} Q6ERE8	0.29
TC271428	Proteolysis	Serine carboxypeptidase II-1 precursor {Hv} P55747	0.39
TC251128	Proteolysis	Ubiquitin carrier protein {OS} A2X368	0.42
TC192492	Signalling	Peptidyl-prolyl cis-trans isomerase {Os} A2WYA7	0.01
TC261446	Signalling	Putative CRK1 protein {Os} Q93W00	0.05
TC198516	Signalling	Serine/threonine protein kinase {Os} Q304W8	0.07
TC249033	Signalling	Os09g0518700 protein {Os} Q0J0B2	0.09
TC261340	Signalling	Putative C2 domain-containing protein {Os} Q6ZBX9	0.12
AZM4_126821	Signalling	BTH-induced protein phosphatase 1 {Os} AZM4_126821	0.15
TC264850	Signalling	Hexokinase {Os} Q1WM16	0.19
TC192383	Signalling	Putative ethylene-responsive small GTP-binding protein {Os} Q84TA8	0.35
TC261451	Signalling	Peptidyl-prolyl cis-trans isomerase {Os} Q1KL26	0.38
TC268320	Signalling	Serine/threonine kinase {Os} Q304W8	0.38
TC267807	Signalling	S-domain receptor-like protein kinase precursor {Zm} O82103	0.40
TC258109	Signalling	Putative leucine-rich repeat transmembrane protein kinase 1 {Os} Q8H4T2	0.42
TC198868	Stress/Defence	Putative interferon-related protein {Os} Q6ZIP6	0.01
TC197759	Stress/Defence	rRNA N-glycosidase {Zm} Q41851	0.02
TC200016	Stress/Defence	Cold-regulated protein {Hv} Q9FSI8	0.08
TC252043	Stress/Defence	Catalase isozyme 2 {Zm} P12365	0.13
TC268889	Stress/Defence	Heat shock protein 17.9 {Pa} Q40867	0.15
TC254726	Stress/Defence	Derlin-1.2 {ZM} Q4G2J5	0.20
TC265311	Stress/Defence	Os06g0716100 protein {Os} Q0D9H0	0.20
CD219294	Stress/Defence	Desiccation responisve element-binding protein {Sb} A9LNM4	0.25
NP211376|S82315.1|AAB47177.2	Stress/Defence	Pathogenesis-related protein 6 {Zm} Q2XXB4	0.28
TC258760	Stress/Defence	Thioredoxin h1 protein {Zm} Q4W1F7	0.28
BQ485999	Stress/Defence	Heat shock protein 16.9 {Pg} Q40865	0.36
TC269317	Stress/Defence	Copper chaperone homolog CCH {Os} Q9SE04	0.44
TC249270	Transcription	Putative small nuclear ribonucleoprotein polypeptide F {Os} Q6ETN4	0.02
TC217530	Transcription	Homeodomain leucine zipper protein 16 {Os} Q6Q502	0.03
AW147041	Transcription	BTB/POZ domain containing protein {Os} Q2QYP1	0.25
BQ619562	Transcription	Putative MADS-box protein AGL35 {OS} Q5NB09	0.27
TC256140	Transcription	Putative CTV.22 {Os} Q7EZK6	0.33
AZM4_38275	Transcription	Putative DNA-binding protein {At} Q9CA64	0.38
TC250775	Transcription	Two-component response regulator-like PRR73 {OS} A2XFB7	0.38
TC266492	Transcription	HLH transcription factor PTF1 {Zm} Q1KMR1	0.38
TC248635	Translation	Eukaryotic translation initiation factor 2 alpha subunit {Os} Q10MT2	0.02
TC248254	Translation	40S ribosomal protein S10 {Os} Q9AYP4	0.05
CF637160	Translation	60S acidic ribosomal protein P3 {Zm} O24413	0.07
TC248642	Translation	40S ribosomal protein S21 {Zm}P93626	0.10
TC248070	Translation	Putative 60S ribosomal protein L1 {Os} |Q7Y1I5	0.31
TC269396	Translation	Putative 60S ribosomal protein L36 {So} Q5Q0X7	0.35
CD440140	Transport	Aluminum activated citrate transporter {Hv} A7M6U2	0.19
TC247930	Transport	Putative 24 kDa seed maturation protein {Os} Q656J2	0.21
TC263714	Transport	Major facilitator superfamily antiporter {Zm} Q8H6D7	0.22
TC250407	Transport	Mitochondrial import receptor subunit TOM40 {Os} Q10M45	0.24
BM074098	Transport	Amino acid permease family protein {OS} Q2QM92	0.33
TC271875	Transport	Leucine zipper protein-like {Os} Q941X1	0.34
CD976551	Transport	Potassium channel beta chain {Ed} O49847	0.35
TC266533	Transport	Major facilitator superfamily antiporter {Os} Q2RAW4	0.39
TC259019	Transport	Ran {Os} Q9XJ45	0.47
CF007590	Unknown	Putative uncharacterized protein {Os} A2WLZ0	0.01
TC265222	Unknown	Integral membrane protein-like {Os} Q6YT98	0.01
BG458838	Unknown	Expressed protein {Os} Q6AU54	0.02
TC199089	Unknown	Expressed protein {Os} Q10J01	0.05
BM380114	Unknown	OSIGBa0132O24.3 protein {Os} Q01MB4	0.06
TC260121	Unknown	Putative methyl-binding domain protein MBD108 {Zm} Q94IQ7	0.28
TC258671	Unknown	Wali7 protein {Tae} Q43661	0.39

FC/WT corresponds to the fold change in the mutants compared to WT plants for transcripts exhibiting significant variations (Student t-test, $P \leq 0.05$) in their amount.
Genes indicated in bold were used for validation by qRT-PCR.

Table S9b Trancripts exhibiting significant increase (Student t-test, $P \leq 0.05$) in *gln1.4* mutant at the M stage

Identification number	Functional category	Putative annotation	FC/WT
TC270270	C metabolism	Putative alpha-mannosidase {Os} Q8W348	2.52
AZM4_36264	C metabolism	Phosphoglycerate kinase, chloroplast precursor {Tae} S05967	2.85
TC272592	C metabolism	Galactinol synthase 3 {Zm} Q5DVS7	3.88
BM072721	C metabolism	Carbonic anhydrase {Zm} Q41728	5.14
TC261572	C Metabolism	Putative aldose reductase {Zm} A2T1W7	3.87
TC277283	Cell wall	Glycoside hydrolase family 28 protein {Os} Q10B12	22.06
AZM4_1831	Chlorophyll biosynthesis	Uroporphyrinogen decarboxylase, chloroplast precursor {Zm} O81220	2.95
TC260128	Gene silencing	Leafbladeless1 {Zm} A1Y2B7	3.19
TC270501	Genetic information processing	RNA recognition motif family protein expressed {Os} Q10MR0	31.40
CD972391	Lipid metabolism	Lipoxygenase {Zm} Q9LKL4	3.37
TC249711	Lipid metabolism	Beta-ketoacyl-ACP synthase {Os} Q40028	4.96
**TC260211**	**Lipid metabolism**	**Allene oxide cyclase {Zm} Q6RW09**	**7.15**
TC191887	Lipid metabolism	Lipoxygenase {Zm} A1XCH8	23.87
TC249830	Metabolism	Cytochrome P450-like protein {Sb} Q84YE6	2.27
BM500657	Metabolism	Putative cinnamoyl-CoA reductase {OS} Q69U05	2.89
TC255584	Metabolism	Putative iron deficiency protein Ids3 {Os} Q69LD9	3.12
TC252329	Metabolism	Oxidoreductase 2OG-Fe oxygenase family protein expressed {OS} Q336T3	3.31
AZM4_134320	Metabolism	N-hydroxycinnamoyl/benzoyltransferase-like protein {Os} AZM4_134320	3.62
TC279016	Metabolism	putative phosphoethanolamine methyltransferase {Os} Q5SDQ0	3.76
TC271952	Metabolism	Histone acetyltransferase type B catalytic subunit {Zm} Q8LPU4	4.16
TC276041	Metabolism	Putative uncharacterized protein {Os} A2XKC8	4.22
BM500294	Metabolism	Putative NADPH HC toxin reductase {Os} Q7X6N6	4.53
TC253757	Metabolism	Flavonoid 3',5'-hydroxylase {Os} Q10KX4	6.35
TC261752	Metabolism	Histone acetyltransferase type B catalytic subunit {Zm} Q8LPU4	7.64
TC275601	Metabolism	Putative cytochrome P450 {Os} Q3C1U0	7.64
TC266765	Metabolism	Putative iron/ascorbate-dependent oxidoreductase {Os} Q658E2	10.13
TC196270	Metabolism	Ferredoxin VI, chloroplast precursor {Zm} P94044	10.82
TC259563	Metabolism	Putative ubiquinol-cytochrome C reductase complex ubiquinone-binding protein {Os} Q9LDS7	60.19
TC266666	Metabolism	NAD dependent epimerase/dehydratase family protein expressed {Os} Q10CW5	61.18
TC249078	Metabolism	Cytochrome P450 monooxygenase CYP72A27 {Zm} Q8LGN4	68.24
TC266788	Metabolism	Putative NADPH HC toxin reductase {Os} Q7X6N6	101.99
TC260592	mRNA processing	Os05g0437300 protein {Os} Q0DHV7	4.97
TC279101	mRNA processing	mRNA cap guanine-N7 methyltransferase 2 {Os} Q6K833	5.15
TC204807	N Metabolism	Methionine synthase {Zm} Q8W529	2.24
TC250001	N metabolism	Ferredoxin--nitrite reductase, chloroplast precursor {Zm} P17847	4.16
TC260936	Polyamine metabolism	Polyamine oxidase precursor {Zm} O64411	2.92
TC249372	Proteolysis	26S proteasome regulatory particle triple-A ATPase subunit2b {Os} Q8W422	2.69
PUFXO20TBC	Proteolysis	Putative ATP-dependent proteinase; BsgA {Os} Q6Z4A9	3.09
TC249853	Proteolysis	27K protein {Tae} Q7Y1Z2	22.79
**TC199171**	**Proteolysis**	**Putative uncharacterized protein {Os} Q75K52**	**19.96**
TC263491	RNA metabolic process	PolyA polymerase family protein expressed {Zm} Q10S77	2.56
PUCER79TD	RNA metabolic process	Putative hUPF2 {Os} Q6H470	4.40
AZM4_68303	Signalling	F-box protein SNE {At} Q9LUB6	2.34
NP672375	Signalling	Protein kinase {Zm} Q02494	2.48
TC268302	Signalling	Putative Avr9/Cf-9 rapidly elicited protein 264 {Os} Q67UU7	2.67
TC248432	Signalling	Pleckstrin homology (PH) domain-containing protein-like {Os} Q69RJ1	2.74
BG321212	Signalling	Peptidyl-prolyl cis-trans isomerase {OS} A2XL75	2.84
AI065635	Signalling	Protein kinase domain containing protein {OS} Q10LW3	3.36
TC271726	Signalling	Pyruvate dehydrogenase kinase isoform 1 {Zm} O82423	3.96
TC272988	Signalling	Minor allergen alt a7 {Os} A6MZE9	4.16
TC259714	Signalling	Shaggy-like kinase etha {Os} Q5Z7J0	5.16
AZM4_79229	Signalling	Putative MAP3K alpha 1 protein kinase {Os} Q6ESH1	5.78
TC259877	Signalling	Calmodulin {Zm} Q7DLR7	7.89
TC264163	Signalling	Extracellular calcium sensing receptor {Os} Q6YWR8	15.23
TC255710	Signalling	Putative kinase {Os} Q94GW8	46.17
TC248074	Stress/Defence	Expressed protein {OS} Q53PY3	2.79
TC266166	Stress/Defence	Putative uncharacterized protein S126P21.2 {Sb} Q8L434	3.48
BM347819	Stress/Defence	Beta-glucosidase aggregating factor precursor {Zm} Q9LL87	4.25
TC261808	Stress/Defence	Nuclear shuttle interacting protein {At} Q7X9V3	4.65
TC249203	Stress/Defence	Beta-glucosidase aggregating factor 1 {Zm} A0FDH4	4.98
CD968089	Stress/Defence	Os05g0399100 protein {Os} Q0DID5	5.30
TC269399	Stress/Defence	Os05g0399100 protein {Os} Q0DID5	5.67
TC253794	Stress/Defence	17.8 kDa class II heat shock protein {Zm} P24631	8.62
TC274215	Stress/Defence	Glutathione S-transferase GST 15 {Zm} Q9FQC4	8.79
TC261286	Stress/Defence	Metallo-beta-lactamase-like {Os} Q6K9T9	9.03
CF004288	Stress/Defence	Putative uncharacterized protein {OS} A2ZBH5	9.36
TC271734	Structure	Putative ankyrin protein {Os} Q7X6P5	18.02
TC261055	Transcription	OCL3 protein {Zm} Q9LEE8	2.18
AF112150_ZMZMM3-heuer_	Transcription	MADS box protein 3 {Zm} Q9FR84	2.25
TC249903	Transcription	Putative growth-regulating factor 10 {Zm} A5HEG9	2.81
TC259118	Transcription	m23 {Zm} Q84V75	3.00
TC252109	Transcription	High mobility group protein HMGd1 {Zm} O04418	3.50
TC275640	Transcription	Putative WIZZ {Os} Q6EPZ2	4.24
TC252227	Transcription	DNA-binding protein RAV1-like {Os} Q8RZX9	4.39
TC268763	Transcription	WRKY transcription factor {Os} Q8H6W1	4.66
TC270237	Translation	Elongation factor 1 alpha {Zm} Q9M7E6	4.53
TC248224	Translation	40S ribosomal protein S7 {Os} Q10MS5	5.76
TC247883	Translation	40S ribosomal protein S10 {Os} Q9AYP4	6.68
TC249011	Translation	Putative 40S ribosomal protein S26 {Os} P49216	9.02
**TC248617**	**Translation**	**Elongation factor 1-delta 1 {Os} Q40680**	**12.22**
AI065444	Translation	40S ribosomal protein S26 {Os} P49216	21.52
TC272126	Translation	Putative uncharacterized protein {Ps} A9NTN5	46.44
AZM4_21980	Transport	NOD26-like membrane integral protein ZmNIP2-1 {Zm} Q19KC1	2.38
TC254954	Transport	Nitrate transporter {Zm} Q6Y391	2.60
AW181243	Transport	Similar to UPF0014 membrane protein STAR2 {Os} Q9LIX5	2.68
TC262186	Transport	plastidic general dicarboxylate transporter {Zm} Q768R4	3.20
TC250786	Transport	Aquaporin NIP1-1 {Zm} Q9ATN4	3.38
TC273759	Transport	Putative endosomal Vps protein complex subunit {Os} Q8GVS9	3.58
TC211152	Transport	Ras-related protein Rab11C {Os} Q75LE2	3.80
TC248022	Transport	Protein transport protein SEC61 gamma subunit {Os} P38385	4.40
TC264060	Transport	Myosin heavy chain-like {Os} Q5ZB07	6.41
TC276235	Transport	Putative potassium transporter {Os} Q7XIV8	15.85
TC266487	Transposition	Transposon protein putative {OS} Q7XGX1	4.25
TC201872	Unknown	Putative lung seven transmembrane receptor 1 {Os} Q6L517	2.22
TC273286	Unknown	Putative auxin induced protein {Zm} Q6JAC8	3.38
AZM4_40301	Unknown	At5g58020 {At} Q6NQ98	3.88
CF632260_root	Unknown	Putative uncharacterized protein {OS} A2X7W5	4.40
TC260041	Unknown	Putative uncharacterized protein {Mt} Q45NM5	4.54
TC259745	Unknown	Stem-specific protein {So} Q5U7K9	9.73
TC251848	Unknown	Putative BURP domain-containing protein {Os} Q942D4	11.52
TC270417	Unknown	Dormancy/auxin associated protein expressed {Os} Q10LM8	29.62
TC252810	Wax biosynthetic process	Putative CER1 {Os} Q109K1	2.43

FC/WT corresponds to the fold change in the mutants compared to WT plants for transcripts exhibiting significant variations (Student t-test, $P \leq 0.05$) in their amount.
Genes indicated in bold were used for validation by qRT-PCR.

Table S10 Transcripts exhibiting significant difference (Student t-test, *P* ≤ 0.05) in their amount in *gln1.3* mutant both at the V and the M stage

Identification number	Functional category	Putative Annotation	FC at V	FC at M
TC248095	C metabolism	Putative succinate dehydrogenase flavoprotein alpha subunit {Os} Q6ZDY8	0.22	0.16
TC273540	C metabolism	Putative class III chitinase {Os} Q7G8Q1	6.09	0.13
TC272199	Cell wall	Cellulose synthase-3 {Zm} Q9LLI7	0.28	0.11
TC277283	Cell wall	Glycoside hydrolase family 28 protein {Os} Q10B12	11.37	23.09
TC279125	Cell wall	Hydroxyproline-rich glycoprotein-like protein {Os} Q67UA8	12.36	29.29
TC249210	DNA topology	DNA topoisomerase II {Os} A2X8M2	8.01	14.50
TC253670	Lipid metabolism	Very-long-chain fatty acid condensing enzyme CUT1 like protein {At} Q0WWR0	0.15	0.21
TC248075	Lipid metabolism	Acyl-CoA thioester hydrolase-like {Os} Q69MT1	4.87	30.40
TC255020	Lipid metabolism	3-hydroxy-3-methylglutaryl-CoA reductase {Os} O04188	46.64	33.09
TC249231	Metabolism	Putative vacuolar ATP synthase subunit C {Os} Q6L4R5	0.11	0.04
TC251023	Metabolism	Cytochrome P450 {Tae} Q70EV8	0.15	0.09
BM500657	Metabolism	Putative cinnamoyl-CoA reductase {OS} Q69U05	0.35	4.53
AZM4_101226	Metabolism	ferredoxin {Zm} AZM4_101226	5.35	10.19
AZM4_72986	Nucelosome assembly	Histone H2B.2 {Zm} P30756	17.14	26.47
TC249748	Polyamines biosynthetic process	Aminopropyl transferase {Os} Q7XAP2	0.18	0.12
TC271428	Proteolysis	Serine carboxypeptidase II-1 precursor {Hv} P55747	0.21	0.30
TC260199	Proteolysis	Ubiquitin-associated (UBA)/TS-N domain-containing protein-like {Os} Q6K653	0.23	0.19
CF633259	Proteolysis	Putative uncharacterized protein {OS} A2YM57	0.26	0.06
TC247886	Proteolysis	Ubiquitin-like protein SMT3 {Os} P55857	0.34	0.22
TC266142	Proteolysis	Avr9/Cf-9 rapidly elicited protein-like {Os} Q6K5K9	33.18	19.49
TC261195	Proteolysis	26S proteasome regulatory subunit-like protein {Os} Q69Q88	34.37	20.68
**TC199171**	**Proteolysis**	**Putative uncharacterized protein {Os} Q75K52**	**79.56**	**18.49**
TC251967	Secondary metabolism	Chalcone isomerase-like {Os} Q6EQW2	7.37	12.41
TC251968	Secondary metabolism	Chalcone isomerase-like {Os} Q6EQW2	28.12	44.04
TC192492	Signalling	Peptidyl-prolyl cis-trans isomerase {Os} A2WYA7	0.04	0.01
TC271920	Signalling	Putative tubby-like protein {Os} Q6Z2G9	0.11	0.08
BG841682	Signalling	Peptidyl-prolyl cis-trans isomerase{Os} A2WYA7	0.17	0.08
TC271919	Signalling	Putative tubby-like protein {Os} Q6Z2G9	0.17	0.09
TC207366	Signalling	Putative calcium-dependent protein kinase {Os} Q6I587	0.19	0.08
BG874174	Signalling	Galactokinase {PS} Q5ZP80	0.30	0.10
TC250843	Signalling	Putative protein phosphatase-2C {Os} O82470	4.17	6.20
TC249354	Signalling	Putative protein kinase CK2 regulatory subunit CK2B3 {Zm} Q9FQF6	5.08	4.75
TC276810	Signalling	Putative calcium binding protein {Os} Q652U8	22.14	13.63
TC249498	Signalling	Peptidyl-prolyl cis-trans isomerase {Os} A2WYA7	26.76	61.45
TC250399	Signalling	Pyruvate dehydrogenase kinase isoform 2 {Zm} O82424	30.98	157.42
TC264842	Stress/Defence	101 kDa heat shock protein {Tae} Q334I0	0.13	0.05
TC264843	Stress/Defence	101 kDa heat shock protein {Zm} Q9S822	0.17	0.20
TC213439	Stress/Defence	Metallothionein-like protein 1 {Zm} P30571	0.17	0.11
TC252043	Stress/Defence	Catalase isozyme 2 {Zm} P12365	4.71	0.18
TC255599	Stress/Defence	DnaJ protein-like {Os} Q6Z899	5.84	6.04
TC252674	Stress/Defence	Glutathione S-transferase GST 21 {Zm} Q9FQB8	6.17	4.76
CF024860	Stress/Defence	Cystatin II {Zm} Q41825	6.39	3.14
TC264087	Stress/Defence	AvrRpt2-induced protein 2-like {Os} Q6Z2W4	11.97	35.66
TC267841	Transcription	B3 DNA binding domain containing protein expressed {Os} Q2QMT2	0.14	0.15
CF056912	Transcription	Histone deacetylase {Zm} Q9ZTP8	6.74	14.72
TC258948	Translation	putative ribosomal protein S3a {OS} P49397	3.51	0.04
AI987264	Translation	Putative aspartate-tRNA ligase {Os} Q6ZHC3	3.65	3.14
TC248070	Translation	Putative 60S ribosomal protein L1 {Os} |Q7Y1I5	6.38	7.54
TC271736	Translation	Ribosomal protein S21-like protein {Os} Q6YUV4	18.77	32.63
CA403087	Translation	Ribosomal protein S21-like protein {Os} Q6YUV4	20.80	22.52
TC256186	Transport	Putative integral membrane protein {Os} Q688W0	0.02	0.06
TC274040	Transport	Cyclic nucleotide-gated ion channel 4 {Hv} Q4VDN3	0.08	0.03
TC269753	Transport	ATP synthase beta chain, mitochondrial precursor {Zm} P19023	0.10	0.05
TC206341	Transport	Synaptobrevin-like protein {Os} Q69WS1	0.15	0.14
TC261773	Transport	Putative ABC transporter {Os} Q69S79	0.27	0.20
TC271071	Transport	Phosphate/phosphoenolpyruvate translocator protein-like {Ors} Q8S2F4	0.29	0.21
TC248805	Transport	Mannitol transporter putative expressed {Os} Q10QG5	5.72	3.71
TC270254	Transport	ATP synthase subunit D {Os} Q9FT52	10.54	7.18
TC249105	Transport	Ferredoxin 1 {Zm} P27787	13.43	19.36
TC268922	Transport	ADP-ribosylation factor {At} P36397	14.06	12.42
CA402100	Transport	ADP-ribosylation factor-like protein {Nt} Q08IJ1	15.80	30.33
TC253650	Transport	Putative nitrite transporter {Os} Q5Z6P7	22.38	2.77
TC258661	Transport	Syntaxin {Os} Q6YVJ8	31.33	32.93
TC253014	Transposition	Putative AC9 transposase {Zm} P03010	3.00	3.43
T18705	Transposition	Activator-like transposable element {Pg} T18705	4.47	5.24
TC253688	Transposition	Transposase {Zm} Q5UDR1	43.62	29.82
TC256063	Unknown	Mitochondrial transcription termination factor-like protein {Os} Q6K5F5	0.19	0.24
CF006353	Unknown	Putative zinc finger protein {At} O04261	3.18	3.33
CF045733	Unknown	SEC14 cytosolic factor-like {Os} Q6ER71	4.99	12.52
TC260041	Unknown	Putative uncharacterized protein {Mt} Q45NM5	5.14	5.58

FC corresponds to the fold change in the mutants compared to wild type (WT) plants for metabolites exhibiting significant variations (Student t-test, *P* ≤ 0.05) in their amount at the vegetative stage (V) and grain filling stage (M).
Genes indicated in bold were used for validation by qRT-PCR.

Table S11 Transcripts exhibiting significant difference (Student t-test, *P* ≤ 0.05) in their amount in the *gln1.4* mutant both at the V and the M stage

Identification number	Functional category	Putative Annotation	FC at V	FC at M
TC250487	C metabolism	Putative trehalose-6-phosphate synthase {At} Q9C9W6	0.08	0.06
BM072721	C metabolism	Carbonic anhydrase {Zm} Q41728	7.85	5.14
TC249033	Metabolism	Os09g0518700 protein {OS} Q0J0B2	0.14	0.09
TC261275	Metabolism	2-isopropylmalate synthase B {Os} Q2QXY9	0.15	0.03
TC260608	Metabolism	Putative threonine synthase {Os} Q6L4H5	0.31	0.18
TC253728	Metabolism	Phospholipase D alpha 1 {Zm} Q43270	0.33	0.16
TC266765	Metabolism	Putative iron/ascorbate-dependent oxidoreductase {Os} Q658E2	7.80	10.13
TC266666	Metabolism	NAD dependent epimerase/dehydratase family protein {Os} Q10CW5	24.91	61.18
TC266788	Metabolism	Putative NADPH HC toxin reductase {Os} Q7X6N6	107.17	101.99
TC260041	Nucleic acid binding	Uncharacterized protein {Os} Q45NM5	4.38	4.54
TC271428	Proteolysis	Serine carboxypeptidase II-1 precursor {Hv} P55747	0.24	0.39
**TC199171**	**Proteolysis**	**Putative uncharacterized protein {Os} Q75K52**	**67.79**	**19.96**
TC261446	Signalling	Putative CRK1 protein {Os} Q93W00	0.04	0.05
TC261340	Signalling	Putative C2 domain-containing protein {Os} Q6ZBX9	0.20	0.12
TC259714	Signalling	Shaggy-like kinase etha {Os} Q5Z7J0	7.48	5.16
TC259877	Signalling	Calmodulin {Zm} Q7DLR7	8.02	7.89
TC197759	Stress/Defence	rRNA N-glycosidase {Zm} Q41851	0.01	0.02
TC198868	Stress/Defence	Putative interferon-related protein {Os} Q6ZIP6	0.02	0.01
TC265311	Stress/Defence	DnaJ protein homolog-like {Os} Q6YT03	0.07	0.20
TC261286	Stress/Defence	Metallo-beta-lactamase-like {Os} Q6K9T9	10.02	9.03
TC217530	Transcription	Homeodomain leucine zipper protein 16 {Os} Q6Q502	0.06	0.03
CF637160	Translation	60S acidic ribosomal protein P3 {Zm} O24413	0.01	0.07
TC248642	Translation	40S ribosomal protein S21 {Zm} P93626	0.09	0.10
TC248254	Translation	40S ribosomal protein S10 {Os} Q9AYP4	0.18	0.05
TC270237	Translation	Elongation factor 1 alpha {Zm} Q9FYV3	4.51	4.53
TC272126	Translation	Putative uncharacterized protein {Ps} A9NTN5	60.23	46.44
TC259563	Transport	Ubiquinol-cytochrome C reductase complex ubiquinone-binding protein {Os} Q9LDS7	65.76	60.19
BM380114	Transposon	TNP2 {Os} Q948C7	0.03	0.06
TC199089	Unknown	Expressed protein {Os} Q10J01	0.04	0.05

FC corresponds to the fold change in the mutants compared to wild type (WT) plants for metabolites exhibiting significant variations (Student t-test, *P* ≤ 0.05) in their amount at the vegetative stage (V) and grain filling stage (M).
Genes indicated in bold were used for validation by qRT-PCR.

Table S12 Transcripts exhibiting significant difference (Student t-test, *P* ≤ 0.05) in their amount both in the *gln1.3* and *gln1.4* mutant at the V stage

Identification number	Functional category	Putative Annotation	FC 1.3/WT	FC 1.4/WT
TC195262	Metabolism	Isoleucyl-tRNA synthetase-like {Os} Q67WM1	21.73	23.25
TC260041	Nucleic acid binding	Uncharacterized protein {Os} Q45NM5	5.14	4.38
TC260113	Photosynthesis	Probable photosystem II oxygen-evolving complex protein 2 precursor {Nt} Q04126	283.37	200.22
TC261397	Proteolysis	Kelch repeat-containing F-box-like {Os} Q6H8E0	4.81	3.07
TC271428	Proteolysis	Serine carboxypeptidase II-1 precursor {Hv} P55747	0.21	0.24
**TC199171**	**Proteolysis**	**Putative uncharacterized protein {Os} Q75K52**	**79.56**	**67.79**
CF006353	Signalling	Programmed cell death protein 2 {Os} Q10MP9	3.18	3.00
TC259714	Signalling	Shaggy-like kinase etha {Os} Q5Z7J0	6.08	7.48
TC270988	Signalling	Protein phosphatase type-2C {Zm} Q9FQY2	23.61	21.34
TC264476	Transcription	MA3 domain-containing protein {Os} Q10PT6	14.19	5.57
AZM4_8141	Transport	Putative lipid transfer protein {Os} AZM4_8141	0.13	0.09
TC248805	Transport	Mannitol transporter putative expressed {Os} Q10QG5	5.72	9.04
TC274040	Transport	Putative cyclic nucleotide gated ion channel {Os} Q60EI8	0.08	4.73
TC251793	Transport	Peroxisomal biogenesis factor 11 {Mt} A2Q5C8	2.62	2.71
TC248254	Unknown	40S ribosomal protein S10 {Os} Q9AYP4	4.11	0.18
**AI665454**	**Unknown**	**Putative uncharacterized protein {Os} A2Z2L6**	**0.07**	**0.11**

FC corresponds to the fold change in the mutants compared to wild type (WT) plants for metabolites exhibiting significant variations (Student t-test, *P* ≤ 0.05) in their amount at the vegetative stage (V).
Genes indicated in bold were used for validation by qRT-PCR.

Table S13 Transcripts exhibiting significant difference (Student t-test, $P \leq 0.05$) in their amount both in the *gln1.3* and *gln1.4* mutants at the M stage

Identification number	Functional category	Putative Annotation	FC 1.3/WT	FC 1.4/WT
TC208483	C metabolism	Acidic class I chitinase {Zm} Q41794	0.07	0.08
TC277283	Cell wall	Glycoside hydrolase family 28 protein {Os} Q10B12	23.09	22.06
TC272001	Cytoskeleton	Kinesin-like protein {Os} Q7XCW8	0.31	0.29
TC270501	Genetic information processing	RNA recognition motif family protein expressed {Os} Q10MR0	19.74	31.40
TC253728	Lipid metabolism	Phospholipase D alpha 1 {Zm} Q43270	0.16	0.16
**TC260211**	**Lipid metabolism**	**Allene oxide cyclase {Zm} Q6RW09**	**33.17**	**7.15**
TC262102	Metabolism	Putative cytochrome b5 {Bc} Q8H293	0.13	0.17
TC261275	Metabolism	2-isopropylmalate synthase B {Os} Q2QXY9	0.19	0.03
TC263090	Metabolism	Os07g0194500 protein {Os} Q0D7Z6	0.21	0.18
TC261572	Metabolism	Aldose reductase {Zm} A2T1W7	3.48	3.87
TC279016	Metabolism	Putative phosphoethanolamine methyltransferase {Os} Q5SDQ0	4.25	3.76
BM500657	Metabolism	Putative cinnamoyl-CoA reductase {OS} Q69U05	4.53	2.89
TC249078	Metabolism	Cytochrome P450 monooxygenase CYP72A27 {Zm} Q8LGN4	60.42	68.24
TC278353	Proteolysis	Putative serine type endopeptidase inhibitor {Zm} A3FMA3	0.18	0.22
TC247886	Proteolysis	Ubiquitin-like protein SMT3 {Os} P55857	0.22	0.24
TC271428	Proteolysis	Serine carboxypeptidase II-1 precursor {Hv} P55747	0.30	0.39
TC249853	Proteolysis	27K protein {Tae} Q7Y1Z2	9.72	22.79
**TC199171**	**Proteolysis**	**Putative uncharacterized protein {Os} Q75K52**	**18.49**	**19.96**
TC192492	Signalling	Peptidyl-prolyl cis-trans isomerase {Os} A2WYA7	0.01	0.01
AZM4_126821	Signalling	BTH-induced protein phosphatase 1 {Os} AZM4_126821	0.22	0.15
TC192383	Signalling	Putative ethylene-responsive small GTP-binding protein {Os} Q84TA8	0.30	0.35
AI065635	Signalling	Protein kinase domain containing protein {OS} Q10LW3	3.33	3.36
TC272988	Signalling	Minor allergen alt a7 {Os} A6MZE9	8.33	4.16
TC252043	Stress/Defence	Catalase isozyme 2 {Zm} P12365	0.18	0.13
BQ485999	Stress/Defence	Heat shock protein 16.9 {Pg} Q40865	0.23	0.36
CD219294	Stress/Defence	Desiccation responisve element-binding protein {Sb} A9LNM4	0.27	0.25
TC265311	Stress/Defence	Os06g0716100 protein {Os} Q0D9H0	3.69	0.20
TC266166	Stress/Defence	Putative uncharacterized protein S126P21.2 {Sb} Q8L434	4.20	3.48
BM347819	Stress/Defence	Beta-glucosidase aggregating factor precursor {Zm} Q9LL87	8.56	4.25
AZM4_38275	Transcription	Putative DNA-binding protein {At} Q9CA64	0.20	0.38
TC248635	Translation	Eukaryotic translation initiation factor 2 alpha subunit {Os} Q10MT2	0.01	0.02
**TC248617**	**Translation**	**Elongation factor 1-delta 1 {Os} Q40680**	**5.72**	**12.22**
TC248070	Translation	Putative 60S ribosomal protein L1 {Os} |Q7Y1I5	7.54	0.31
TC249011	Translation	Putative 40S ribosomal protein S26 {Os} P49216	12.38	9.02
AI065444	Translation	40S ribosomal protein S26 {Os} P49216	23.65	21.52
CF007590	Unknown	Putative uncharacterized protein {Os} A2WLZ0	0.01	0.01
BG458838	Unknown	Expressed protein {Os} Q6AU54	0.03	0.02
CF632260_root	Unknown	Putative uncharacterized protein {OS} A2X7W5	4.97	4.40
TC260041	Unknown	Putative uncharacterized protein {Mt} Q45NM5	5.58	4.54
TC270417	Unknown	Dormancy/auxin associated protein expressed {Os} Q10LM8	31.93	29.62

FC corresponds to the fold change in the mutants compared to wild type (WT) plants for metabolites exhibiting significant variations (Student t-test, $P \leq 0.05$) in their amount at the grain filling stage (M).

Genes indicated in bold were used for validation by qRT-PCR.

Table S14 Oligonucleotides used for amplification of RNA to obtain DNA fragments for the qRT-PCR used to validate the microarray experiment

Gene identification number	Putative annotation		Primer sequence (5’-3’)
TC270988	Protein phosphatase type-2C {Zm} Q9FQY2	F	TGGTAAGCAGGTGTAGCGAAC
		R	CGGAAGAGGAACAGAACAGC
TC248805	Mannitol transporter {Os} Q10QG5	F	GTGGCATAGTGCGTGTGAGT
		R	GGAGGGATTGAAGAGGGTGT
TC260113	23 kDa polypeptide of photosystem II {Nt} Q04126	F	GATGGGATGCACGCACTAC
		R	TCTCATGGCTGGGAACATC
TC261275	Isopropylmalate synthase B{Os} Q2QXY9	F	GTCCCGAGCACAGAATGAG
		R	TACAAACTGGCAACGAGCAC
TC199171	Uncharacterized protein {Os} Q75K52	F	AAGTTCCCTGGTGAGGTCTG
		R	TGGTTGCTTATTGCCCTTCT
TC248617	Elongation factor delta1 {Os} Q40680	F	AACCTGGATGGCTGGAATAG
		R	GCTGAAATAAACAGAACCGAAC
AI665454	Uncharacterized protein {Os} A2Z216	F	CAGAAAGAAGCAGGGCAAA
		R	AGGAGGAGAAGGACGACGA
TC260211	Allene oxide cyclase {Zm} Q6RW09	F	CAGCCCAGGAACCATCTC
		R	GCAGAAAGAGCAAGTGAATGAA

F = forward, R = reverse.

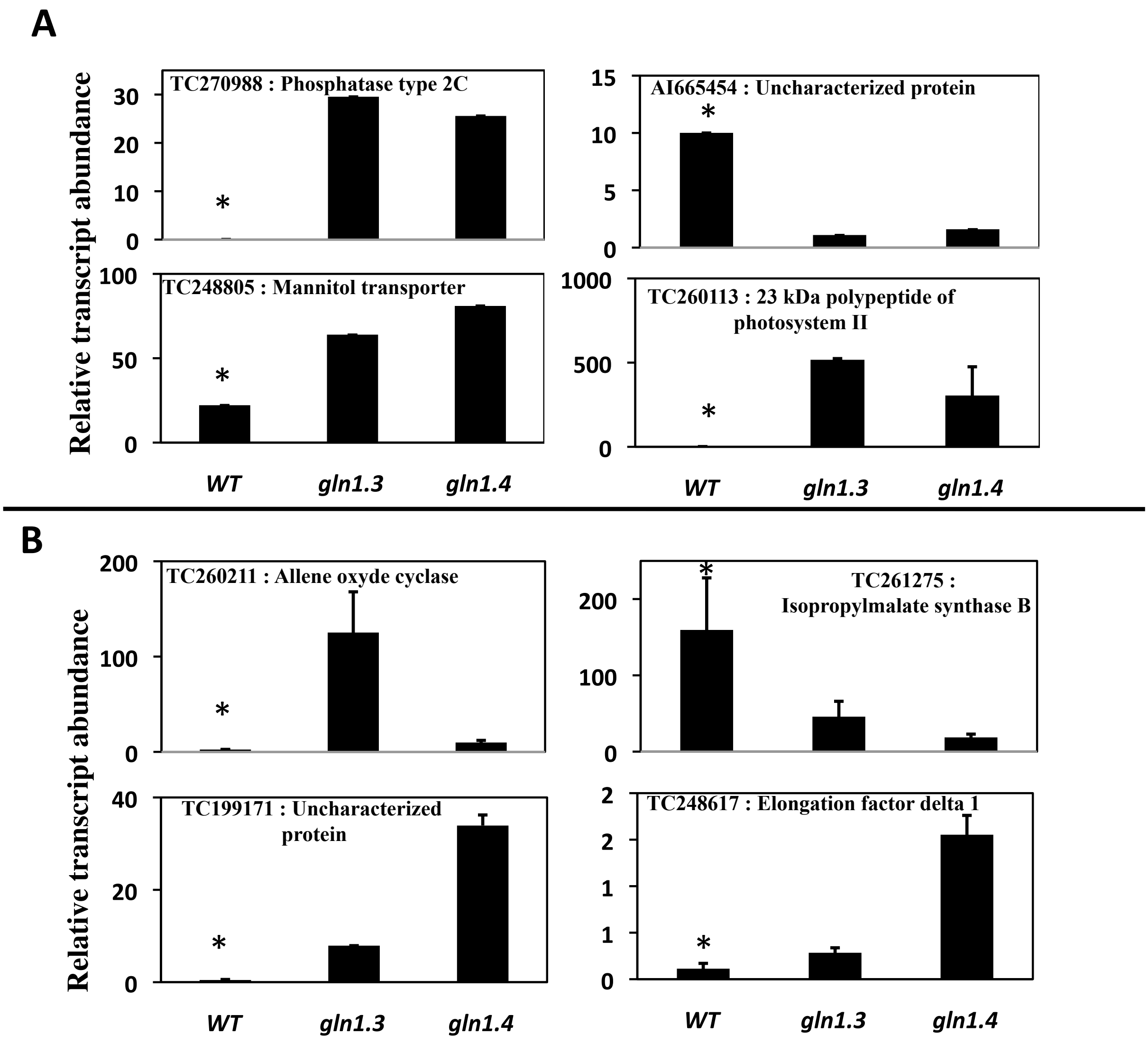

Figure S1 Transcript abundance in the *gln1.3* and *gln1.4* maize (*Zea mays L.*) mutants compared to the wild type (WT) of selected genes. mRNA quantification was performed using qRT-PCR using total RNA samples extracted from leaves. Samples were harvested at the vegetative stage (**A**) and the maturity stage (**B**). Each value was normalized with the relative transcript abundance of the *EF1a* gene. Values are mean ± S.E. of three independent plant samples. The asterisk above the bar indicates significant differences between the WT and the mutants at $P<0.05$, student t-test. The selected genes are indicated in bold in Tables S3 to S14.
